# Design, Synthesis and Biological Evaluation of Conjugates of 3-*O*-Descladinose-azithromycin and Nucleobases against rRNA A2058G- or A2059G-Mutated Strains

**DOI:** 10.3390/molecules28031327

**Published:** 2023-01-30

**Authors:** Xiaotian Lian, Wentian Liu, Bingzhi Fan, Mingjia Yu, Jianhua Liang

**Affiliations:** 1 Key Laboratory of Medical Molecule Science and Pharmaceutical Engineering, School of Chemistry and Chemical Engineering, Beijing Institute of Technology, Beijing 102488, China; 2 Yangtze Delta Region Academy, Beijing Institute of Technology, Jiaxing 314019, China

**Keywords:** antibiotics, drug design, MLS_B_, nucleobase, SARs

## Abstract

Structurally unrelated antibiotics MLS_B_ (macrolide-lincosamide-streptogramin B) compromised with clinically resistant pathogens because of the cross-resistance resulting from the structural modification of rRNA A2058. The structure–activity relationships of a novel 3-*O*-descladinose azithromycin chemotype conjugating with nucleobases were fully explored with the aid of engineered *E. coli* SQ110DTC and SQ110LPTD. The conjugates of macrolides with nucleobases, especially adenine, displayed antibacterial superiority over telithromycin, azithromycin and clindamycin against rRNA A2058/2059-mutated engineered *E. coli* strains at the cost of lowering permeability and increasing vulnerability to efflux proteins against clinical isolates.

## 1. Introduction

Public health has been threatened due to the dramatically surging incidence of resistance in clinical isolates. More than half of the marketed antibacterial drugs serve as protein synthesis inhibitors by targeting 30S or 50S subunits of bacterial ribosomes [1]. Among them, macrolides are widely prescribed to treat community- or hospital-acquired bacterial pneumonia. 

Recently, high-resolution crystal structures of 70S ribosome complexed with erythromycin derivatives revealed the convincible model for a prevailing resistance against structurally unrelated MLS_B_ (macrolide-lincosamide-streptogramin B) antibiotics [2]. The methylated A2058 or A2058 mutation would disrupt the water-mediated hydrogen bonding to the 5-*O*-desosamine of macrolides, and the greatly decreasing affinity could not maintain the tight binding of the antibiotics when nascent peptides egress, leading to high-level resistance. 

**Figure 1:**
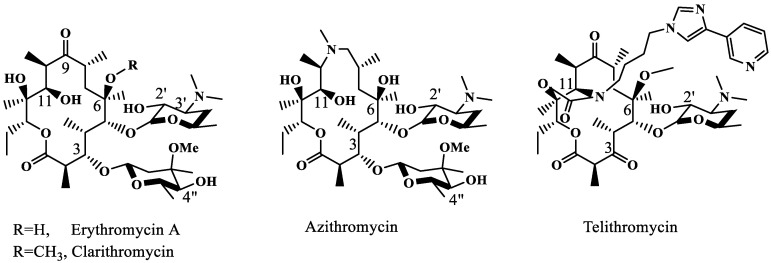
Structures of the 1st-, 2nd- and 3rd-generation erythromycin.

The second-generation semi-synthetic macrolides, clarithromycin and 15-membered azithromycin (Figure 1), failed to afford additional contacts with the ribosome and thus showed no efficacy against erythromycin-resistant bacteria. The third-generation ketolide (replacing 3-*O*-cladinose with 3-keto), telithromycin (Figure 1), succeeded in partially restoring the activities by supplying a secondary π–π interaction between the A752–A2609 base pair and the newly introduced imidazolyl pyridyl group [3,4]. The extended side chain of another ketolide, solithromycin, interacts with the same base pair [5]. However, the affinities of telithromycin or solithromycin to the A2058-methylated or mutated ribosome are too low to allow the usage of these drugs for treating high-level MLS_B_-resistant infections [2].

To strengthen the affinities to the modified ribosome, a variety of mono/bi/fused-heteroaromatic groups were installed onto the end of the linkers derivatized from the 6-OH, 9-keto, 11-OH and 4″-OH of macrolides [6,7]. It was reported that the antibacterial activities of the novel macrolides that were constructed by a total synthesis route still heavily depend on the pendant side chains capable of binding the ribosome [8]. Carbamolides (3-*O*-carbamoyl), acylides (3-*O*-acyl) and alkylides (3-*O*-alkyl) showed a new mode of action, namely π–π interacting with the base pair C2610–G2505 [9,10,11,12]. In 2019, we attempted to introduce some heteroaromatic groups to 3-OH clarithromycin bridged by three-to eight-atom-long spacers. The molecular docking suggested that the most potent compound possessing a three-atom-long spacer shared the same mode of action as that of the known macrolides [13].

Apart from the chemotypes mentioned above, some conjugates have been investigated to achieve dual modes of action [14,15,16]. Quinolones linking to 4″-OH of macrolides have been extensively investigated since 2010, which was well summarized in a recent review [7]. The potent hybrids proved to be non-inhibitors in gyrase-based supercoiling assays [17,18]. In 2020, we reported that quinolones attaching to 6-OH of azithromycin could show moderate activities against *E. coli* DNA gyrase [19].

In continuation of our work, we report here a series of 3-*O*-descladinose azithromycin derivatives substituting 3-*O*-cladinose by seven- to seventeen-atom-length spacers anchored with various nucleobases (adenine, guanine, cytosine, thymine and uracil). There are two reasons to further this work. First, thymine and adenine were linked to 9- or 11-positions of some 3-*O*-cladinose-containing erythromycin derivatives, but the resulting activities, for unknown reasons, are poor [20,21]. The 3-*O*-derivatives of macrolides are not expected to activate the expression of inducible resistance genes, as the presence of 3-*O*-cladinose is required for efficient induction [22], but they failed to afford potency against A2058/2059-methylated or mutated strains. As purines and pyrimidines might form Watson–Crick interactions with rRNA bases, it would be interesting to know whether nucleobases attached at the C3-position of the macrolactone ring of macrolides could restore the activities against the ribosome-mutated strains. Secondly, the modes of action of ribosome-targeting inhibitors are less investigated in the early stage of new macrolides discovery [23] because it is difficult to isolate resistance mutants with alterations in the drug target sites because of the redundancy of rRNA genes in bacteria. To determine the biological function of nucleobases when attached to macrolides, we mutated ribosomal RNA A2058 or A2059 of a public strain *E. coli* SQ110 that has been engineered with only one chromosomal rrn allele [24].

## 2. Results and Discussion

**Scheme 1:**
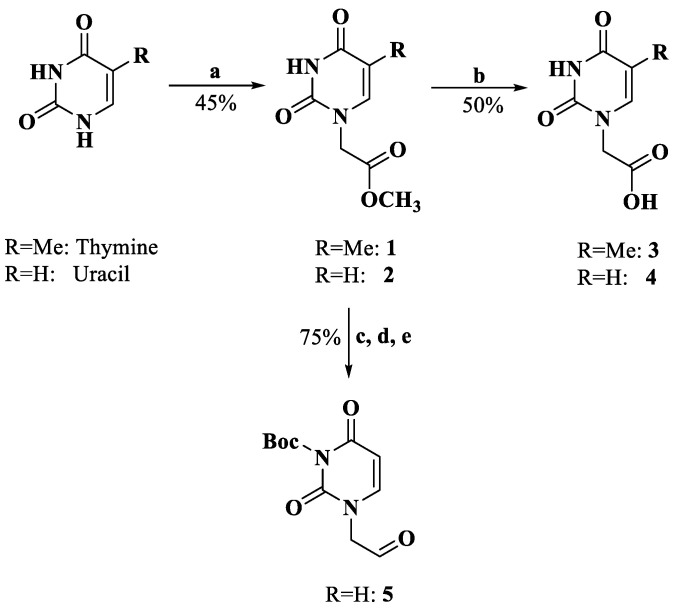
Synthesis of compounds **3–5**. Reagents and conditions: a. DMF, K_2_CO_3_, BrCH_2_COOMe, rt; b. H_2_O, 2M NaOH; 4M HCl, 0 °C; c. CH_2_Cl_2_, DMAP, di-*tert*-butyl dicarbonate, rt; d. THF, MeOH, NaBH_4_, rt; e. CH_2_Cl_2_, Dess–Martin periodinane, rt.

**Scheme 2:**
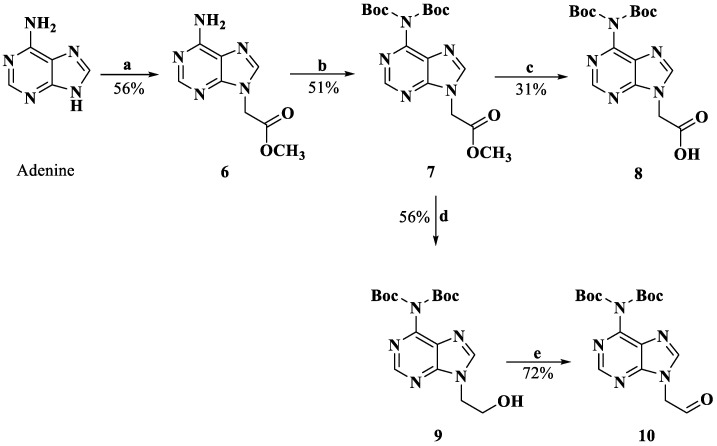
Synthesis of compounds **8** and **10**. Reagents and conditions: a. DMF, K_2_CO_3_, BrCH_2_COOMe, rt; b. CH_2_Cl_2_, DMAP, di-*tert*-butyl dicarbonate, rt; c. MeOH, 2M NaOH; 4M HCl, 0 °C; d. THF, MeOH, NaBH_4_, rt; e. CH_2_Cl_2_, Dess–Martin periodinane, rt.

**Scheme 3:**
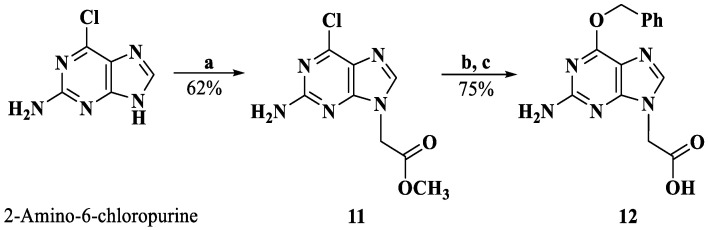
Synthesis of compound **12**. Reagents and conditions: a. DMF, K_2_CO_3_, BrCH_2_COOMe, rt; b. DMF, NaH, benzyl alcohol, rt; c. MeOH, 2M NaOH; 4M HCl, 0 °C.

**Scheme 4:**
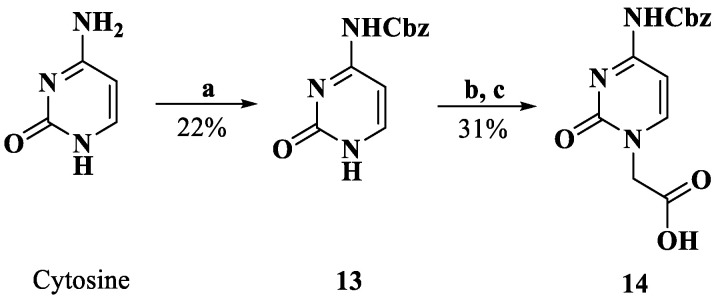
Synthesis of compound **14**. Reagents and conditions: a. Pyridine, Cbz-Cl, 0 °C; b. DMF, K_2_CO_3_, BrCH_2_COOMe, rt; c. H_2_O, 2M NaOH; 4M HCl, 0 °C.

The derivatization of the five nucleobases followed a modified procedure [25]. The nucleobases **3**, **4**, **8**, **12** and **14** were obtained through three steps: a substitution reaction with methyl bromoacetate, protection and saponification, which are outlined in Scheme 1, Scheme 2, Scheme 3 and Scheme 4. To prevent the functional groups at the nucleobases, uracil, adenine, guanine and cytosine were protected by Boc, di-Boc, Bn and Cbz, respectively [26]. The nucleobases **5** and **10** were obtained through the reduction of **2** and **7** to alcohols by using NaBH_4_ and subsequent oxidization to aldehydes with Dess–Martin reagent.

**Scheme 5:**
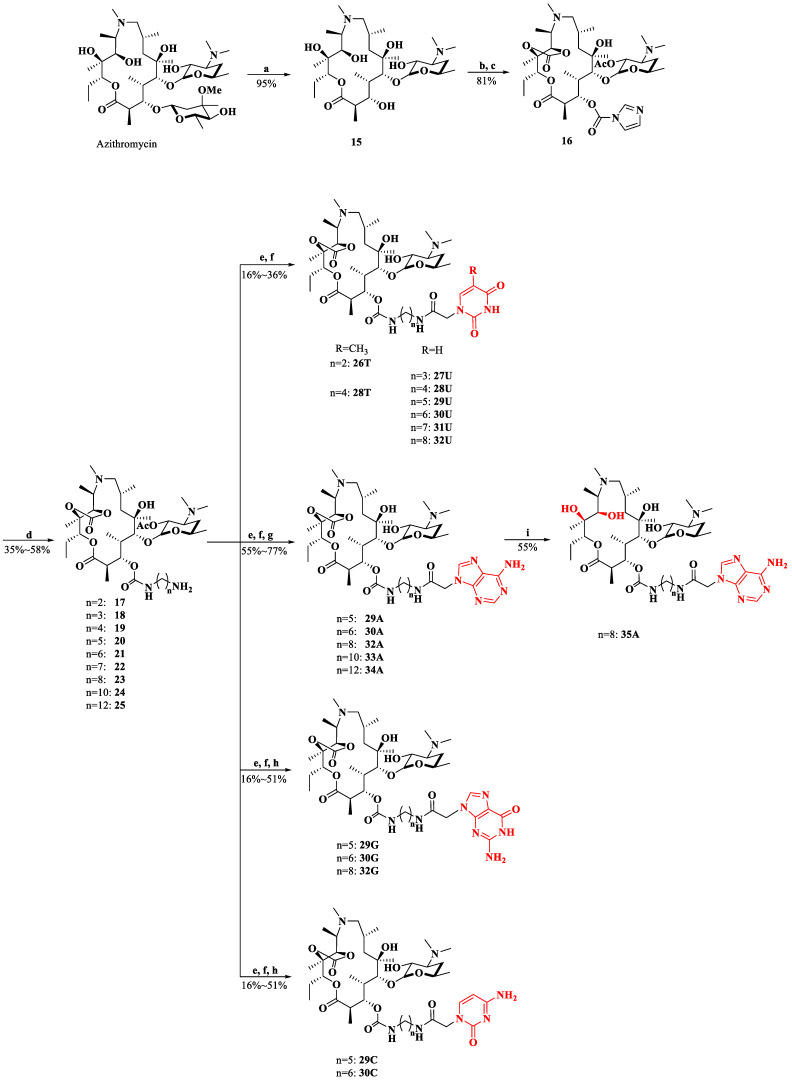
Synthesis of compounds **26–35**. Reagents and conditions: a. EtOH, HCl, 40 °C; b. CH_2_Cl_2_, acetic anhydride, rt; c. CH_2_Cl_2_, 4-dimethylaminopyridine, *N*,*N′*-carbonyldiimdazole, Ar, rt; d. DMF, 1,2-diaminoethane/1,3-diaminopropane/1,4-diaminobutane/1,5-diaminopentane/1,6-diaminohexane/1,7-diaminoheptane/1,8-diaminooctane/1,10-diaminodecane/1,12-diaminododecane, rt. e. CH_2_Cl_2_, compounds **3**, **4**, **8**, **12** or **14**, 1-hydroxybenzotriazole, dicyclohexylcarbodiimide; f. MeOH, 60 °C; g. EtOH, HCl; h. MeOH, H_2_, Pd/C; i. THF:H_2_O = 1:1, LiOH·H_2_O.

Intermediate **16** was prepared from azithromycin in three steps [27]. The general procedure for the synthesis of **26–35** (conjugating with nucleobases A/T/U/G/C) is listed in Scheme 5: introduction of various diamines to the 3-O position, amidization catalyzed by DCC/HOBT, the removal of 2′-acetyl group and the deprotection of the nucleobases [28,29]. Compounds **26T**, **28T** and **27U–32U** were directly obtained without deprotection. Compounds **29A, 30A** and **32A–34A** were synthesized by removing the Boc groups under acidic conditions [30]. In the presence of Pd/C, a hydrogenolysis reaction was carried out to produce the target compounds of **29G–30G**, **32G** and **29C–30C** [31,32]. 11,12-di-OH **35A** was yielded from 11,12-cyclic carbonate **32A** through a treatment with LiOH·H_2_O.

**Scheme 6:**
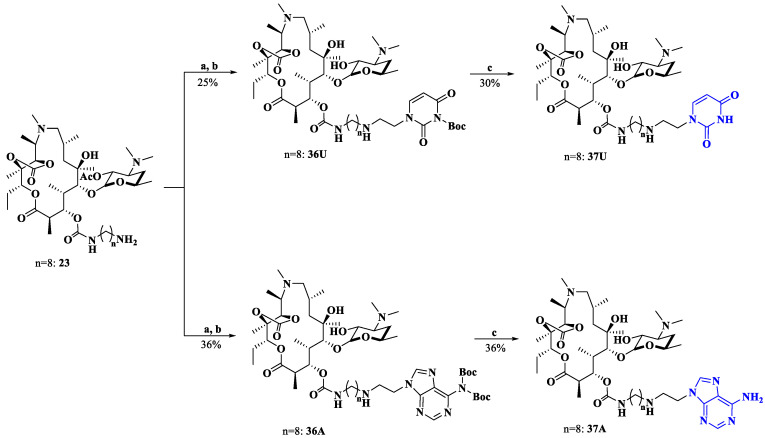
Synthesis of compounds **37U** and **37A**. Reagents and conditions: a. CH_2_Cl_2_, compound **5** or **10**, ZnCl_2_, NaBH(OAc)_3_; b. MeOH, 60 °C; c. EtOH, HCl.

In addition, a more flexible secondary amine (substituting for an amide) side chain spanning from macrolides and nucleobases was designed as a comparator (Scheme 6). In the presence of ZnCl_2_ and NaBH(OAc)_3_, the reaction of **23** with aldehydes **5** or **10** followed by deacetylation yielded the intermediates **36U** and **36A** [33]. The Boc group was then removed to produce the target compounds **37U** and **37A**. All target compounds **26–35** and **37** were structurally validated by using ^1^H NMR, ^13^C NMR and HRMS.

Initially, the MICs of compounds **26–32** were determined against a panel of clinical isolates. The data in Table 1 suggest that the influence of the bases on the activities followed the order of A > G > U > C (**29A**, **29G**, **29U**, **29C**; **30A**, **30G**, **30U**, **30C**), and the activity increased when prolonging the length of the linker (**27U–32U**).

Unexpectedly, the activities of compounds **26–32** were inferior to azithromycin against susceptible and efflux-encoded strains. To further determine whether conjugates **26–32** were vulnerable to outer membrane barriers and efflux pumps, we used the engineered *E. coli* stains SQ110DTC and SQ110LPTD to examine the MICs of compounds **30U**, **30A**, **30G**, **30C**, **32U** and **32A** (Table 2). SQ110 was rendered hypersusceptible to macrolides by deleting the gene encoding the multidrug efflux transporter TolC (strain SQ110DTC) or mutating the *lptD* gene, which renders the outer membrane more permeable (strain SQ110LPTD) [24]. The decreasing MICs against the SQ110 strains in comparison with the reference *E. coli* strain ATCC25922 indicated that the low activities of compounds **26–32** could be attributed to their poor permeability and vulnerability to efflux. Among them, compounds **30A** and **32A,** which have adenine groups, were the most active, which was in agreement with the SARs against the clinical isolates, as shown in Table 1. The presence of the uracil base was inferior to the adenine (**30U** vs. **30A**, **32U** vs. **32A**). None of the compounds were active against the A2058/A2059-mutated SQ110 stains (Table 2).

It was reported that menadione (VK_3_) could inhibit the efflux pumps and alter the fluidity of the bacterial membrane of *S. aureus* [34]. Thus, compound **32A**, in combination with the usage of menadione (VK_3_) at concentrations of MIC/4 or MIC/8, exhibited better activities against *S. aureus* than 3-*O*-descladinose-3-OH azithromycin while much lower activities of **32A** than azithromycin (Table 3), which suggested that the introduction of nucleobases is detrimental to the resulting activities of the conjugates against the clinical isolates.

With the most potent compound, **32A**, in hand, the analogs of **32A** were then screened, as shown in Table 4. The varying lengths of the linker (**32A**, **33A**, **34A**) revealed that the homolog of **33A** was more potent than **32A** and **34A**. Replacing the base A with G/U (**32A** vs. **32G**; **37A** vs. **37U**) and removing cyclic carbonation at 11,12-OH (**32A** vs. **35A**) were not favorable for activities. The conversion of a rigid linker (amide functionality) to a flexible one (amine) resulted in two- to four-fold higher activities (**32A** vs. **37A**).

**Figure 2:**
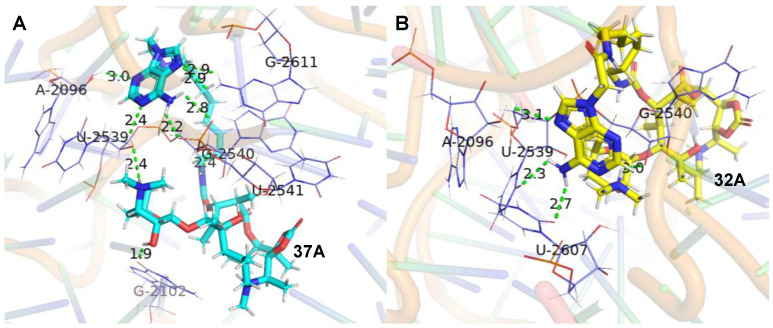
Interactions of the ligands with 50S ribosomal subunit (PDB ID: 1YHQ). (**A**). Interaction between **37A** (cyan) and 50S ribosomal subunit. (**B**). Interaction between **32A** (yellow) and 50S ribosomal subunit. The green dashed lines represent conventional hydrogen bonds. The labeled bases are colored purple and shown in licorice by PyMOL [38].

The molecular docking studies of the ligands (**37A** and **32A**) against the G2099A mutant *Haloarcula Marismortui* 50S ribosomal subunit (PDB ID: 1YHQ [35]) were performed utilizing Autodock VINA in YASARA (YASARA Biosciences GmbH, Vienna, VIE, AUT) [36,37]. The suggested binding modes of **37A** and **32A** (Figure 2) indicated that hydrogen bonds were formed between the adenines of the conjugates and the surrounded ribosomal nucleobases (Tables S1 and S2), which accounted for the better activities of **37A** and **32A** than azithromycin against A2058/2059-mutated SQ110 strains. Meanwhile, higher binding affinities were determined for **37A** than **32A** (Table 5), which was in agreement with the higher activities of **37A** than those of **32A** (Table 4).

**Figure 3:**
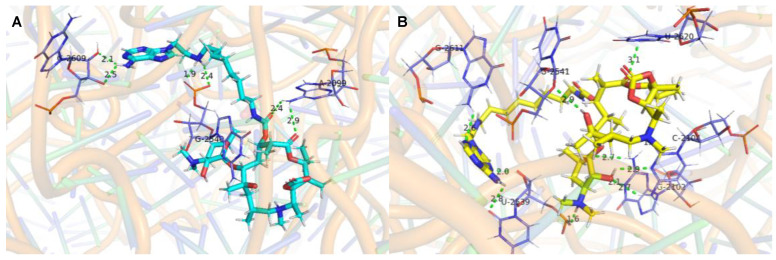
Interactions of the ligands with 50S ribosomal subunit (PDB ID: 1YHQ) after molecular dynamic simulation. (**A**). Interaction between **37A** (cyan) and 50S ribosomal subunit. (**B**). Interaction between **32A** (yellow) and 50S ribosomal subunit. The green dashed lines represent conventional hydrogen bond. The labeled bases are colored purple and shown in licorice by PyMOL.

The adenine, 3′-N(CH_3_)_2_ of the desosamine, the carbamoyl group in **37A** formed six conventional hydrogen bonds with both sugar and phosphate groups in the RNA backbone linked to A2096/ U2539/ G2540/ U2541 (Table S1). The adenine and 2′-OH of the desosamine in **37A** formed three conventional hydrogen bonds with the 2-NH_2_ of G2611 and O6 of G2102. The adenine and 3′-N(CH_3_)_2_ of the desosamine in **32A** formed two conventional hydrogen bonds with the sugar group in the RNA backbone linked to A2096 and G2540. The adenine in **32A** formed two conventional hydrogen bonds with the O2 of U2607 and U2539 (Table S2). Therefore, it revealed that the **37A** bound tightly to the backbone of the G2099A large ribosomal subunit. The reliability of the models constructed from protein–ligand docking was verified by using molecular dynamics (MD) simulations. Both methods yielded similar complex structures. Quantitatively, the RMSD values of the structures from the MD and docking are small (Figure S2), which indicates that the structural variation is minor. Interestingly, we found that after the molecular dynamic simulation, the 6-NH_2_ of compound **37A**’s adenine formed two hydrogen bonds with the sugar in the RNA backbone linked to G2609. The -NH- of the linker chains formed two hydrogen bonds with the phosphate groups of G2540, and the -C=O- of the linker chains and the backbone of macrolides formed two hydrogen bonds with 6-NH_2_ of A2099 (Figure 3A, Table S3). The 6-OH of compound **32A** formed a hydrogen bond with the sugar in the RNA backbone linked to U2541. The 2′-OH formed two hydrogen bonds with the 4-NH_2_ and N3 of C2104 and a hydrogen bond with 1-NH of G2102. Moreover, the -N-CH_3_ and O6 of the backbone of macrolides formed a hydrogen bond with the O2 of C2104 and 2-NH_2_ of G2102, respectively. The 3′-N(CH_3_)_2_ of the desosamine, 6-NH_2_ and N7 of adenine formed a hydrogen bond with the phosphate groups, O1 and the sugar in the RNA backbone linked to G2539, respectively. The -C=O- of the backbone of macrolides formed a hydrogen bond with 3-NH of U2620, and the N3 of adenine formed a hydrogen bond with the 2-NH_2_ of G2611 (Figure 3B, Table S4).

## 3. Materials and Methods

### 3.1. Synthetic Procedures

All of the solvents and reagents were obtained from commercial sources (Innochem, Beijing, China; Bidepharm, Shanghai, China; J&K scientific, Beijing, China) and used without further purification unless otherwise noted. The reactions were monitored by using thin-layer chromatography (TLC, Qingdao Ocean Chemical Co., Ltd., Qingdao, China) with silica gel HSGF254 precoated plates (0.2 mm), and the compounds were visualized under UV light (λ = 254 or 365 nm) and/or stained with iodine. Column chromatography was performed on silica gel (100–200 mesh). ^1^H and ^13^C spectra were taken in CDCl_3_, MeOH-*d_4_* or DMSO-*d_6_* on Bruker Ascend 400 MHz or Ascend 700 MHz spectrometers (Bruker, Massachusetts, Germany) with tetramethylsilane (TMS) as an internal standard. High-resolution mass spectra (HRMS) were obtained with an Agilent Q-TOF 6520 (Agilent Technologies Inc., Santa Clara, CA, USA). The spectra of compounds are shown in Table S5.

#### 3.1.1. Methyl Thymin-1-ylacetate (**1**)

To a suspension of thymine (1.000 g, 7.93 mmol) and K_2_CO_3_ (1.100 g, 7.93 mmol) in dry DMF, methyl bromoacetate was added (0.85 mL, 7.93 mmol), and the mixture was stirred overnight. Then, the mixture was filtered, the solid residue was cooled to 0 °C and then treated with water and 4M HCl. The precipitate was collected by filtration and washed with water to afford compound **1** (0.700 g, 3.54 mmol, 44.6%). HRMS (ESI) (M + H)^+^ *m*/*z* 199.0717, calcd for C_8_H_11_N_2_O_4_^+^ 199.0713. ^1^H NMR (DMSO-*d_6_*, 400 MHz) δ: 10.13 (s, 1 H, -NH-), 7.51 (s, 1 H, H-6), 4.49 (s, 2 H, -CH_2_-), 3.69 (s, 3 H, -O-CH_3_), 1.76 (s, 3 H, 5-CH_3_).

#### 3.1.2. Thymin-1-ylacetic Acid (**3**)

Compound **1** (0.700 g, 3.54 mmol) was treated with water (3 mL) and 2M NaOH (3 mL). The mixture was boiled for 10 min, and then cooled to 0 °C and treated with 4M HCl to adjust the pH to 3. After stirring for 30 min, the solid was filtered and washed with water to afford compound **3** (0.325 g, 1.77 mmol, 50.0%). HRMS (ESI) (M − H)^−^ *m*/*z* 183.0414, calcd for C_7_H_7_N_2_O_4_^−^ 183.0411. ^1^H NMR (DMSO-*d_6_*, 400 MHz) δ: 13.08 (s, 1 H, -COOH), 11.34 (s, 1 H, -NH-), 7.49 (s, 1 H, H-6), 4.36 (s, 2 H, -CH_2_-), 1.75 (s, 3 H, 5-CH_3_).

#### 3.1.3. Uracil-1-ylacetic Acid (**4**)

To a suspension of uracil (1.000 g, 8.92 mmol) and K_2_CO_3_ (1.356 g, 8.92 mmol) in dry DMF, methyl bromoacetate was added (0.96 mL, 8.92 mmol), and the mixture was stirred overnight. The mixture was filtered, and the solid residue was cooled to 0 ºC and treated with water and 4M HCl. The precipitate was collected by filtration and washed with water. The solid was treated with water (3 mL) and 2M NaOH (3 mL), and then the mixture was boiled for 10 min. The mixture was cooled to 0 °C and treated with 4M HCl to adjust the pH to 3. After stirring for 30 min, compound **4** (0.287 g, 1.69 mmol, 18.9%) was filtered and washed with water. HRMS (ESI) (M + H)^+^ *m*/*z* 171.0398, calcd for C_6_H_7_N_2_O_4_^+^ 171.0400. ^1^H NMR (DMSO-*d_6_*, 400 MHz) δ: 13.12 (s, 1 H, -COOH), 11.34 (s, 1 H, -NH-), 7.61 (d, *J* = 8.0 Hz, 1 H, H-6), 5.59 (d, *J* = 7.6 Hz, 1 H, H-5), 4.41 (s, 2 H, -CH_2_-).

#### 3.1.4. (*N*^3^-(tert-butyloxy carbonyl)uracil-1-yl)acetaldehyde (**5**)

Compound **2** (2.313 g, 12.57 mmol) was dissolved in dry CH_2_Cl_2_, and then DMAP (0.566 g, 12.57 mmol) and di-*tert*-butyl dicarbonate (1.000 g, 15.08 mmol) were added to the solution over a period of 15 min at 0 °C. The cooling was discontinued, and the solution was stirred for 8 h. Subsequently, the mixture was treated with saturated NH_4_Cl repeatedly. After stirring for 30 min, the phases were separated, and the organic phase was washed with water and brine. The organic layer was concentrated and purified through column chromatography (CH_2_Cl_2_/EtOH = 10:0.1). Then, the resulting compound (0.400 g, 1.41 mmol) was dissolved in dry THF/MeOH (10:1), and NaBH_4_ (0.106 g, 2.82 mmol) was added to the solution. After stirring for 1 h at room temperature, the mixture was washed with water and brine, and the organic layer was concentrated. The resulting compound (0.218 g, 0.85 mmol) and Dess–Martin periodinane (0.396 g, 0.93 mmol) were dissolved in dry CH_2_Cl_2_. The solution was stirred for 1 h at room temperature and then washed with water and brine. The organic layer was concentrated and purified through column chromatography (CH_2_Cl_2_/EtOH = 10:0.3) to afford compound **5** (0.161 g, 0.64 mmol, 75.3%). ^1^H NMR (MeOD-*d_4_*, 400 MHz) δ: 9.72 (s, 1 H, -CHO), 7.03 (d, *J* = 8.0 Hz, 1 H, H-6), 5.83 (d, *J* = 8.0 Hz, 1 H, H-5), 4.64 (d, *J* = 4.8 Hz, 2 H, -CH_2_-), 1.63 (s, 9 H, -Boc).

#### 3.1.5. Methyl Adenin-9-ylacetate (**6**)

To a suspension of adenine (1.000 g, 7.40 mmol) and K_2_CO_3_ (1.227 g, 8.88 mmol) in dry DMF, methyl bromoacetate was added (1.60 mL, 14.80 mmol). After stirring at room temperature for 5 h, the mixture was filtered, and the solid residue was treated with ethyl acetate, resulting in recrystallization. The solid was filtered off and followed by recrystallization using MeOH to yield 0.807 g (4.18 mmol, 56.5%) of white product. HRMS (ESI) (M + H)^+^ *m*/*z* 194.0670, calcd for C_7_H_8_N_5_O_2_^+^ 194.0673. ^1^H NMR (DMSO-*d_6_*, 400 MHz) δ: 8.13 (s, 1 H, H-2), 8.12 (s, 1 H, H-8), 7.27 (s, 2 H, -NH_2_), 5.09 (s, 2 H, -CH_2_-), 3.70 (s, 3 H, -O-CH_3_).

#### 3.1.6. Methyl (*N*^6^-(di-tert-butyloxy carbonyl)adenin-9-yl)acetate (**7**)

Compound **6** (0.807 g, 4.18 mmol) was dissolved in dry CH_2_Cl_2_, and then DMAP (1.532 g, 12.54 mmol) and di-*tert*-butyl dicarbonate (2.737 g, 12.54 mmol) were added to the solution over a period of 15 min at 0 °C. The cooling was discontinued, and the solution was stirred overnight. Subsequently, the mixture was treated with saturated NH_4_Cl repeatedly. After stirring for 30 min, the phases were separated, and the organic phase was washed with water and brine. The organic layer was concentrated and purified through column chromatography (CH_2_Cl_2_/EtOH = 10:0.15) to obtain compound **7** (0.795 g, 2.15 mmol, 51.4%). ^1^H NMR (CDCl_3_, 400 MHz) δ: 8.86 (s, 1 H, H-2), 8.16 (s, 1 H, H-8), 5.08 (s, 2 H, -CH_2_-), 3.81 (s, 3 H, -CH_3_), 1.45 (s, 18 H, -N(Boc)_2_).

#### 3.1.7. (*N*^6^-(di-tert-butyloxy carbonyl)adenin-9-yl)acetic Acid (**8**)

Compound **7** (0.795 g, 2.15 mmol) was mixed with MeOH, and 2M NaOH (5 mL) was added. After stirring for 30 min, CH_2_Cl_2_ and water were added to the solution for extraction. The water phase was adjusted to pH 3.0 with 4M HCl at 0 °C. Target compound **8** (0.507 g, 1.29 mmol, 30.9%) was filtered and washed with water. HRMS (ESI) (M + H)^+^ *m*/*z* 394.1713, calcd for C_7_H_8_N_5_O_2_^+^ 394.1721. ^1^H NMR (CDCl_3_, 400 MHz) δ: 8.77 (s, 1 H, H-2), 8.29 (s, 1 H, H-8), 4.86 (s, 2 H, -CH_2_-), 1.42 (s, 18 H, -N(Boc)_2_).

#### 3.1.8. (*N*^6^-(di-tert-butyloxy carbonyl)adenin-9-yl)ethanol (**9**)

Compound **7** (0.500 g, 1.23 mmol) was dissolved in dry THF/MeOH (10:1), and NaBH_4_ (0.093 g, 2.45 mmol) was added to the solution. The mixture was stirred for 1 h at room temperature and then washed with water and brine. The organic layer was concentrated and purified through column chromatography (CH_2_Cl_2_/EtOH = 10:0.5) to afford compound **9** (0.260 g, 0.69 mmol, 56.0%). ^1^H NMR (CDCl_3_, 400 MHz) δ: 8.84 (s, 1 H, H-2), 8.14 (s, 1 H, H-8), 4.41 (t, *J* = 4.8 Hz, 2 H, -CH_2_-), 4.01 (t, *J* = 4.8 Hz, 2 H, -C**H_2_**-OH), 3.57 (s, 1 H, -OH), 1.46 (s, 18 H, -N(Boc)_2_).

#### 3.1.9. (*N*^6^-(di-tert-butyloxy carbonyl)adenin-9-yl)acetaldehyde (**10**)

Compound **9** (0.260 g, 0.69 mmol) and Dess–Martin periodinane (0.349 g, 0.83 mmol) were dissolved in dry CH_2_Cl_2_. The solution was stirred for 1 h at room temperature, then washed with water and brine. The organic layer was concentrated and purified through column chromatography (CH_2_Cl_2_/EtOH = 10:0.3) to afford compound **10** (0.189 g, 0.50 mmol, 72.0%). HRMS (ESI) (M + H)^+^ *m*/*z* 378.1768, calcd for C_17_H_24_N_5_O_5_^+^ 378.1772. ^1^H NMR (CDCl_3_, 400 MHz) δ: 9.86 (s, 1 H, -CHO), 8.87 (s, 1 H, H-2), 8.11 (s, 1 H, H-8), 5.22 (s, 2 H, -CH_2_-), 1.48 (s, 18 H, -N(Boc)_2_).

#### 3.1.10. Methyl (2-Amino-6-chloropurin-9-yl)acetate (**11**)

To a suspension of 2-amino-6-chloropurine (1.000 g, 5.91 mmol) and K_2_CO_3_ (2.450 g, 17.73 mmol) in DMF, methyl bromoacetate was added (0.76 mL, 7.09 mmol), and the mixture was stirred for 5 h. Water was added, the suspension was filtered, and the solid was dried to afford intermediate **11** (0.880 g, 3.65 mmol, 62%).

#### 3.1.11. (2-Amino-6-(benzyloxy)purin-9-yl)acetic acid (**12**)

NaH (0.730 g, 18.25 mmol) was dissolved in benzyl alcohol (10 mL) for 2 h at 0 °C, a solution of compound **11** (0.880 g, 3.65 mmol) in DMF was slowly added, and the resultant suspension was stirred overnight at room temperature. Then, 1M NaOH was added, and the solution was washed with ethyl acetate. The water phase was acidified to pH 3 with 4M HCl and extracted with ethyl acetate repeatedly. The combined organic phases were washed with brine and concentrated. The residue was recrystallized from EtOH (yield 74.8%). ^1^H NMR (DMSO-*d_6_*, 400 MHz) δ: 7.84 (s, 1 H, H-8), 7.52–7.35 (m, 5 H, -phenyl), 6.50 (s, 2 H, -NH_2_), 5.50 (s, 2 H, -O-CH_2_-), 4.82 (s, 2 H, -CH_2_-).

#### 3.1.12. *N*^4^-(Benzyloxycarbonyl)cytosine (**13**)

Cbz-Cl (2.54 mL, 18.0 mmol) was added dropwise over a suspension of cytosine (1.000 g, 9.0 mmol) in pyridine (10 mL) at 0 °C. The mixture was stirred overnight. Water was added, and the pH was adjusted to 1 with 4M HCl. The resultant white precipitate was filtered off, washed with water, and dried to afford **13** (0.510 g, 2.08 mmol, 22%). ^1^H NMR (DMSO-*d_6_*, 400 MHz) δ: 7.79 (d, *J* = 7.2 Hz, 1 H, H-6), 7.40–7.34 (m, 5 H, -phenyl), 6.91 (d, *J* = 7.2 Hz, 1 H, H-5), 5.17 (s, 2 H, -O-CH_2_-).

#### 3.1.13. *N*^4^-(Benzyloxycarbonyl)cytosin-1-yl)acetic Acid (**14**)

To a suspension of compound **13** (0.510 g, 2.08 mmol) and K_2_CO_3_ (0.288 g, 2.08 mmol) in dry DMF, methyl bromoacetate was added (0.23 mL, 2.08 mmol), and the mixture was stirred overnight. The mixture was filtered, and the solid residue was treated with water and 4M HCl at 0 °C. The precipitate was collected through filtration and treated with water (3 mL) and 2M NaOH (3 mL). After stirring for 30 min, the solution was cooled to 0 ºC and treated with 4M HCl to adjust the pH to 3. The mixture was filtered and washed with water to obtain a white solid, **14** (0.198 g, 0.65 mmol, 31.4%). HRMS (ESI) (M + H)^+^ *m*/*z* 304.0954, calcd for C_14_H_14_N_3_O_5_^+^ 304.0928. ^1^H NMR (DMSO-*d_6_*, 400 MHz) δ: 8.04 (d, *J* = 7.2 Hz, 1 H, H-6), 7.41–7.35 (m, 5 H, -phenyl), 7.02 (d, *J* = 6.8 Hz, 1 H, H-5), 5.20 (s, 1 H, -O-CH_2_-), 4.53 (s, 2 H, -CH_2_-).

#### 3.1.14. 3-*O*-descladinosyl-3-OH azithromycin (**15**)

To a solution of azithromycin (20.000 g, 26.7 mmol) in ethanol (35 mL), 1 M hydrochloric acid was slowly dripped into the reaction system. After stirring for 1 h at 40 °C, the pH was adjusted to 10 with NH_3_·H_2_O, and then CH_2_Cl_2_ was added for extraction. The organic phase was washed with water and brine and dried to afford compound **15** (15.500 g, 26.2 mmol, 98.1%) as a white solid. HRMS (ESI) (M + H)^+^ *m*/*z* 591.4194, calcd for C_30_H_59_N_2_O_9_^+^ 591.4215. ^1^H NMR (MeOD-*d_4_*, 400 MHz) δ: 4.92 (dd, *J* = 10.8, 2.4 Hz, 1 H, H-13), 4.68 (d, *J* = 7.2 Hz, 1 H, H-1′), 3.63–3.60 (m, 2 H, H-3, H-11), 3.57–3.51 (m, 2 H, H-5, H-5′), 3.26 (dd, *J* = 10.0, 7.6 Hz, 1 H, H-2′), 2.84 (q, *J* = 10.0, 7.2 Hz, 1 H, H-10), 2.68–2.57 (m, 2 H, H-2, H-3′), 2.50–2.46 (m, 1 H, H-9a), 2.35 (s, 3 H, N-CH_3_), 2.33 (s, 6 H, -N(CH_3_)_2_), 2.24–2.22 (m, 1 H, H-4), 2.17–2.11 (m, 1 H, H-9b), 1.90–1.84 (m, 2 H, H-8, H-14eq), 1.74–1.65 (m, 2 H, H-4′a, H-7a), 1.58–1.42 (m, 2 H, H-7b, H-14ax), 1.25 (s, 3 H, 6-CH_3_), 1.24–1.20 (m, 7 H, H-4′b, 2-CH_3_, 4-CH_3_), 1.11 (d, *J* = 6.8 Hz, 3 H, 5′-CH_3_), 1.08 (s, 3 H, 12-CH_3_), 1.00 (d, *J* = 7.6 Hz, 3 H, 10-CH_3_), 0.92 (d, *J* = 7.2 Hz, 3 H, 8-CH_3_), 0.88 (t, *J* = 7.2 Hz, 3 H, 15-CH_3_).

#### 3.1.15. 2′-*O*-acetyl-3-*O*-descladinosyl-3-*O*-imidazolylcarbonyl azithromycin-11,12-cyclic carbonate (**16**)

To a stirred solution of compound **15** (15.500 g, 26.2 mmol) in CH_2_Cl_2_ (150 mL), acetic anhydride was added (3.22 mL, 34.1 mmol). The mixture was stirred at room temperature for 1 h and quenched by the addition of saturated NaHCO_3_. The remaining acetic anhydride was removed by washing thoroughly with saturated NaHCO_3_. Then, the organic phase was washed with water and brine and concentrated to yield 2′-*O*-acetyl-3-*O*-descladinosylazithromycin (15.900 g, 25.1 mmol, 95.7%) as a white solid.

The resulting compound (15.900 g, 25.1 mmol) was dissolved in dry CH_2_Cl_2_ (10 mL), then 4-dimethylaminopyridine (6.131 g, 50.2 mmol) and *N, N*-carbonyl diimidazole (12.176 g, 75.3 mmol) were added to this solution under Ar. The reaction was stirred at room temperature for 12 h and was washed with saturated NH_4_Cl solution, water and brine. The organic phase was evaporated under reduced pressure, and then an appropriate amount of ethyl acetate was added. The mixture was stirred in an ice bath for 2 h and filtered to afford a white solid, **16** (15.928 g, 21.2 mmol, 84.6%). HRMS (ESI) (M + H)^+^ *m*/*z* 753.4280, calcd for C_37_H_61_N_4_O_12_^+^ 753.4278. ^1^H NMR (CDCl_3_, 400 MHz) δ: 8.22 (s, 1 H, H-imidazole), 7.51 (t, *J* = 1.5 Hz, 1 H, H-imidazole), 7.14 (dd, *J* = 1.7, 0.9 Hz, 1 H, H-imidazole), 6.62 (s, 1 H, 6-OH), 5.67 (dd, *J* = 9.2, 3.3 Hz, 1 H, H-3), 5.07 (dd, *J* = 10.8, 2.4 Hz, 1 H, H-13), 4.69 (dd, *J* = 10.6, 7.5 Hz, 1 H, H-2′), 4.59 (s, 1 H, H-11), 4.02 (d, *J* = 7.5 Hz, 1 H, H-1′), 3.60 (d, *J* = 2.7 Hz, 1 H, H-5), 3.21–3.12 (m, 1 H, H-2), 2.86 (q, *J* = 6.6 Hz, 1 H, H-10), 2.69–2.60 (m, 1 H, H-5′), 2.40–2.34 (m, 1 H, H-9a), 2.34–2.29 (m, 1 H, H-3′), 2.23–2.30 (m, 4 H, N-CH_3_, H-4), 2.20 (s, 6 H, -N(CH_3_)_2_), 2.09 (s, 3 H, 2′-*O*-CO-CH_3_), 2.07–2.03 (m, 1 H, H-9b), 1.94–1.82 (m, 2 H, H-7a, H-14eq), 1.79–1.67 (m, 1 H, H-8), 1.64–1.56 (m, 1 H, H-14ax), 1.55–1.48 (m, 2 H, H-7b, H-4′a), 1.43 (s, 3 H, 12-CH_3_), 1.31–1.26 (m, 1 H, H-4′b), 1.25 (d, *J* = 7.1 Hz, 3 H, 5′-CH_3_), 1.22 (s, 3 H, 6-CH_3_), 1.11 (d, *J* = 6.1 Hz, 3 H, 10-CH_3_), 1.09–1.04 (m, 6 H, 2-CH_3_, 4-CH_3_), 0.94–0.88 (m, 6 H, 8-CH_3_, 15-CH_3_).

#### 3.1.16. General Procedures for the Synthesis of Intermediates **17–25**

Compound **16** (1 eq) was dissolved in DMF, then a diamine (5 eq) was added to this solution. The reaction was stirred at room temperature for 2 h. Water was added, and the resulting mixture was extracted with ethyl acetate. The organic layer was washed with water and brine. The organic layer was concentrated and purified through column chromatography (CH_2_Cl_2_/MeOH/NH_3_·H_2_O = 10:0.4:0.05) to obtain intermediates **17–25**.

#### 3.1.17. General Procedures for the Synthesis of Target Compounds **26T**, **28T**, **27U**–**32U**

Compounds **17–23** (1 eq), compound **3** or **4** (1.2 eq) and 1-hydroxybenzotriazole (HOBT, 1.1 eq) were dissolved in CH_2_Cl_2_, and then the solution was cooled to 0 °C and dicyclohexylcarbodiimide (DCC, 1.2 eq) was added. The ice bath was removed after 1.5 h, and the stirring was continued for another 2.5 h at rt. The precipitated DCU was removed through filtration and washed with CH_2_Cl_2_. Then, the combined filtrate was washed with water and brine and then concentrated. The resulting product was dissolved in methanol and stirred at 60 °C for 2 h. The methanol was evaporated, and the crude product was purified through column chromatography to afford the target compounds, **26T**, **28T** and **27U–32U**.

##### 3-*O*-descladinosyl-3-*O*-(2-(2-(thymin-1-yl)acetamido)ethyl)carbamoyl azithromycin-11,12-cyclic carbonate (**26T**)

Compound **17** (0.277 g, 0.37 mmol), compound **3** (0.103 g, 0.45 mmol), DCC (0.092 g, 0.45 mmol) and HOBT (0.055 g, 0.40 mmol) were dissolved in DCM according to the general procedure. The crude product was purified through column chromatography (CH_2_Cl_2_/MeOH/NH_3_·H_2_O = 10:0.5:0.05) to afford compound **26T** (80.2 mg, 0.09 mmol, 24.3%). Melting point: 145.3–146.8 °C. HRMS (ESI) (M + H)^+^ *m*/*z* 869.4871, calcd for C_41_H_69_N_6_O_14_^+^ 869.4866. ^1^H NMR (CDCl_3_, 400 MHz) δ: 7.46 (s, 1 H, -NH-thymine), 7.05 (s, 1 H, H-thymine), 6.30 (s, 1 H, -OH), 5.87 (s, 1 H, -*O*-CONH-), 5.12 (d, *J* = 8.8 Hz, 1 H, H-3), 5.06 (dd, *J* = 10.0, 2.4 Hz, 1 H, H-13), 4.65 (s, 1 H, H-11), 4.29 (s, 2 H, -C**H_2_**-T), 4.18 (d, *J* = 7.2 Hz, 1 H, H-1′), 3.57 (s, 1 H, H-5), 3.46–3.31 (m, 6 H, H-5′, -CONHC**H_2_**C**H_2_**-, H-2′), 2.88–2.82 (m, 2 H, H-10, H-2), 2.71–2.69 (m, 1 H, H-3′), 2.40–2.37 (m, 7 H, -N(CH_3_)_2_, H-9a), 2.25 (s, 3 H, N-CH_3_), 2.18–2.16 (m, 1 H, H-4), 2.07–2.01 (m, 1 H, H-9b), 1.92 (s, 4 H, thymine-CH_3_, H-8), 1.86–1.82 (m, 1 H, H-14eq), 1.73–1.70 (m, 1 H, H-4′a), 1.62–1.54 (m, 2 H, H-14ax, H-7a), 1.43 (s, 3 H, 12-CH_3_), 1.39–1.36 (m, 2 H, H-7b, H-4′b), 1.29 (s, 3 H, 6-CH_3_), 1.23 (d, *J* = 6.0 Hz, 3 H, 5′-CH_3_), 1.17 (d, *J* = 6.8 Hz, 3 H, 2-CH_3_), 1.08–1.05 (m, 6 H, 10-CH_3_, 4-CH_3_), 0.94–0.89 (m, 6 H, 8-CH_3_, 15- CH_3_). ^13^C NMR (CDCl_3_, 100 MHz) δ: 174.4, 167.4, 164.5, 157.3, 153.4, 151.8, 140.9, 111.1, 104.2, 88.9, 86.0, 85.0, 79.1, 76.1, 73.2, 71.0, 69.1, 67.7, 65.3, 61.6, 51.4, 43.3, 42.2, 40.9, 39.9, 36.6, 35.0, 29.0, 26.2, 25.6, 21.9, 21.4, 21.1, 15.1, 13.9, 12.4, 10.3, 9.6, 5.3.

##### 3-*O*-descladinosyl-3-*O*-(4-(2-(thymin-1-yl)acetamido)butyl)carbamoyl azithromycin-11,12-cyclic carbonate (**28T**)

Compound **19** (0.321 g, 0.36 mmol), compound **3** (0.080 g, 0.43 mmol), DCC (0.089 g, 0.43 mmol) and HOBT (0.054 g, 0.39 mmol) were dissolved in DCM according to the general procedure. The crude product was purified through column chromatography (CH_2_Cl_2_/MeOH/NH_3_·H_2_O = 10:0.5:0.05) to afford compound **28T** (100.5 mg, 0.11 mmol, 30.6%). Melting point: 139.5–141.2 °C. HRMS (ESI) (M + H)^+^ *m*/*z* 897.5169, calcd for C_43_H_73_N_6_O_14_^+^ 897.5179. ^1^H NMR (CDCl_3_, 400 MHz) δ: 7.10 (s, 1 H, H-thymine), 7.00 (s, 1 H, -NH-thymine), 6.15 (s, 1 H, -OH), 5.65 (s, 1 H, -*O*-CONH-), 5.12 (d, *J* = 10.0 Hz, 1 H, H-3), 5.06 (dd, *J* = 10.4, 2.4 Hz, 1 H, H-13), 4.61 (s, 1 H, H-11), 4.31 (s, 2 H, -C**H_2_**-T), 4.13 (d, *J* = 7.2 Hz, 1 H, H-1′), 3.52 (s, 1 H, H-5), 3.44–3.09 (m, 6 H, H-5′, -CONH-C**H_2_**CH_2_CH_2_C**H_2_**-, H-2′), 2.88–2.83 (m, 2 H, H-10, H-2), 2.71–2.69 (m, 1 H, H-3′), 2.41–2.36 (m, 7 H, -N(CH_3_)_2_, H-9a), 2.25 (s, 3 H, N-CH_3_), 2.17−2.15 (m, 1 H, H-4), 2.06–1.99 (m, 1 H, H-9b), 1.92–1.82 (m, 5 H, thymine-CH_3_, H-8, H-14eq), 1.78–1.75 (m, 1 H, H-4′a), 1.56–1.43 (m, 6 H, H-14ax, H-7a, -CONH-CH_2_C**H_2_**C**H_2_**-CH_2_-), 1.43 (s, 3 H, 12-CH_3_), 1.34–1.28 (m, 2 H, H-7b, H-4′b), 1.24–1.19 (m, 6 H, 6-CH_3_, 5′-CH_3_), 1.18 (d, *J* = 6.8 Hz, 3 H, 2-CH_3_), 1.08–1.05 (m, 6 H, 10-CH_3_, 4-CH_3_), 0.93−0.88 (m, 6 H, 8-CH_3_, 15- CH_3_). ^13^C NMR (CDCl_3_, 100 MHz) δ: 174.4, 166.8, 156.7, 153.4, 152.1, 141.0, 111.2, 103.7, 88.5, 86.3, 84.9, 78.9, 75.9, 73.0, 71.0, 69.0, 68.0, 65.5, 61.9, 51.6, 43.3, 42.0, 40.4, 39.8, 39.2, 36.0, 35.0, 29.7, 27.1, 26.1, 25.8, 21.8, 21.4, 21.1, 15.6, 13.6, 12.4, 10.2, 9.5, 5.0.

##### 3-*O*-descladinosyl-3-*O*-(3-(2-(uracil-1-yl)acetamido)propyl)carbamoyl azithromycin-11, 12-cyclic carbonate (**27U**)

Compound **18** (0.355 g, 0.50 mmol), compound **4** (0.100 g, 0.60 mmol), DCC (0.123 g, 0.60 mmol) and HOBT (0.074 g, 0.55 mmol) were dissolved in DCM according to the general procedure. The crude product was purified through column chromatography (CH_2_Cl_2_/MeOH/NH_3_·H_2_O = 10:0.5:0.05) to afford compound **27U** (80.5 g, 0.09 mmol, 18.0%). Melting point: 139.6–140.6 °C. HRMS (ESI) (M + H)^+^ *m*/*z* 869.4864, calcd for C_41_H_69_N_6_O_14_^+^ 869.4866. ^1^H NMR (CDCl_3_, 400 MHz) δ: 7.31 (s, 1 H, -NH-uracil), 7.25 (d, *J* = 7.6 Hz, 1 H, H-uracil), 6.15 (s, 1 H, -OH), 5.89 (s, 1 H, -*O*-CONH-), 5.71 (d, *J* = 7.6 Hz, 1 H, H-uracil), 5.11 (d, *J* = 10.4 Hz, 1 H, H-3), 5.04 (dd, *J* = 10.4, 2.4 Hz, 1 H, H-13), 4.58 (s, 1 H, H-11), 4.48–4.27 (m, 2 H, -C**H_2_**-U), 4.11 (d, *J* = 7.6 Hz, 1 H, H-1′), 3.51 (s, 1 H, H-5), 3.48–3.09 (m, 6 H, H-5′, -CONH-C**H_2_**CH_2_C**H_2_**-, H-2′), 2.89–2.81 (m, 2 H, H-10, H-2), 2.73–2.67 (m, 1 H, H-3′), 2.37–2.36 (m, 7 H, -N(CH_3_)_2_, H-9a), 2.25 (s, 3 H, N-CH_3_), 2.16–2.14 (m, 1 H, H-4), 2.05−2.01 (m, 1 H, H-9b), 1.89−1.82 (m, 2 H, H-8, H-14eq), 1.74−1.70 (m, 1 H, H-4′a), 1.69–1.54 (m, 4 H, H-14ax, H-7a, -CONHCH_2_C**H_2_**CH_2_-), 1.43 (s, 3 H, 12-CH_3_), 1.34–1.31 (m, 2 H, H-7b, H-4′b), 1.24–1.21 (m, 6 H, 6-CH_3_, 2-CH_3_), 1.16 (d, *J* = 7.2 Hz, 3 H, 5′-CH_3_), 1.08–1.04 (m, 6 H, 10-CH_3_, 4-CH_3_), 0.93–0.88 (m, 6 H, 8-CH_3_, 15- CH_3_). ^13^C NMR (CDCl_3_, 100 MHz) δ: 174.6, 167.0, 164.5, 157.0, 153.4, 151.9, 145.2, 103.8, 102.6, 88.2, 86.2, 85.0, 78.8, 75.9, 73.1, 71.0, 69.1, 67.9, 64.9, 61.8, 51.5, 43.3, 42.0, 39.7, 37.7, 36.5, 36.1, 35.0, 29.5, 28.9, 26.0, 25.8, 21.7, 21.4, 21.1, 15.5, 13.6, 10.2, 9.6, 5.1.

##### 3-*O*-descladinosyl-3-*O*-(4-(2-(uracil-1-yl)acetamido)butyl)carbamoyl azithromycin-11,12-cyclic carbonate (**28U**)

Compound **19** (0.250 g, 0.34 mmol), compound **4** (0.070 g, 0.41 mmol), DCC (0.084 g, 0.41 mmol) and HOBT (0.051 g, 0.37 mmol) were dissolved in DCM according to the general procedure. The crude product was purified through column chromatography (CH_2_Cl_2_/MeOH/NH_3_·H_2_O = 10:0.5:0.05) to afford compound **28U** (52.7 mg, 0.06 mmol, 17.5%). Melting point: 136.9–138.3 °C. HRMS (ESI) (M + H)^+^ *m*/*z* 883.5014, calcd for C_42_H_71_N_6_O_14_^+^ 883.5023. ^1^H NMR (CDCl_3_, 400 MHz) δ: 7.30 (d, *J* = 8.0 Hz, 1 H, H-uracil), 7.17 (s, 1 H, -NH-uracil), 6.23 (s, 1 H, -OH), 5.72 (d, *J* = 8.0 Hz, 1 H, H-uracil), 5.68 (s, 1 H, -*O*-CONH-), 5.11 (d, *J* = 10.4 Hz, 1 H, H-3), 5.06 (dd, *J* = 10.4, 2.4 Hz, 1 H, H-13), 4.59 (s, 1 H, H-11), 4.38 (s, 2 H, -C**H_2_**-U), 4.12 (d, *J* = 7.2 Hz, 1 H, H-1′), 3.51 (s, 1 H, H-5), 3.40–3.04 (m, 6 H, H-5′, -CONH-C**H_2_**CH_2_CH_2_C**H_2_**-, H-2′), 2.90–2.84 (m, 2 H, H-10, H-2), 2.64–2.58 (m, 1 H, H-3′), 2.39–2.31 (m, 7 H, -N(CH_3_)_2_, H-9a), 2.25 (s, 3 H, N-CH_3_), 2.17–2.14 (m, 1 H, H-4), 2.06–2.00 (m, 1 H, H-9b), 1.89–1.82 (m, 2 H, H-8, H-14eq), 1.72–1.69 (m, 1 H, H-4′a), 1.60–1.57 (m, 6 H, H-14ax, H-7a, -CONHCH_2_C**H_2_**C**H_2_**CH_2_-), 1.43 (s, 3 H, 12-CH_3_), 1.33−1.31 (m, 2 H, H-7b, H-4′b), 1.23–1.17 (m, 9 H, 6-CH_3_, 2-CH_3_, 5′-CH_3_), 1.08–1.05 (m, 6 H, 10-CH_3_, 4-CH_3_), 0.93–0.88 (m, 6 H, 8-CH_3_, 15- CH_3_). ^13^C NMR (CDCl_3_, 100 MHz) δ: 174.6, 166.7, 164.5, 156.8, 153.4, 152.0, 145.3, 103.7, 102.6, 88.1, 86.3, 84.9, 78.8, 75.9, 73.1, 71.0, 69.1, 68.0, 65.5, 61.8, 51.5, 43.3, 42.0, 40.4, 39.2, 36.0, 35.0, 29.3, 27.2, 26.1, 25.8, 21.7, 21.4, 21.1, 15.6, 13.6, 10.2, 9.5, 5.0.

##### 3-*O*-descladinosyl-3-*O*-(5-(2-(uracil-1-yl)acetamido)pentyl)carbamoyl azithromycin-11,12-cyclic carbonate (**29U**)

Compound **20** (0.285 g, 0.38 mmol), compound **4** (0.078 g, 0.45 mmol), DCC (0.094 g, 0.45 mmol) and HOBT (0.057 g, 0.42 mmol) were dissolved in DCM according to the general procedure. The crude product was purified through column chromatography (CH_2_Cl_2_/MeOH/NH_3_·H_2_O = 10:0.45:0.05) to afford compound **29U** (60.5 mg, 0.07 mmol, 18.4%). Melting point: 137.8–139.9 °C. HRMS (ESI) (M + H)^+^ *m*/*z* 897.5181, calcd for C_43_H_73_N_6_O_14_^+^ 897.5179. ^1^H NMR (CDCl_3_, 400 MHz) δ: 7.32 (d, *J* = 8.0 Hz, 1 H, H-uracil), 7.00 (s, 1 H, -NH-uracil), 6.17 (s, 1 H, -OH), 5.71 (d, *J* = 8.0 Hz, 1 H, H-uracil, -*O*-CONH-), 5.12 (d, *J* = 10.4 Hz, 1 H, H-3), 5.05 (dd, *J* = 10.8, 2.4 Hz, 1 H, H-13), 4.58 (s, 1 H, H-11), 4.43 (s, 2 H, -C**H_2_**-U), 4.13 (d, *J* = 7.2 Hz, 1 H, H-1′), 3.51 (s, 1 H, H-5), 3.42–3.19 (m, 6 H, H-5′, -CONH-C**H_2_**(CH_2_)_3_C**H_2_**-, H-2′), 2.90–2.80 (m, 2 H, H-10, H-2), 2.73–2.67 (m, 1 H, H-3′), 2.40–2.35 (m, 7 H, -N(CH_3_)_2_, H-9a), 2.25 (s, 3 H, N-CH_3_), 2.16–2.14 (m, 1 H, H-4), 2.04–1.99 (m, 1 H, H-9b), 1.88–1.84 (m, 2 H, H-8, H-14eq), 1.76–1.72 (m, 1 H, H-4′a), 1.61–1.50 (m, 6 H, H-14ax, H-7a, -CONHCH_2_C**H_2_**CH_2_C**H_2_**CH_2_-), 1.42 (s, 3 H, 12-CH_3_), 1.37–1.28 (m, 4 H, -CONH-CH_2_CH_2_C**H_2_**CH_2_CH_2_-, H-7b, H-4′b), 1.23–1.17 (m, 9 H, 6-CH_3_, 5′-CH_3_, 2-CH_3_), 1.08–1.05 (m, 6 H, 10-CH_3_, 4-CH_3_), 0.92–0.88 (m, 6 H, 8-CH_3_, 15- CH_3_). ^13^C NMR (CDCl_3_, 100 MHz) δ: 174.6, 166.6, 164.4, 156.8, 153.4, 151.6, 145.5, 103.6, 102.3, 88.0, 86.4, 84.8, 78.9, 76.0, 73.1, 71.0, 69.1, 68.1, 65.6, 61.9, 51.0, 43.2, 42.0, 38.7, 35.9, 35.0, 29.8, 29.5, 28.8, 26.0, 25.8, 25.3, 21.7, 21.4, 21.1, 15.7, 13.5, 9.4, 5.0.

##### 3-*O*-descladinosyl-3-*O*-(6-(2-(uracil-1-yl)acetamido)hexyl)carbamoyl azithromycin-11,12-cyclic carbonate (**30U**)

Compound **21** (0.316 g, 0.42 mmol), compound **4** (0.085 g, 0.50 mmol), DCC (0.104 g, 0.50 mmol) and HOBT (0.063 g, 0.46 mmol) were dissolved in DCM according to the general procedure. The crude product was purified through column chromatography (CH_2_Cl_2_/MeOH/NH_3_·H_2_O = 10:0.45:0.05) to afford compound **30U** (61.3 mg, 0.07 mmol, 16.0%). Melting point: 137.5–139.6 °C. HRMS (ESI) (M + H)^+^ *m*/*z* 911.5326, calcd for C_44_H_75_N_6_O_14_^+^ 911.5336. ^1^H NMR (CDCl_3_, 400 MHz) δ: 7.33 (d, *J* = 8.0 Hz, 1 H, H-uracil), 7.06 (s, 1 H, -NH-uracil), 6.19 (s, 1 H, -OH), 5.71 (d, *J* = 8.0 Hz, 1 H, H-uracil), 5.63 (s, 1 H, -*O*-CONH-), 5.11 (d, *J* = 10.4 Hz, 1 H, H-3), 5.05 (dd, *J* = 10.8, 2.4 Hz, 1 H, H-13), 4.60 (s, 1 H, H-11), 4.45 (s, 2 H, -C**H_2_**-U), 4.12 (d, *J* = 7.2 Hz, 1 H, H-1′), 3.54 (s, 1 H, H-5), 3.44–3.04 (m, 6 H, H-5′, -CONH-C**H_2_**(CH_2_)_4_C**H_2_**-, H-2′), 2.90–2.84 (m, 2 H, H-10, H-2), 2.64–2.58 (m, 1 H, H-3′), 2.39–2.31 (m, 7 H, -N(CH_3_)_2_, H-9a), 2.25 (s, 3 H, N-CH_3_), 2.17–2.14 (m, 1 H, H-4), 2.06–2.00 (m, 1 H, H-9b), 1.89–1.82 (m, 2 H, H-8, H-14eq), 1.72–1.69 (m, 1 H, H-4′a), 1.60–1.57 (m, 6 H, H-14ax, H-7a, -CONHCH_2_C**H_2_**(CH_2_)_2_C**H_2_**CH_2_-), 1.43–1.31 (m, 9 H, 12-CH_3_, H-7b, H-4′b, -CONHCH_2_CH_2_C**H_2_**C**H_2_**CH_2_CH_2_-), 1.23–1.17 (m, 9 H, 6-CH_3_, 2-CH_3_, 5′-CH_3_), 1.08–1.05 (m, 6 H, 10-CH_3_, 4-CH_3_), 0.93–0.88 (m, 6 H, 8-CH_3_, 15- CH_3_). ^13^C NMR (CDCl_3_, 100 MHz) δ: 174.6, 166.7, 164.5, 156.8, 153.4, 152.0, 145.3, 103.7, 102.6, 88.1, 86.3, 84.9, 78.8, 75.9, 73.1, 71.0, 69.1, 68.0, 65.5, 61.8, 51.5, 43.3, 42.0, 40.4, 39.2, 36.0, 35.0, 29.3, 27.2, 26.1, 25.8, 21.7, 21.4, 21.1, 15.6, 13.6, 10.2, 9.5, 5.0.

##### 3-*O*-descladinosyl-3-*O*-(7-(2-(uracil-1-yl)acetamido)heptyl)carbamoyl azithromycin-11, 12-cyclic carbonate (**31U**)

Compound **22** (0.362 g, 0.47 mmol), compound **4** (0.095 g, 0.56 mmol), DCC (0.095 g, 0.56 mmol) and HOBT (0.070 g, 0.52 mmol) were dissolved in DCM according to the general procedure. The crude product was purified through column chromatography (CH_2_Cl_2_/MeOH/NH_3_·H_2_O = 10:0.4:0.05) to afford compound **31U** (100.2 mg, 0.11 mmol, 23.0%). Melting point: 120.5–121.9 °C. HRMS (ESI) (M + H)^+^ *m*/*z* 925.5489, calcd for C_45_H_77_N_6_O_14_^+^ 925.5492. ^1^H NMR (CDCl_3_, 400 MHz) δ: 7.34 (d, *J* = 8.0 Hz, 1 H, H-uracil), 6.97 (s, 1 H, -NH-uracil), 6.08 (s, 1 H, -H), 5.71 (d, *J* = 8.0 Hz, 1 H, H-uracil), 5.58 (s, 1 H, -*O*-CONH-), 5.11 (d, *J* = 10.4 Hz, 1 H, H-3), 5.05 (dd, *J* = 10.8, 2.4 Hz, 1 H, H-13), 4.58 (s, 1 H, H-11), 4.45 (q, *J* = 16.0, 12.0 Hz, 2 H, -C**H_2_**-U), 4.13 (d, *J* = 7.2 Hz, 1 H, H-1′), 3.52 (s, 1 H, H-5), 3.43–3.03 (m, 6 H, H-5′, -CONH-C**H_2_**(CH_2_)_5_C**H_2_**-, H-2′), 2.90–2.81 (m, 2 H, H-10, H-2), 2.64–2.58 (m, 1 H, H-3′), 2.37–2.31 (m, 7 H, -N(CH_3_)_2_, H-9a), 2.24 (s, 3 H, N-CH_3_), 2.17–2.11 (m, 1 H, H-4), 2.05–1.99 (m, 1 H, H-9b), 1.88–1.80 (m, 2 H, H-8, H-14eq), 1.71–1.67 (m, 1 H, H-4′a), 1.61–1.50 (m, 6 H, H-14ax, H-7a, -CONHCH_2_C**H_2_**(CH_2_)_3_C**H_2_**CH_2_-), 1.42 (s, 3 H, 12-CH_3_), 1.32-1.26 (m, 8 H, H-7b, H-4′b, -CONHCH_2_CH_2_C**H_2_**C**H_2_**C**H_2_**CH_2_CH_2_-), 1.22–1.18 (m, 9 H, 6-CH_3_, 2-CH_3_, 5′-CH_3_), 1.07–1.06 (m, 6 H, 10-CH_3_, 4-CH_3_), 0.93–0.90 (m, 6 H, 8-CH_3_, 15-CH_3_). ^13^C NMR (CDCl_3_, 100 MHz) δ: 174.7, 166.7, 164.8, 156.6, 153.4, 151.6, 145.6, 103.6, 102.2, 87.8, 86.4, 84.8, 78.8, 75.9, 73.1, 70.9, 69.2, 68.1, 65.6, 61.9, 51.0, 43.3, 41.9, 40.6, 39.9, 39.3, 36.0, 35.0, 29.6, 29.0, 28.9, 28.1, 26.2, 26.0, 25.8, 21.7, 21.4, 21.2, 15.7, 13.5, 10.1, 9.4, 5.0.

##### 3-*O*-descladinosyl-3-*O*-(8-(2-(uracil-1-yl)acetamido)octyl)carbamoyl azithromycin-11,12-cyclic carbonate (**32U**)

Compound **23** (0.366 g, 0.47 mmol), compound **4** (0.095 g, 0.56 mmol), DCC (0.095 g, 0.56 mmol) and HOBT (0.070 g, 0.52 mmol) were dissolved in DCM according to the general procedure. The crude product was purified through column chromatography (CH_2_Cl_2_/MeOH/NH_3_·H_2_O = 10:0.4:0.05) to afford compound **32U** (83.5 mg, 0.09 mmol, 18.9%). Melting point: 121.9–123.6 °C. HRMS (ESI) (M + H)^+^ *m*/*z* 939,5642, calcd for C_46_H_79_N_6_O_14_^+^ 939.5649. ^1^H NMR (CDCl_3_, 400 MHz) δ: 7.34 (d, *J* = 8.0 Hz, 1 H, H-uracil), 6.87 (s, 1 H, -NH-uracil), 6.11 (s, 1 H, -OH), 5.71 (d, *J* = 8.0 Hz, 1 H, H-uracil), 5.47 (s, 1 H, -*O*-CONH-), 5.11 (d, *J* = 10.4 Hz, 1 H, H-3), 5.06 (dd, *J* = 10.8, 2.4 Hz, 1 H, H-13), 4.60 (s, 1 H, H-11), 4.41 (q, *J* = 22.4, 15.6 Hz, 2 H, -C**H_2_**-U), 4.12 (d, *J* = 7.2 Hz, 1 H, H-1′), 3.54 (s, 1 H, H-5), 3.43–3.03 (m, 6 H, H-5′, -CONH-C**H_2_**(CH_2_)_6_C**H_2_**-, H-2′), 2.90–2.81 (m, 2 H, H-10, H-2), 2.64–2.58 (m, 1 H, H-3′), 2.39–2.37 (m, 7 H, -N(CH_3_)_2_, H-9a), 2.24 (s, 3 H, N-CH_3_), 2.16-2.14 (m, 1 H, H-4), 2.05–1.99 (m, 1 H, H-9b), 1.89–1.82 (m, 2 H, H-8, H-14eq), 1.69–1.66 (m, 1 H, H-4′a), 1.62–1.48 (m, 6 H, H-14ax, H-7a, -CONHCH_2_C**H_2_**(CH_2_)_4_C**H_2_**CH_2_-), 1.42 (s, 3 H, 12-CH_3_), 1.29–1.25 (m, 10 H, H-7b, H-4′b, -CONHCH_2_CH_2_C**H_2_**C**H_2_**C**H_2_**C**H_2_**CH_2_CH_2_-), 1.22–1.17 (m, 9 H, 6-CH_3_, 2-CH_3_, 5′-CH_3_), 1.08-1.06 (m, 6 H, 10-CH_3_, 4-CH_3_), 0.92–0.88 (m, 6 H, 8-CH_3_, 15-CH_3_). ^13^C NMR (CDCl_3_, 100 MHz) δ: 174.6, 166.6, 164.6, 156.5, 153.4, 151.5, 145.5, 103.6, 102.2, 87.9, 86.4, 84.9, 78.8, 77.3, 75.9, 73.1, 70.9, 69.2, 68.1, 65.6, 61.9, 50.8, 43.3, 42.0, 40.9, 40.0, 39.6, 36.0, 35.0, 29.9, 29.1, 29.0, 28.7, 26.4, 26.3, 26.0, 25.8, 21.7, 21.4, 21.2, 15.6, 13.5, 10.1, 9.4, 5.0.

#### 3.1.18. General Procedures for the Synthesis of Target Compounds **29A–30A**, **32A–34A**

Compound **20**–**21** or **23**–**25** (1 eq), compound **8** (1.2 eq) and HOBT (1.1 eq) were dissolved in CH_2_Cl_2_, then the solution was cooled to 0 °C and DCC (1.2 eq) was added. The ice bath was removed after 1.5 h, and the stirring was continued for another 2.5 h at rt. The precipitated DCU was removed through filtration and washed with DCM. Then, the combined filtrate was washed with water and brine and concentrated. The resulting mixture was dissolved in methanol and stirred at 60 °C for 2 h. The methanol was concentrated, and the crude product was purified through column chromatography (CH_2_Cl_2_/MeOH/NH_3_·H_2_O = 10:0.5:0.05). The resulting compound was dissolved in EtOH/HCl (8:2 mL), and the solution was stirred for 30 min. Then, the pH was adjusted to 10 with NH_3_·H_2_O, and then CH_2_Cl_2_ was added for extraction. The organic phase was washed with water and brine once, and the crude product was purified through column chromatography to afford compounds **29A**–**30A** and **32A**–**34A**.

##### 3-*O*-descladinosyl-3-*O*-(5-(2-(adenin-9-yl)acetamido)pentyl)carbamoyl azithromycin-11, 12-cyclic carbonate (**29A**)

Compound **20** (0.425 g, 0.57 mmol), compound **8** (0.267 g, 0.68 mmol), DCC (0.115 g, 0.68 mmol) and HOBT (0.085 g, 0.63 mmol) were dissolved in DCM according to the general procedure. The crude product was purified through column chromatography (CH_2_Cl_2_/MeOH/NH_3_·H_2_O = 10:0.25:0.05) to afford compound **29A** (95.8 mg, 0.11 mmol, 55.0%). Melting point: 127.4–130.4 °C. HRMS (ESI) (M + H)^+^ *m*/*z* 920.5424, calcd for C_44_H_74_N_9_O_12_^+^ 920.5451. ^1^H NMR (CDCl_3_, 400 MHz) δ: 8.34 (s, 1 H, H-adenine), 7.96 (s, 1 H, H-adenine), 6.16 (s, 1 H, -OH), 5.83 (s, 1 H, -NH_2_-), 5.45 (s, 1 H, -*O*-CONH-), 5.14 (d, *J* = 10.0 Hz, 1 H, H-3), 5.07–4.87 (m, 1 H, H-13, -C**H_2_**-A), 4.58 (s, 1 H, H-11), 4.15 (d, *J* = 7.2 Hz, 1 H, H-1′), 3.56 (s, 1 H, H-5), 3.45–3.06 (m, 6 H, H-5′, -CONH-C**H_2_**(CH_2_)_3_C**H_2_**-, H-2′), 2.88–2.81 (m, 2 H, H-10, H-2), 2.73–2.68 (m, 1 H, H-3′), 2.45–2.34 (m, 7 H, -N(CH_3_)_2_, H-9a), 2.23 (s, 3 H, N-CH_3_), 2.18–2.16 (m, 1 H, H-4), 2.04–1.99 (m, 1 H, H-9b), 1.90–1.83 (m, 2 H, H-8, H-14eq), 1.77–1.73 (m, 1 H, H-4′a), 1.61–1.48 (m, 6 H, H-14ax, H-7a, -CONHCH_2_C**H_2_**CH_2_C**H_2_**CH_2_-), 1.41 (s, 3 H, 12-CH_3_), 1.37–1.28 (m, 4 H, H-7b, H-4′b, -CONHCH_2_CH_2_C**H_2_**CH_2_CH_2_-), 1.25–1.18 (m, 9 H, 6-CH_3_, 2-CH_3_, 5′-CH_3_), 1.07–1.04 (m, 6 H, 10-CH_3_, 4-CH_3_), 0.93–0.87 (m, 6 H, 8-CH_3_, 15-CH_3_). ^13^C NMR (CDCl_3_, 100 MHz) δ: 174.4, 156.6, 155.6, 153.1, 141.5, 119.4, 103.7, 86.3, 84.9, 78.9, 75.9, 73.0, 70.7, 69.0, 65.8, 61.8, 46.7, 43.4, 40.3, 39.6, 36.0, 29.5, 28.4, 26.1, 25.6, 23.6, 21.7, 21.4, 21.1, 15.6, 13.6, 10.1, 5.1.

##### 3-*O*-descladinosyl-3-*O*-(6-(2-(adenin-9-yl)acetamido)hexyl)carbamoyl azithromycin-11, 12-cyclic carbonate (**30A**)

Compound **21** (0.250 g, 0.33 mmol), compound **8** (0.157 g, 0.40 mmol), DCC (0.067 g, 0.40 mmol) and HOBT (0.049 g, 0.36 mmol) were dissolved in DCM according to the general procedure. The crude product was purified through column chromatography (CH_2_Cl_2_/MeOH/NH_3_·H_2_O = 10:0.3:0.05) to afford compound **30A** (58.5 mg, 0.06 mmol, 54.6%). Melting point: 128.1–129.9 °C. HRMS (ESI) (M + H)^+^ *m*/*z* 934.5600, calcd for C_45_H_76_N_9_O_12_^+^ 934.5608. ^1^H NMR (CDCl_3_, 400 MHz) δ: 8.33 (s, 1 H, H-adenine), 7.95 (s, 1 H, H-adenine), 7.03 (t, *J* = 5.6 Hz, 1 H, -CON**H**-CH_2_-A), 6.13–6.07 (m, 3 H, -NH_2_-, -OH), 5.28 (s, 1 H, -*O*-CONH-), 5.15 (d, *J* = 10.0 Hz, 1 H, H-3), 5.06 (dd, *J* = 10.8, 2.0 Hz, 1 H, H-13), 4.90 (q, 2 H, -C**H_2_**-A), 4.60 (s, 1 H, H-11), 4.12 (d, *J* = 7.2 Hz, 1 H, H-1′), 3.56 (s, 1 H, H-5), 3.42–3.01 (m, 6 H, H-5′, -CONH-C**H_2_**(CH_2_)_4_C**H_2_**-, H-2′), 2.89–2.82 (m, 2 H, H-10, H-2), 2.49–2.43 (m, 1 H, H-3′), 2.38–2.30 (m, 7 H, -N(CH_3_)_2_, H-9a), 2.24 (s, 3 H, N-CH_3_), 2.18–2.16 (m, 1 H, H-4), 2.04–1.99 (m, 1 H, H-9b), 1.89–1.82 (m, 2 H, H-8, H-14eq), 1.68–1.55 (m, 3 H, H-4′a, H-14ax, H-7a), 1.46–1.42 (m, 7 H, -CONHCH_2_C**H_2_**(CH_2_)_2_C**H_2_**CH_2_-, 12-CH_3_), 1.27–1.18 (m, 13 H, H-7b, H-4′b, -CONHCH_2_CH_2_C**H_2_**C**H_2_**CH_2_CH_2_-, 6-CH_3_, 2-CH_3_, 5′-CH_3_), 1.08–1.06 (m, 6 H, 10-CH_3_, 4-CH_3_), 0.92-0.90 (m, 6 H, 8-CH_3_, 15-CH_3_). ^13^C NMR (CDCl_3_, 100 MHz) δ: 174.5, 166.2, 156.6, 155.7, 153.4, 153.1, 150.0, 141.3, 119.3, 103.8, 88.0, 86.3, 84.9, 78.9, 77.3, 75.9, 73.0, 70.8, 69.4, 68.0, 61.8, 47.0, 43.3, 42.1, 40.4, 39.2, 36.0, 34.9, 30.0, 28.9, 28.8, 26.1, 25.8, 25.7, 25.6, 21.7, 21.4, 21.2, 15.6, 10.1, 5.1.

##### 3-*O*-descladinosyl-3-*O*-(8-(2-(adenin-9-yl)acetamido)octyl)carbamoyl azithromycin-11, 12-cyclic carbonate (**32A**)

Compound **23** (0.315 g, 0.40 mmol), compound **8** (0.188 g, 0.48 mmol), DCC (0.081 g, 0.48 mmol) and HOBT (0.059 g, 0.44 mmol) were dissolved in DCM according to the general procedure. The crude product was purified through column chromatography (CH_2_Cl_2_/MeOH/NH_3_·H_2_O = 10:0.3:0.05) to afford compound **32A** (97.8 mg, 0.10 mmol, 76.9%). Melting point: 125.7–127.2 °C. HRMS (ESI) (M + H)^+^ *m*/*z* 962.5928, calcd for C_47_H_80_N_9_O_12_^+^ 962.5921. ^1^H NMR (MeOD-*d_4_*, 400 MHz) δ: 8.19 (s, 1 H, H-adenine), 8.10 (s, 1 H, H-adenine), 5.11–5.04 (m, 2 H, H-3, H-13), 4.93 (q, 2 H, -C**H_2_**-A), 4.60 (s, 1 H, H-11), 4.20 (d, *J* = 7.2 Hz, 1 H, H-1′), 3.66–3.19 (m, 6 H, H-5′, -CONH-C**H**_2_(CH_2_)_6_C**H_2_**-, H-2′, H-5), 3.04–2.95 (m, 3 H, -CONHC**H**_2_-, H-10, H-2), 2.60–2.55 (m, 1 H, H-3′), 2.40–2.33 (m, 7 H, -N(CH_3_)_2_, H-9a), 2.23 (s, 3 H, N-CH_3_), 2.20–2.13 (m, 2 H, H-4, H-9b), 1.84–1.78 (m, 2 H, H-8, H-14eq), 1.72–1.63 (m, 3 H, H-4′a, H-14ax, H-7a), 1.52–1.50 (m, 4 H, -CONHCH_2_C**H_2_**(CH_2_)_4_C**H_2_**CH_2_-), 1.46 (s, 3 H, 12-CH_3_), 1.42–1.25 (m, 10 H, -CONHCH_2_CH_2_C**H_2_**C**H_2_**C**H_2_**C**H_2_**CH_2_CH_2_-, H-7b, H-4′b), 1.25–1.16 (m, 9 H, 6-CH_3_, 2-CH_3_, 5′-CH_3_), 1.09–1.06 (m, 6 H, 10-CH_3_, 4-CH_3_), 0.94–0.89 (m, 6 H, 8-CH_3_, 15-CH_3_). ^13^C NMR (MeOD-*d_4_*, 100 MHz) δ: 172.6, 164.7, 154.9, 153.5, 151.1, 150.1, 147.3, 139.8, 116.0, 99.1, 84.1, 82.7, 81.1, 76.0, 73.5, 71.1, 68.7, 66.3, 64.9, 62.1, 59.0, 42.8, 40.8, 38.6, 38.2, 37.2, 36.9, 33.5, 31.5, 28.5, 27.2, 26.6, 26.5, 24.1, 23.4, 22.5, 18.9, 17.9, 17.7, 12.3, 9.8, 6.8, 5.9.

##### 3-*O*-descladinosyl-3-*O*-(10-(2-(adenin-9-yl)acetamido)decyl)carbamoyl azithromycin-11, 12-cyclic carbonate (**33A**)

Compound **24** (0.302 g, 0.37 mmol), compound **8** (0.175 g, 0.44 mmol), DCC (0.092 g, 0.44 mmol) and HOBT (0.055 g, 0.41 mmol) were dissolved in DCM according to the general procedure. The crude product was purified through column chromatography (CH_2_Cl_2_/MeOH/NH_3_·H_2_O = 10:0.2:0.05) to afford compound **33A** (100.8 mg, 0.10 mmol, 59.8%). Melting point: 127.5–128.9 °C. HRMS (ESI) (M + H)^+^ *m*/*z* 990.6198, calcd for C_49_H_84_N_9_O_12_^+^ 990.6234. ^1^H NMR (MeOD-*d_4_*, 400 MHz) δ: 8.19 (s, 1 H, H-adenine), 8.10 (s, 1 H, H-adenine), 5.10–5.04 (m, 2 H, H-3, H-13), 4.93 (s, 2 H, -C**H_2_**-A), 4.59 (s, 1 H, H-11), 4.20 (d, *J* = 7.2 Hz, 1 H, H-1′), 3.66 (s, 2 H, H-5), 3.44–3.40 (m, 1 H, H-5′), 3.27–3.19 (m, 4 H, H-2′, -CONH-C**H**_2_(CH_2_)_8_C**H_2_**-), 3.05–2.91 (m, 3 H, H-2, H-10, -CONH-C**H**_2_(CH_2_)_9_-), 2.62–2.56 (m, 1 H, H-3′), 2.40–2.33 (m, 7 H, -N(CH_3_)_2_, H-9a), 2.23 (s, 3 H, N-CH_3_), 2.20–2.12 (m, 2 H, H-4, H-9b), 1.84-1.78 (m, 2 H, H-8, H-14eq), 1.72–1.63 (m, 3 H, H-4′a, H-14ax, H-7a), 1.51–1.50 (m, 4 H, -CONHCH_2_C**H_2_**(CH_2_)_6_C**H_2_**CH_2_-), 1.46 (s, 3 H, 12-CH_3_), 1.37–1.31 (m, 14 H, H-7b, H-4′b, -CONHCH_2_CH_2_C**H_2_**C**H_2_**C**H_2_**C**H_2_**C**H_2_**C**H_2_**CH_2_CH_2_-), 1.25–1.16 (m, 9 H, 6-CH_3_, 2-CH_3_, 5′-CH_3_), 1.09–1.06 (m, 6 H, 10-CH_3_, 4-CH_3_), 0.94–0.89 (m, 6 H, 8-CH_3_, 15-CH_3_). ^13^C NMR (MeOD-*d_4_*, 176 MHz) δ: 174.9, 167.0, 157.3, 155.9, 153.5, 152.4, 149.6, 142.2, 118.3, 101.5, 86.5, 85.0, 83.4, 78.3, 75.9, 73.4, 71.0, 68.6, 67.2, 64.4, 61.4, 45.1, 43.1, 40.9, 40.5, 39.6, 39.3, 35.9, 33.8, 30.8, 29.6, 29.2, 29.0, 28.9,26.5, 25.8, 24.8, 21.3, 20.3, 20.1, 14.7, 12.1, 9.2, 8.2, 3.8.

##### 3-*O*-descladinosyl-3-*O*-(12-(2-(adenin-9-yl)acetamido)dodecyl)carbamoyl azithromycin-11, 12-cyclic carbonate (**34A**)

Compound **25** (0.250 g, 0.30 mmol), compound **8** (0.141 g, 0.36 mmol), DCC (0.074 g, 0.36 mmol) and HOBT (0.045 g, 0.33 mmol) were dissolved in DCM according to the general procedure. The crude product was purified through column chromatography (CH_2_Cl_2_/MeOH/NH_3_·H_2_O=10:0.2:0.05) to afford compound **34A** (86.4 mg, 0.08 mmol, 56.7%). Melting point: 126.9–127.7 °C. HRMS (ESI) (M + H)^+^ *m*/*z* 1018.6531, calcd for C_51_H_88_N_9_O_12_^+^ 1018.6547. ^1^H NMR (MeOD-*d_4_*, 400 MHz) δ: 8.19 (s, 1 H, H-adenine), 8.10 (s, 1 H, H-adenine), 5.10–5.04 (m, 2 H, H-3, H-13), 4.93 (s, 2 H, -C**H_2_**-A), 4.59 (s, 1 H, H-11), 4.20 (d, *J* = 7.2 Hz, 1 H, H-1′), 3.66 (s, 2 H, H-5), 3.44–3.40 (m, 1 H, H-5′), 3.28–3.19 (m, 4 H, H-2′, -CONH-C**H**_2_(CH_2_)_10_C**H_2_**-), 3.05–2.91 (m, 3 H, H-2, H-10, -CONH-C**H**_2_(CH_2_)_11_-), 2.62–2.56 (m, 1 H, H-3′), 2.40–2.36 (m, 7 H, -N(CH_3_)_2_, H-9a), 2.23 (s, 3 H, N-CH_3_), 2.20–2.12 (m, 2 H, H-4, H-9b), 1.87–1.78 (m, 2 H, H-8, H-14eq), 1.72–1.63 (m, 3 H, H-4′a, H-14ax, H-7a), 1.53-1.50 (m, 4 H, -CONHCH_2_C**H_2_**(CH_2_)_8_C**H_2_**CH_2_-), 1.46 (s, 3 H, 12-CH_3_), 1.37–1.26 (m, 18 H, H-7b, H-4′b, -CONHCH_2_CH_2_C**H_2_**C**H_2_**C**H_2_**C**H_2_**C**H_2_**C**H_2_**C**H_2_**C**H_2_**CH_2_CH_2_-), 1.20–1.16 (m, 9 H, 6-CH_3_, 2-CH_3_, 5′-CH_3_), 1.09–1.06 (m, 6 H, 10-CH_3_, 4-CH_3_), 0.94-0.89 (m, 6 H, 8-CH_3_, 15-CH_3_). ^13^C NMR (MeOD-*d_4_*, 176 MHz) δ: 174.9, 167.0, 157.3, 155.9, 153.5, 152.4, 149.6, 142.2, 118.3, 101.5, 86.5, 85.0, 83.4, 78.3, 75.9, 73.4, 71.0, 68.6, 67.2, 64.4, 61.4, 45.1, 43.1, 40.9, 40.5, 39.6, 39.3, 35.9, 33.8, 30.8, 29.6, 29.3, 29.0, 28.9, 26.5, 25.7, 24.8, 21.3, 20.3, 20.1,14.7, 12.1, 9.2, 8.3, 3.8.

#### 3.1.19. 3-*O*-descladinosyl-3-*O*-(8-(2-(adenin-9-yl)acetamido)octyl)carbamoyl azithromycin (**35A**)

Compound **32A** (0.164 g, 0.17 mmol) was dissolved in THF/H_2_O (1:1), and LiOH·H_2_O (0.036 g, 0.85 mmol) was added to the solution. The mixture was stirred for 5 h at room temperature and then extracted with a mixture of water and CH_2_Cl_2_. The organic phase was concentrated and purified through column chromatography (CH_2_Cl_2_/MeOH/NH_3_·H_2_O = 10:0.3:0.05) to afford compound **35A** (87.8 mg, 0.09 mmol, 55.2%). Melting point: 125.7–127.2 °C. HRMS (ESI) (M + H)^+^ *m*/*z* 936.6102, calcd for C_46_H_82_N_9_O_11_^+^ 936.6128. ^1^H NMR (MeOD-*d_4_*, 400 MHz) δ: 8.19 (s, 1 H, H-adenine), 8.11 (s, 1 H, H-adenine), 5.11–5.04 (m, 2 H, H-3, H-13), 4.93 (q, 2 H, -C**H_2_**-A), 4.60 (s, 1 H, H-11), 4.20 (d, *J* = 7.2 Hz, 1 H, H-1′), 3.66–3.19 (m, 6 H, H-5′, -CONH-C**H**_2_(CH_2_)_6_C**H_2_**-, H-5, H-2′), 3.04–2.95 (m, 3 H, H-10, H-2, -CONHC**H**_2_-), 2.60–2.55 (m, 1 H, H-3′), 2.40–2.33 (m, 7 H, -N(CH_3_)_2_, H-9a), 2.23 (s, 3 H, N-CH_3_), 2.20–2.13 (m, 2 H, H-4, H-9b), 1.84–1.78 (m, 2 H, H-8, H-14eq), 1.72–1.63 (m, 3 H, H-4′a, H-14ax, H-7a), 1.52–1.50 (m, 4 H, -CONHCH_2_C**H_2_**(CH_2_)_4_C**H_2_**CH_2_-), 1.46 (s, 3 H, 12-CH_3_), 1.42–1.25 (m, 10 H, -CONHCH_2_CH_2_C**H_2_**C**H_2_**C**H_2_**C**H_2_**CH_2_CH_2_-, H-7b, H-4′b), 1.25–1.16 (m, 9 H, 6-CH_3_, 2-CH_3_, 5′-CH_3_), 1.09–1.06 (m, 6 H, 10-CH_3_, 4-CH_3_), 0.94-0.89 (m, 6 H, 8-CH_3_, 15-CH_3_). ^13^C NMR (MeOD-*d_4_*, 176 MHz) δ: 175.7, 167.1, 157.4, 155.9, 152.4, 149.6, 142.2, 118.3, 101.3, 83.3, 78.7, 77.4, 75.8, 74.1, 73.4, 71.1, 69.7, 68.5, 64.4, 62.4, 43.1, 40.5, 39.6, 39.3, 35.6, 30.9, 29.6, 28.9, 28.8, 26.4, 26.1, 25.1, 20.5, 20.3, 20.1, 16.1, 14.6, 9.8, 8.2, 5.9.

#### 3.1.20. General Procedures for the Synthesis of Target Compounds **29G**–**30G**, **32G** and **29C**–**30C**

Compound **20**–**21** or **23** (1 eq), compound **12** or **14** (1.2 eq) and HOBT (1.1 eq) were dissolved in CH_2_Cl_2_, then the solution was cooled to 0 °C and DCC (1.2 eq) was added. The ice bath was removed after 1.5 h, and the stirring was continued for another 2.5 h at rt. The precipitated DCU was removed through filtration and washed with DCM. Then, the combined filtrate was washed with water and brine and then concentrated. The resulting mixture was dissolved in methanol and stirred under reflux for 2 h. The methanol was concentrated, and the crude product was purified through column chromatography (CH_2_Cl_2_/MeOH/NH_3_·H_2_O = 10:0.5:0.05). The resulting compound was dissolved in MeOH, then Pd/C (15% wt) was added under H_2_. After stirring for 24 h at room temperature, the Pd/C was filtered. The filtrate was concentrated and purified through column chromatography to afford compounds **29G**–**30G, 32G** and **29C**–**30C**.

##### 3-*O*-descladinosyl-3-*O*-(5-(2-(guanin-9-yl)acetamido)pentyl)carbamoyl azithromycin -11, 12-cyclic carbonate (**29G**)

Compound **20** (0.365 g, 0.49 mmol), compound **12** (0.176 g, 0.59 mmol), DCC (0.100 g, 0.59 mmol) and HOBT (0.073 g, 0.54 mmol) were dissolved in DCM according to the general procedure. The crude product was purified through column chromatography (CH_2_Cl_2_/MeOH/NH_3_·H_2_O = 10:0.3:0.05) to afford compound **29G** (42.6 mg, 0.05 mmol, 31.3%). Melting point: 138.7–140.0 °C. HRMS (ESI) (M + H)^+^ *m*/*z* 936.5394, calcd for C_44_H_74_N_9_O_13_^+^ 936.5401. ^1^H NMR (DMSO-*d_6_*, 400 MHz) δ: 10.58 (s, 1 H, -NH-guanine), 8.16 (s, 1 H, -CONH-), 7.59 (s, 1 H, H-guanine),7.33(s, 1 H, -*O*-CONH-), 6.46 (s, 1 H, -NH_2_-), 5.30 (s, 1 H, -OH), 4.92–4.86 (m, 2 H, H-3, H-13), 4.61 (s, 2 H, -C**H_2_**-G), 4.43 (s, 1 H, H-11), 4.19 (d, *J* = 7.2 Hz, 1 H, H-1′), 3.40–3.02 (m, 7 H, H-5′, -CONH-C**H_2_**(CH_2_)_3_C**H_2_**-, H-5, H-2′), 2.92–2.82 (m, 2 H, H-10, H-2), 2.28–2.25 (m, 1 H, H-3′), 2.18–2.10 (m, 1 H, H-9a), 2.10 (s, 3 H, N-CH_3_), 2.06–2.04 (m, 1 H, H-4), 1.78–1.61 (m, 4 H, H-8, H-14eq, H-14ax, H-7a), 1.43–1.40 (m, 7 H, -CONHCH_2_C**H_2_**CH_2_C**H_2_**CH_2_-, 12-CH_3_), 1.38–1.24 (m, 5 H, H-7b, H-4′b, H-4′a, -CONHCH_2_CH_2_C**H_2_**CH_2_CH_2_-), 1.11–1.05 (m, 9 H, 6-CH_3_, 2-CH_3_, 5′-CH_3_), 1.00–0.96 (m, 6 H, 10-CH_3_, 4-CH_3_), 0.86–0.82 (m, 6 H, 8-CH_3_, 15-CH_3_). ^13^C NMR (DMSO-*d_6_*, 100 MHz) δ: 175.0, 166.7, 157.3, 156.8, 154.1, 153.3, 151.9, 138.8, 116.6, 101.1, 86.0, 85.7, 83.2, 79.7, 77.5, 76.0, 73.1, 68.0, 66.8, 65.2, 60.5, 45.2, 42.9, 40.6, 40.5, 39.1, 35.9, 34.8, 30.7, 29.5, 29.1, 26.3, 25.4, 24.0, 22.0, 21.5, 21.3, 15.5, 14.1, 10.2, 9.6, 5.8.

##### 3-*O*-descladinosyl-3-*O*-(6-(2-(guanin-9-yl)acetamido)hexyl)carbamoyl azithromycin-11, 12-cyclic carbonate (**30G**)

Compound **21** (0.408 g, 0.54 mmol), compound **12** (0.194 g, 0.65 mmol), DCC (0.110 g, 0.65 mmol) and HOBT (0.080 g, 0.59 mmol) were dissolved in DCM according to the general procedure. The crude product was purified through column chromatography (CH_2_Cl_2_/MeOH/NH_3_·H_2_O = 10:0.3:0.05) to afford compound **30G** (28.5 mg, 0.03 mmol, 15.8%). Melting point: 132.8–134.5 °C. HRMS (ESI) (M + H)^+^ *m*/*z* 950.5568, calcd for C_45_H_76_N_9_O_13_^+^ 950.5557. ^1^H NMR (DMSO-*d_6_*, 400 MHz) δ: 10.52 (s, 1 H, -NH-guanine), 8.11 (s, 1 H, -CONH-), 7.59 (s, 1 H, H-guanine),7.28 (s, 1 H, -*O*-CONH-), 6.40 (s, 1 H, -NH_2_-), 5.32 (s, 1 H, -OH), 4.92–4.86 (m, 2 H, H-3, H-13), 4.60 (s, 2 H, -C**H_2_**-G), 4.43 (s, 1 H, H-11), 4.12 (d, *J* = 7.2 Hz, 1 H, H-1′), 3.41–3.02 (m, 7 H, H-5′, -CONH-C**H_2_**(CH_2_)_4_C**H_2_**-, H-5, H-2′), 2.92–2.82 (m, 2 H, H-10, H-2), 2.25–2.17 (m, 7 H, -N(CH_3_)_2_, H-9a), 2.10 (s, 3 H, N-CH_3_), 2.04–2.01 (m, 1 H, H-4), 1.78–1.57 (m, 4 H, H-8, H-14eq, H-14ax, H-7a), 1.43 (s, 3 H, 12-CH_3_), 1.34–1.26 (m, 10 H, H-7b, H-4′b, -CONHCH_2_C**H_2_**C**H_2_**C**H_2_**C**H_2_**CH_2_-), 1.08–1.04 (m, 9 H, 6-CH_3_, 2-CH_3_, 5′-CH_3_), 0.99–0.95 (m, 6 H, 10-CH_3_, 4-CH_3_), 0.86–0.82 (m, 6 H, 8-CH_3_, 15-CH_3_). ^13^C NMR (DMSO-*d_6_*, 100 MHz) δ: 175.0, 167.5, 158.0, 157.3, 154.0, 153.5, 152.1, 139.0, 115.8, 100.9, 86.4, 85.0, 83.2, 78.2, 76.0, 73.4, 70.0, 68.3, 67.2, 65.1, 61.4, 45.0, 43.1, 40.9, 40.4, 39.0, 35.9, 33.8, 30.3, 29.4, 28.9, 26.0, 25.9, 25.7, 24.8, 21.3, 20.3, 19.9, 14.7, 12.1, 9.2, 8.2, 3.8.

##### 3-*O*-descladinosyl-3-*O*-(8-(2-(guanin-9-yl)acetamido)octyl)carbamoyl azithromycin-11, 12-cyclic carbonate (**32G**)

Compound **23** (0.343 g, 0.44 mmol), compound **12** (0.261 g, 0.52 mmol), DCC (0.109 g, 0.52 mmol) and HOBT (0.065 g, 0.48 mmol) were dissolved in DCM according to the general procedure. The crude product was purified through column chromatography (CH_2_Cl_2_/MeOH/NH_3_·H_2_O = 10:0.25:0.05) to afford compound **32G** (62.7 mg, 0.06 mmol, 14.6%). Melting point: 131.6-133.0 °C. HRMS (ESI) (M + H)^+^ *m*/*z* 978.5842, calcd for C_47_H_80_N_9_O_13_^+^ 978.5870. ^1^H NMR (MeOD-*d_4_*, 400 MHz) δ: 7.70 (s, 1 H, H-guanine), 5.10–5.04 (m, 2 H, H-3, H-13), 4.74 (s, 2 H, -C**H_2_**-G), 4.43 (s, 1 H, H-11), 4.59 (d, *J* = 7.2 Hz, 1 H, H-1′), 3.66 (s, 1 H, H-5), 3.44–3.40 (m, 1 H, H-5′), 3.28–3.18 (m, 4 H, H-2′, -CONH-C**H**_2_(CH_2_)_6_C**H_2_**-), 3.06–2.91 (m, 3 H, H-10, H-2, -CONHC**H**_2_-), 2.67-2.60 (m, 1 H, H-3′), 2.40–2.36 (m, 7 H, -N(CH_3_)_2_, H-9a), 2.23 (s, 3 H, N-CH_3_), 2.21–2.12 (m, 2 H, H-4, H-9a), 1.84–1.78 (m, 2 H, H-8, H-14eq), 1.74-1.63 (m, 3 H, H-4′a, H-14ax, H-7a), 1.51-1.49 (m, 4 H, -CONH-CH_2_C**H_2_**(CH_2_)_4_C**H_2_**CH_2_-), 1.47 (s, 3 H, 12-CH_3_), 1.32–1.24 (m, 10 H, H-7b, H-4′b, -CONHCH_2_CH_2_C**H_2_**C**H_2_**C**H_2_**C**H_2_**CH_2_CH_2_-), 1.20–1.16 (m, 9 H, 6-CH_3_, 2-CH_3_, 5′-CH_3_), 1.09-1.06 (m, 6 H, 10-CH_3_, 4-CH_3_), 0.94–0.89 (m, 6 H, 8-CH_3_, 15-CH_3_). ^13^C NMR (MeOD-*d_4_*, 176 MHz) δ: 175.0, 167.4, 158.0, 157.3, 154.0, 153.5, 152.1, 139.0, 115.8, 101.4, 86.5, 85.0, 83.4, 78.3, 75.9, 73.4, 70.9, 68.6, 67.2, 64.5, 61.4, 45.0, 43.1, 40.9, 40.5, 39.5, 39.2, 35.9, 33.8, 30.8, 29.6, 28.9, 26.5, 25.8, 24.8, 21.3, 20.3, 20.0, 14.7, 12.1, 9.2, 8.2, 3.8.

##### 3-*O*-descladinosyl-3-*O*-(5-(2-(cytosin-1-yl)acetamido)pentyl)carbamoyl azithromycin -11,12-cyclic carbonate (**29C**)

Compound **20** (0.406 g, 0.55 mmol), compound **14** (0.200 g, 0.66 mmol), DCC (0.112 g, 0.66 mmol) and HOBT (0.082 g, 0.61 mmol) were dissolved in DCM according to the general procedure. The crude product was purified through column chromatography (CH_2_Cl_2_/MeOH/NH_3_·H_2_O = 10:0.25:0.05) to afford compound **29C** (82.3 mg, 0.10 mmol, 51.2%). Melting point: 135.2-137.0 °C. HRMS (ESI) (M + H)^+^ *m*/*z* 896.5369, calcd for C_43_H_74_N_7_O_13_^+^ 896.5339. ^1^H NMR (CDCl_3_, 400 MHz) δ: 7.60 (s, 1 H, -CONH-), 7.37 (d, *J* = 7.2 Hz, 1 H, H-cytosine), 6.22 (s, 1 H, -OH), 5.84 (d, *J* = 7.2 Hz, 2 H, H-cytosine, -*O*-CONH-), 5.10 (d, *J* =10.4 Hz, 1 H, H-3), 5.04 (dd, *J* = 10.8, 2.0 Hz, 1 H, H-13), 4.59 (s, 1 H, H-11), 4.46–4.35 (m, 2 H, -C**H_2_**-cytosine), 4.14 (d, *J* = 7.2 Hz, 1 H, H-1′), 3.56–2.98 (m, 6 H, H-5′, -CONH-C**H**_2_(CH_2_)_3_C**H_2_**-, H-5, H-2′), 2.88–2.83 (m, 3 H, H-10, H-2, -CONH-C**H**_2_-), 2.51–2.50 (m, 1 H, H-3′), 2.38–2.29 (m, 7 H, H-9a, -N(CH_3_)_2_), 2.25 (s, 3 H, N-CH_3_), 2.12–2.00 (m, 2 H, H-4, H-9b), 1.89–1.81 (m, 2 H, H-14eq, H-7a), 1.68–1.49 (m, 7 H, H-4′a, -CO-NH-CH_2_C**H_2_**CH_2_C**H_2_**CH_2_-, H-8, H-14ax), 1.42 (s, 3 H, 12-CH_3_), 1.25–1.16 (m, 13 H, H-7b, H-4′b, -CO-NH-CH_2_CH_2_C**H_2_**CH_2_CH_2_-, 6-CH_3_, 5′-CH_3_, 2-CH_3_), 1.07–1.04 (m, 6 H, 10-CH_3_, 4-CH_3_), 0.92–0.87 (m, 6 H, 8-CH_3_, 15- CH_3_). ^13^C NMR (CDCl_3_, 100 MHz) δ: 174.8, 167.9, 166.4, 155.6, 153.4, 146.4, 103.0, 95.2, 86.7, 86.3, 84.9, 78.5, 75.9, 73.2, 70.8, 69.1, 68.0, 65.6, 61.8, 53.0, 43.3, 41.9, 40.6, 40.4, 39.3, 36.0, 34.9, 29.5, 29.1, 28.6, 26.0, 25.8, 23.7, 21.7, 21.4, 21.2, 15.6, 13.5, 10.2, 9.4, 5.0.

##### 3-*O*-descladinosyl-3-*O*-(6-(2-(cytosin-1-yl)acetamido)hexyl)carbamoyl azithromycin-11, 12-cyclic carbonate (**30C**)

Compound **21** (0.376 g, 0.50 mmol), compound **14** (0.182 g, 0.60 mmol), DCC (0.102 g, 0.60 mmol) and HOBT (0.074 g, 0.55 mmol) were dissolved in DCM according to the general procedure. The crude product was purified through column chromatography (CH_2_Cl_2_/MeOH/NH_3_·H_2_O = 10:0.25:0.05) to afford compound **30C** (65.6 mg, 0.07 mmol, 46.1%). Melting point: 131.9-133.4 °C. HRMS (ESI) (M + H)^3+^ *m*/*z* 304.1881, calcd for C_44_H_76_N_7_O_13_^3+^ 304.1880. ^1^H NMR (CDCl_3_, 400 MHz) δ: 7.40 (d, *J* = 7.2 Hz, 1 H, H-cytosine), 6.16 (s, 1 H, -OH), 5.87 (d, *J* = 6.8 Hz, 1 H, H-cytosine), 5.69 (s, 1 H, -*O*-CONH-), 5.11 (d, *J* =10.4 Hz, 1 H, H-3), 5.05 (dd, *J* = 10.8, 2.0 Hz, 1 H, H-13), 4.60 (s, 1 H, H-11), 4.49-4.38 (m, 2 H, -C**H_2_**-cytosine), 4.17 (d, *J* = 7.2 Hz, 1 H, H-1′), 3.56–3.02 (m, 6 H, H-5′, -CONH-C**H**_2_(CH_2_)_4_C**H_2_**-, H-5, H-2′), 2.88–2.83 (m, 3 H, H-10, H-2, -CONH-C**H**_2_-), 2.70 (m, 1 H, H-3′), 2.38–2.32 (m, 7 H, -N(CH_3_)_2,_ H-9a), 2.25 (s, 3 H, N-CH_3_), 2.14–2.12 (m, 1 H, H-4), 2.06–2.00 (m, 1 H, H-9b), 1.89–1.87 (m, 2 H, H-8, H-14eq), 1.73–1.70 (m, 1 H, H-4′a), 1.61–1.58 (m, 2 H, H-14ax, H-7a), 1.52–1.48 (m, 4 H, -CONHCH_2_C**H_2_**(CH_2_)_2_C**H_2_**CH_2_-), 1.42 (s, 3 H, 12-CH_3_), 1.31–1.17 (m, 15 H, H-7b, H-4′b, -CONHCH_2_CH_2_C**H_2_**C**H_2_**CH_2_CH_2_-, 6-CH_3_, 2-CH_3_, 5′-CH_3_), 1.08–1.05 (m, 6 H, 10-CH_3_, 4-CH_3_), 0.92–0.88 (m, 6 H, 8-CH_3_, 15-CH_3_). ^13^C NMR (CDCl_3_, 100 MHz) δ: 174.8, 167.7, 166.2, 156.5, 153.4, 146.2, 103.2, 95.2, 87.1, 86.3, 84.9, 78.5, 75.9, 73.1, 70.8, 69.1, 68.0, 65.7, 61.8, 53.3, 43.3, 42.0, 40.5, 40.4, 40.3, 39.1, 36.0, 34.9, 29.8, 26.0, 25.9, 25.8, 21.7, 21.4, 21.2, 15.6, 13.5, 10.2, 9.4, 5.0.

#### 3.1.21. General Procedures for the Synthesis of Intermediates **36U** and **36A**

Compound **23** (1 eq) was dissolved in CH_2_Cl_2_, then NaBH(OAc)_3_ (2 eq), ZnCl_2_ (1.2 eq) and compound **5** or **10** (2 eq) were added to the solution. After stirring for 2 h at room temperature, the mixture was washed with water and brine and then concentrated. The resulting mixture was dissolved in methanol and stirred at 60 °C for 2 h. The methanol was concentrated, and the crude product was purified through column chromatography (CH_2_Cl_2_/EtOH/NH_3_·H_2_O = 10:0.3:0.05) to afford intermediates **36U** and **36A**.

##### 3-*O*-descladinosyl-3-*O*-(8-((2-(*N*^6^-(di-tert-butyloxycarbonyl)adenin-9-yl)ethyl)amino)octyl)carbamoylazithromycin-11,12-cyclic carbonate (**36A**)

Compound **36A** (100 mg, 0.09 mmol, 36.0%) was prepared from compound **23** (0.200 g, 0.25 mmol), compound **10** (0.189 g, 0.50 mmol), NaBH(OAc)_3_ (0.108 g, 0.50 mmol) and ZnCl_2_ (0.034 g, 0.30 mmol) according to the general procedure. HRMS (ESI) (M + H)^+^ *m*/*z* 1148.7133, calcd for C_57_H_98_N_9_O_15_^+^ 1148.7177. ^1^H NMR (MeOD-*d_4_*, 400 MHz) δ: 8.84 (s, 1 H, H-adenine), 8.54 (s, 1 H, H-adenine), 5.10–5.04 (m, 2 H, H-3, H-13), 4.60 (s, 1 H, H-11), 4.48 (t, *J* = 6.4 Hz, 2 H, -C**H_2_**-A), 4.20 (d, *J* = 7.2 Hz, 1 H, H-1′), 3.66 (s, 1 H, H-5), 3.45–3.40 (m, 1 H, H-5′), 3.28–3.23 (m, 2 H, -CONH-C**H**_2_-, H-2′), 3.11 (t, *J* = 6.4 Hz, 2 H, -C**H_2_**-CH_2_-A),3.05–2.90 (m, 3 H, H-2, H-10, -CONH-C**H**_2_-), 2.60-2.57 (m, 3 H, -C**H_2_**-NH-CH_2_-, H-3′), 2.37-2.34 (m, 7 H, -N(CH_3_)_2_, H-9a), 2.23 (s, 3 H, N-CH_3_), 2.20–2.10 (m, 2 H, H-4, H-9b), 1.90–1.78 (m, 2 H, H-8, H-14eq), 1.69–1.63 (m, 3 H, H-4′a, H-14ax, H-7a), 1.51–1.49 (m, 2 H, -CONHCH_2_C**H_2_**-), 1.46 (s, 3 H, 12-CH_3_), 1.39 (m, 20 H, -C**H_2_**CH_2_-NH-, -(Boc)_2_),1.31–1.30 (m, 10 H, H-7b, H-4′b, -CONHCH_2_CH_2_C**H_2_**C**H_2_**C**H_2_**C**H_2_**CH_2_CH_2_-), 1.20–1.16 (m, 9 H, 6-CH_3_, 2-CH_3_, 5′-CH_3_), 1.09-1.06 (m, 6 H, 10-CH_3_, 4-CH_3_), 0.94–0.89 (m, 6 H, 8-CH_3_, 15-CH_3_).

#### 3.1.22. General Procedures for the Synthesis of Target Compounds **37U** and **37A**

Intermediate **36U** or **36A** was dissolved in EtOH/HCl (8:2 mL) and the solution was stirred for 30 min. Then, the pH was adjusted to 10 with NH_3_·H_2_O, and then CH_2_Cl_2_ was added for extraction. The organic phase was washed with water and brine once and then purified through column chromatography (CH_2_Cl_2_/MeOH/NH_3_·H_2_O = 10:0.7:0.05) to afford compounds **37U** and **37A**.

##### 3-*O*-descladinosyl-3-*O*-(8-((2-(uracil-1-yl)ethyl)amino)octyl)carbamoyl azithromycin-11,12-cyclic carbonate (**37U**)

Compound **37U** (22.0 mg, 0.02 mmol, 29.7%) was prepared from compound **36U** (80 mg, 0.08 mmol) according to the general procedure. Melting point: 129.5-130.6 °C. HRMS (ESI) (M + H)^+^ *m*/*z* 925.5859, calcd for C_46_H_81_N_6_O_13_^+^ 925.5856. ^1^H NMR (MeOD-*d_4_*, 400 MHz) δ: 7.55 (d, *J* = 7.6 Hz, 1 H, H-uracil), 5.63 (d, *J* = 8.0 Hz, 1 H, H-uracil), 5.10–5.04 (m, 2 H, H-3, H-13), 4.60 (s, 1 H, H-11), 4.20 (d, *J* = 7.6 Hz, 1 H, H-1′), 3.85 (t, *J* = 6.4 Hz, 2 H, -C**H_2_**-U), 3.66 (s, 1 H, H-5), 3.44–3.39 (m, 1 H, H-5′), 3.27–3.18 (m, 2 H, -CONH-C**H**_2_-, H-2′), 3.06–2.91 (m, 3 H, -CONH-C**H**_2_-, H-10, H-2), 2.86 (t, *J* = 6.4 Hz, 2 H, -C**H_2_**-CH_2_-U), 2.60–2.57 (m, 3 H, -C**H_2_**-NH-CH_2_-, H-3′), 2.40–2.34 (m, 7 H, -N(CH_3_)_2_, H-9a), 2.24 (s, 3 H, N-CH_3_), 2.18–2.13 (m, 2 H, H-4, H-9b), 1.88–1.78 (m, 2 H, H-8, H-14eq), 1.72–1.63 (m, 3 H, H-4′a, H-14ax, H-7a), 1.52–1.47 (m, 7 H, 12-CH_3_, -CONHCH_2_C**H_2_**(CH_2_)_4_C**H_2_**CH_2_-), 1.42–1.23 (m, 10 H, H-7b, H-4′b, -CONHCH_2_CH_2_C**H_2_**C**H_2_**C**H_2_**C**H_2_**CH_2_CH_2_-), 1.20–1.16 (m, 9 H, 6-CH_3_, 2-CH_3_, 5′-CH_3_), 1.09–1.06 (m, 6 H, 10-CH_3_, 4-CH_3_), 0.94–0.90 (m, 6 H, 8-CH_3_, 15-CH_3_). ^13^C NMR (MeOD-*d_4_*, 176 MHz) δ: 174.9, 165.5, 157.3, 153.5, 151.6, 146.1, 101.4, 100.7, 86.5, 85.0, 83.4, 78.3, 75.9, 73.4, 71.1, 68.6, 67.2, 64.4, 61.4, 49.1, 43.1, 40.9, 40.5, 39.6, 35.9, 33.8, 30.9, 29.6, 29.2, 29.1, 29.0, 26.9, 26.5, 25.7, 24.8, 21.3, 20.3, 20.0, 14.7, 12.1, 9.2, 8.2, 3.7.

##### 3-*O*-descladinosyl-3-*O*-(8-((2-(adenin-9-yl)ethyl)amino)octyl)carbamoyl azithromycin -11, 12-cyclic carbonate (**37A**)

Compound **37A** (30.8 mg, 0.03 mmol, 36.1%) was prepared from compound **36A** (100 mg, 0.09 mmol) according to the general procedure. Melting point: 125.7-127.1 °C. HRMS (ESI) (M + H)^+^ *m*/*z* 948.6117, calcd for C_47_H_82_N_9_O_11_^+^ 948.6128. ^1^H NMR (MeOD-*d_4_*, 400 MHz) δ: 8.20 (s, 1 H, H-adenine), 8.12 (s, 1 H, H-adenine), 5.10–5.04 (m, 2 H, H-3, H-13), 4.59 (s, 1 H, H-11), 4.34 (t, *J* = 6.4 Hz, 2 H, -C**H_2_**-A), 4.20 (d, *J* = 7.2 Hz, 1 H, H-1′), 3.66 (s, 1 H, H-5), 3.45–3.40 (m, 1 H, H-5′), 3.26–3.23 (m, 2 H, -CONH-C**H**_2_-, H-2′), 3.06-3.03 (m, 5 H, -C**H_2_**-CH_2_-A, H-2, H-10, -CONH-C**H**_2_-), 2.60–2.56 (m, 3 H, -C**H_2_**-NH-CH_2_-, H-3′), 2.40–2.34 (m, 7 H, -N(CH_3_)_2_, H-9a), 2.23 (s, 3 H, N-CH_3_), 2.21–2.11 (m, 2 H, H-4, H-9b), 1.89–1.78 (m, 2 H, H-8, H-14eq), 1.71–1.63 (m, 3 H, H-4′a, H-14ax, H-7a), 1.49–1.43 (m, 7 H, -CONHCH_2_C**H_2_**(CH_2_)_4_C**H_2_**CH_2_-, 12-CH_3_), 1.29–1.25 (m, 10 H, H-7b, H-4′b, -CONHCH_2_CH_2_C**H_2_**C**H_2_**C**H_2_**C**H_2_**CH_2_CH_2_-), 1.20–1.16 (m, 9 H, 6-CH_3_, 2-CH_3_, 5′-CH_3_), 1.09-1.06 (m, 6 H, 10-CH_3_, 4-CH_3_), 0.94-0.89 (m, 6 H, 8-CH_3_, 15-CH_3_). ^13^C NMR (MeOD-*d_4_*, 176 MHz) δ: 174.9, 157.3, 155.9, 153.5, 152.3, 149.5, 141.6, 118.7, 101.4, 86.5, 85.0, 83.4, 78.3, 75.9, 73.4, 71.0, 68.6, 67.2, 64.4, 61.4, 48.9, 48.2, 43.2, 43.1, 40.9, 40.5, 39.6, 35.9, 33.8, 30.8, 29.6, 29.1, 29.0, 28.9, 26.8, 26.5, 24.8, 21.3, 20.3, 20.0, 14.7, 12.1, 9.2, 8.3, 3.8.

### 3.2. Antibacterial Assay

Minimum inhibitory concentrations (MICs) were determined using the microbroth dilution method, which was recommended by the Clinical and Laboratory Standards Institute. Standard ATCC strains and standardized antibiotics were used as quality controls in each experiment where stock solutions of the target compounds and clinical drugs were prepared to different concentrations of 256, 128, 64, 32, 16, 8, 4, 2, 1 and 0.25 μg/mL. The fresh bacterial strains purified through plate activation in advance were used for testing. The final concentration of the tested bacterial solution is 5 × 10^5^ CFU/mL.

### 3.3. Docking Methods

The molecular docking studies of the ligands (**37A** and **32A**) against target G2099A mutant 50S ribosomal subunit (PDB ID: 1YHQ) were performed utilizing Autodock VINA in YASARA (YASARA Biosciences GmbH, Vienna, VIE, AUT) [36,37]. To remove bumps and ascertain the covalent geometry of the ligands, the structures were all energy-minimized with the NOVA force field [39]. The dockings were undertaken by defining the simulation cell box of size 30 Å × 30 Å × 30 Å for the G2099A mutant 50S ribosomal subunit. Then, the docking studies on the aptamer were carried out through the built-in docking simulation macro ‘dock_run.mcr’ using an AMBER03 force field with 25 poses and 9 clusters for each situation [40].

### 3.4. Molecular Dynamics

To justify whether the models constructed from protein–ligand docking were reliable, molecular dynamics (MD) simulations of the bindings between G2099A mutant 50S ribosomal subunit with the ligands (**37A** and **32A**) were performed. The AMBER14 force field was utilized for the MD simulation, as implemented in the YASARA program. For the treatment of long-range coulomb forces beyond an 8 Å cutoff, the MD simulation used periodic boundary conditions and the particle-mesh Ewald method. NaCl was used at a concentration of 0.9%, and the density of HOH was 0.997 g/mL in the MD cell. No restraints were applied during the MD simulation using the settings employed in the second equilibration dynamics. Energies and coordinates every 100 ps were saved with a total simulation length of 40–50 ns under a constant temperature (298 K) and uncontrolled pressure in an NVT ensemble. The structural stability of the protein–ligand complex was examined by analyzing the average values of the potential energy with root mean square deviation (RMSD) throughout the trajectory. The RMSD profiles of all MD structures (Figure S1) show that the variation in the RMSD values tends to be stable (<1 Å) after 40–50 ns, indicating that equilibrium was reached, and the last MD structure was chosen for the analysis.

## 4. Conclusions

In summary, macrolides conjugating with nucleobases exhibited considerably low activities against clinical isolates due to poor accumulation in the cell, which was confirmed in the presence of menadione (VK_3_) that could inhibit the efflux pumps and alter the fluidity of the bacterial membrane of *S. aureus*. Thus, we turned to exploit an engineered *E. coli* SQ110 in combination with its rRNA-mutated strains to investigate the SARs of the ribosome-targeting inhibitors. Notably, **34A** was the most potent against A2059G-mutated strains (**34A** 16 μg/mL vs. telithromycin 128 μg/mL, azithromycin > 256 μg/mL and clindamycin > 128 μg/mL). Compounds **37A** and **37U** showed weak activities against the A2058G-mutated strain (128 μg/mL vs. telithromycin > 128 μg/mL, azithromycin > 256 μg/mL and clindamycin > 128 μg/mL). Molecular docking suggested additional hydrogen bonding formation between the adenine of the conjugates and the surrounding ribosomal nucleobases.

The conjugates had superior profiles over a family of macrolide-lincosamide-streptogramin B against A2058/2059-mutated strains, which suggested that a suitable extended alkyl–aryl arm derived from the C-3 of macrolactone, perhaps supplied extra opportunities to combat high-level A2058 mutated resistance. Further research on novel conjugates to address the inefficiency of this work against clinical isolates is underway.

## Figures and Tables

**Table 1:**
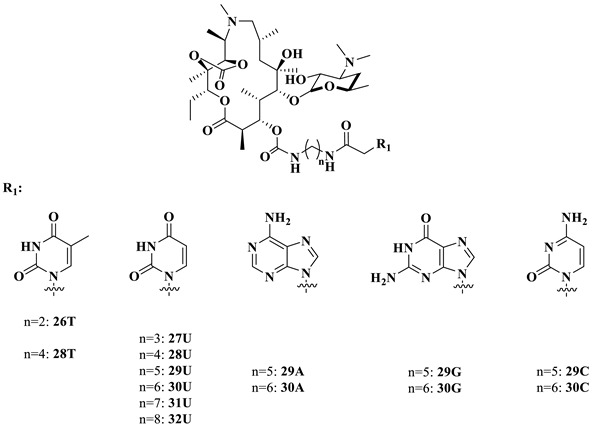
In vitro antibacterial activity of **26–32** against erythromycin-susceptible and -resistant clinical isolates.

Compd.	MICs against Clinical Isolates (μg/mL)						
	*S. pneumoniae*			*S. aureus*		*S. pyogenes*	*H. influenzae*
	ATCC 49619	PU 09	07P390	PU 32	15B196	12-206	ATCC 49247
	EryS ^a^	*mef* ^b^	c-MLS_B_^c^	i-MLS_B_^d^	c-MLS_B_	c-MLS_B_	EryS
**26T**	>256	>256	>256	>256	>256	>256	>256
**28T**	>256	>256	>256	>256	>256	>256	>256
**27U**	>256	>256	>256	>256	>256	>256	>256
**28U**	256	>256	>256	>256	>256	>256	>256
**29U**	128	>256	>256	>256	>256	>256	>256
**30U**	128	>256	>256	>256	>256	>256	>256
**31U**	64	256	>256	>256	>256	>256	>256
**32U**	16	128	>256	>256	>256	>256	>256
**29A**	32	256	>256	>256	>256	>256	>256
**30A**	32	128	>256	>256	>256	>256	128
**29G**	64	>256	>256	>256	>256	>256	>256
**30G**	64	>256	>256	>256	>256	>256	>256
**29C**	256	>256	>256	>256	>256	>256	>256
**30C**	256	>256	>256	>256	>256	>256	>256
Azithromycin	0.25	8	>256	>256	>256	>256	2

^a^ EryS: erythromycin-susceptible strains. ^b^ *mef*: macrolide efflux genotypes. ^c^ c-MLS_B_: constitutive macrolide-lincosamide-streptogramin B-resistance strains. ^d^ i-MLS_B_: inducible macrolide-lincosamide-streptogramin B-resistance strains.

**Table 2:**
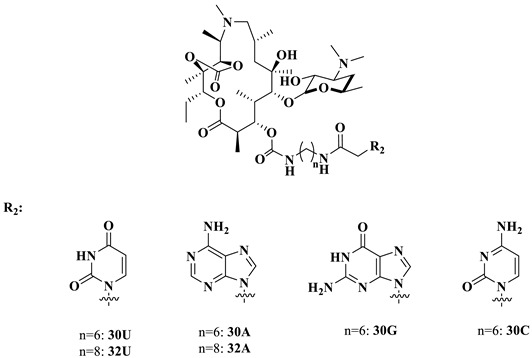
In vitro antibacterial activity of **30** and **32** against some standard, engineered and mutant *E. coli* strains.

Compd.	MICs against *E. coli* (μg/mL)				
	ATCC25922	SQ110DTC ^a^	DTC—A2059G	SQ110LPTD ^b^	LPTD—A2058G
**30U**	>256	2	>256	16	>256
**30A**	>256	1	>256	4	>256
**30G**	>256	2	>256	32	256
**30C**	>256	4	>256	32	>256
**32U**	256	2	>256	8	256
**32A**	128	2	>128	2	>128
Azithromycin	4	≤0.5	>256	≤0.5	>256

^a^ SQ110DTC: *E. coli* SQ110 without multidrug transporters TolC. ^b^ SQ110LPTD: *E. coli* SQ110 without intact outer membrane.

**Table 3:**
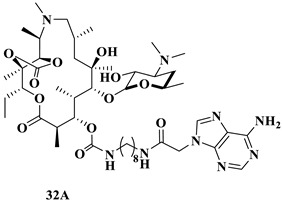
In vitro antibacterial activity of **32A** alone or in combination with menadione against *S. aureus*.

**Compd.**	**MICs against *S. aureus* (μg/mL)**		
	**ATCC29213**	**PU32**	**15B196**
	**EryS**	**i-MLS_B_, MRSA**	**c-MLS_B_, MRSA**
VK_3_^a^	16	16	16
**32A**	64	>128	>128
**32A** + VK_3_ (MIC/8)	32	>128	>128
**32A** + VK_3_ (MIC/4)	16	≤0.25	>128
3-OH azithromycin ^b^	>128	>128	>128
3-OH azithromycin + VK_3_ (MIC/8)	>128	>128	>128
3-OH azithromycin + VK_3_ (MIC/4)	128	≤0.25	>128
Azithromycin	1	32	>128
Azithromycin + VK_3_ (MIC/8)	0.5	2	>128
Azithromycin + VK_3_ (MIC/4)	≤0.25	≤0.25	>128

^a^ VK_3_: menadione. ^b^ 3-OH azithromycin: 3-*O*-descladinose-3-OH azithromycin.

**Table 4:**
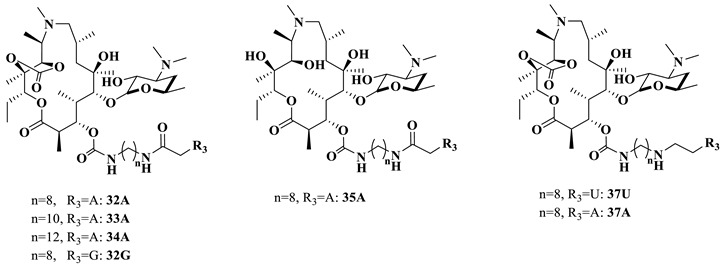
In vitro antibacterial activity of **32G**, **32A–35A** and **37** against some engineered and mutant *E. coli* strains.

Compd.	MICs against *E. coli* (μg/mL)				
	BW25113	SQ110DTC ^a^	DTC—A2059G	SQ110LPTD ^b^	LPTD—A2058G
**32A**	256	1	256	4	256
**33A**	64	≤0.5	64	2	>256
**34A**	>256	1	16	2	>256
**32G**	>256	2	256	16	256
**35A**	>256	1	>256	16	>256
**37U**	128	≤0.5	256	2	128
**37A**	64	0.5	128	1	128
Azithromycin	4	≤0.5	>256	≤0.5	>256
Clindamycin	NT ^c^	4	>128	16	>128
Telithromycin	NT	NT	128	NT	>128

^a^ SQ110DTC: *E. coli* SQ110 without multidrug transporters TolC. ^b^ SQ110LPTD: *E. coli* SQ110 without intact outer membrane. ^c^ NT: not tested.

**Table 5:** The binding free energies (kcal/mol) and predicted K_D_ values (nM) between the 50S ribosomal subunit (PDB ID: 1YHQ) and the ligands.

Ligands	Binding Free Energies (kcal/mol)	K_D_ (nM)
**32A**	−11.79	2.27
**33A**	−11.21	6.06
**34A**	−10.46	21.5
**32G**	−11.63	2.98
**35A**	−10.46	21.7
**37U**	−11.52	3.59
**37A**	−11.89	1.92

## Data Availability

All data necessary for the understanding of the research are present in the manuscript. Additional data related to the molecular docking data and NMR spectra are available as Supplementary Material.

## Supplementary Materials

The following supporting information can be downloaded at: https://www.mdpi.com/article/10.3390/molecules28031327/s1, Figure S1: The RMSD profiles of MD simulations of the G2099A mutant 50S ribosomal subunit with the ligands (**37A** and **32A**). Figure S2: Superposition of two complexes including the last structures obtained from the MD and the complexes obtained from docking. Table S1: The interaction of hydrogen bonds between G2099A Large Ribosomal Subunits (PDB ID: 1YHQ) and **37A**. Table S2: The interaction of hydrogen bonds between G2099A mutant 50S ribosomal subunit (PDB ID: 1YHQ) and **32A**. Table S3: The interaction of hydrogen bonds between G2099A mutant 50S ribosomal subunit (PDB ID: 1YHQ) and **37A** after molecular dynamic simulation. Table S4: The interaction of hydrogen bonds between G2099A mutant 50S ribosomal subunit (PDB ID: 1YHQ) and **32A** after molecular dynamic simulation. Table S5: HRMS and NMR spectra checklist for the intermediates and target compounds (**1**–**16** and **26**–**37**). ^1^H and ^13^C NMR Spectra for the representative compounds.

## Sample Availability

Samples of the compounds are not available from the authors.

## References

[1] Wilson D.N. (2014). Ribosome-targeting antibiotics and mechanisms of bacterial resistance. Nat. Rev. Microbiol..

[2] Svetlov M.S., Syroegin E.A., Aleksandrova E.V., Atkinson G.C., Gregory S.T., Mankin A.S., Polikanov Y.S. (2021). Structure of Erm-modified 70S ribosome reveals the mechanism of macrolide resistance. Nat. Chem. Biol..

[3] Dunkle J.A., Xiong L., Mankin A.S., Cate J.H.D. (2010). Structures of the Escherichia coli ribosome with antibiotics bound near the peptidyl transferase center explain spectra of drug action. Proc. Natl. Acad. Sci. USA.

[4] Bulkley D., Innis C.A., Blaha G., Steitz T.A. (2010). Revisiting the structures of several antibiotics bound to the bacterial ribosome. Proc. Natl. Acad. Sci. USA.

[5] Llano-Sotelo B., Dunkle J., Klepacki D., Zhang W., Fernandes P., Cate J.H.D., Mankin A.S. (2010). Binding and Action of CEM-101, a New Fluoroketolide Antibiotic That Inhibits Protein Synthesis. Antimicrob. Agents Chemother..

[6] Liang J.-H., Han X. (2013). Structure-Activity Relationships and Mechanism of Action of Macrolides Derived from Erythromycin as Antibacterial Agents. Curr. Top. Med. Chem..

[7] Janas A., Przybylski P. (2019). 14-and 15-membered lactone macrolides and their analogues and hybrids: Structure, molecular mechanism of action and biological activity. Eur. J. Med. Chem..

[8] Seiple I.B., Zhang Z., Jakubec P., Langlois-Mercier A., Wright P.M., Hog D.T., Yabu K., Allu S.R., Fukuzaki T., Carlsen P.N. (2016). A platform for the discovery of new macrolide antibiotics. Nature.

[9] Magee T.V., Han S., McCurdy S.P., Nguyen T.T., Granskog K., Marr E.S., Maguire B.A., Huband M.D., Chen J.M., Subashi T.A. (2013). Novel 3-O-carbamoyl erythromycin A derivatives (carbamolides) with activity against resistant staphylococcal and streptococcal isolates. Bioorg. Med. Chem. Lett..

[10] Liang J.H., Lv W., Li X.L., An K., Cushman M., Wang H., Xu Y.C. (2013). Synthesis and antibacterial activity of 9-oxime ether non-ketolides, and novel binding mode of alkylides with bacterial rRNA. Bioorg. Med. Chem. Lett..

[11] Han X., Lv W., Guo S.Y., Cushman M., Liang J.H. (2015). Synthesis and structure-activity relationships of novel 9-oxime acylides with improved bactericidal activity. Bioorg. Med. Chem..

[12] Tanikawa T., Asaka T., Kashimura M., Misawa Y., Suzuki K., Sato M., Kameo K., Morimoto S., Nishida A. (2001). Synthesis and antibacterial activity of acylides (3-O-acyl-erythromycin derivatives): A novel class of macrolide antibiotics. J. Med. Chem..

[13] Li X.M., Lv W., Guo S.Y., Li Y.X., Fan B.Z., Cushman M., Kong F.S., Zhang J., Liang J.H. (2019). Synthesis and structure-bactericidal activity relationships of non-ketolides: 9-Oxime clarithromycin 11,12-cyclic carbonate featured with three-to eight-atom-length spacers at 3-OH. Eur. J. Med. Chem..

[14] Yang H.F., Liu K., Jin S.G., Huigens R.W. (2021). Design, synthesis and biological evaluation of a halogenated phenazine-erythromycin conjugate prodrug for antibacterial applications. Org. Biomol. Chem..

[15] Tevyashova A.N., Bychkova E.N., Korolev A.M., Isakova E.B., Mirchink E.P., Osterman I.A., Erdei R., Szucs Z., Bata G. (2019). Synthesis and evaluation of biological activity for dual-acting antibiotics on the basis of azithromycin and glycopeptides. Bioorg. Med. Chem. Lett..

[16] Krajacic M.B., Novak P., Cindric M., Brajsa K., Dumic M., Kujundzic N. (2007). Azithromycin-sulfonamide conjugates as inhibitors of resistant Streptococcus pyogenes strains. Eur. J. Med. Chem..

[17] Paljetak H.C., Verbanac D., Padovan J., Dominis-Kramaric M., Kelneric Z., Peric M., Banjanac M., Ergovic G., Simon N., Broskey J. (2016). Macrolones Are a Novel Class of Macrolide Antibiotics Active against Key Resistant Respiratory Pathogens In Vitro and In Vivo. Antimicrob. Agents Chemother..

[18] Pavlovic D., Mutak S. (2011). Discovery of 4″-Ether Linked Azithromycin-Quinolone Hybrid Series: Influence of the Central Linker on the Antibacterial Activity. ACS Med. Chem. Lett..

[19] Fan B.-Z., Hiasa H., Lv W., Brody S., Yang Z.-Y., Aldrich C., Cushman M., Liang J.-H. (2020). Design, synthesis and structure-activity relationships of novel 15-membered macrolides: Quinolone/quinoline-containing sidechains tethered to the C-6 position of azithromycin acylides. Eur. J. Med. Chem..

[20] Costa A.M., Vilarrasa J. (2000). Hybrids of macrolides and nucleobases or nucleosides. Tetrahedron Lett..

[21] Esteban J., Costa A.M., Cruzado M.C., Faja M., Garcia P., Vilarrasa J. (2006). Clarithromycin-adenine and related conjugates. Tetrahedron Lett..

[22] Vazquez-Laslop N., Klepacki D., Mulhearn D.C., Ramu H., Krasnykh O., Franzblau S., Mankin A.S. (2011). Role of antibiotic ligand in nascent peptide-dependent ribosome stalling. Proc. Natl. Acad. Sci. USA.

[23] Daher S.S., Lee M., Jin X., Teijaro C.N., Wheeler S.E., Jacobson M.A., Buttaro B., Andrade R.B. (2021). Synthesis, Biological Evaluation, and Computational Analysis of Biaryl Side-Chain Analogs of Solithromycin. Chemmedchem.

[24] Orelle C., Carlson S., Kaushal B., Almutairi M.M., Liu H.P., Ochabowicz A., Quan S., Pham V.C., Squires C.L., Murphy B.T. (2013). Tools for Characterizing Bacterial Protein Synthesis Inhibitors. Antimicrob. Agents Chemother..

[25] Dueholm K.L., Egholm M., Behrens C., Christensen L., Hansen H.F., Vulpius T., Petersen K.H., Berg R.H., Nielsen P.E., Buchardt O. (1994). Synthesis of peptide nucleic-acid monomers containing the 4 natural nucleobases—Thymine, cytosine, adenine, and guanine and their oligomerization. J. Org. Chem..

[26] Farèse A., Patino N., Condom R., Dalleu S., Guedj R. (1996). Liquid phase synthesis of a peptidic nucleic acid dimer. Tetrahedron Lett..

[27] Yan M., Ma R., Jia L., Venter H., Ma S. (2017). Synthesis and antibacterial activity of novel 3-O-descladinosylazithromycin derivatives. Eur. J. Med. Chem..

[28] Nielsen P.E., Egholm M., Berg R.H., Buchardt O. (1991). Sequence-selective recognition of DNA by strand displacement with a thymine-substituted polyamide. Science.

[29] Abdelbaky A.S., Prokhorov I.A., Gnuskova E.V., Esipova O.V., Kirillova Y.G. (2019). Convenient and Efficient Syntheses of Peptide Nucleic Acid Purine Monomers. Curr. Org. Chem..

[30] Ashworth I.W., Cox B.G., Meyrick B. (2010). Kinetics and Mechanism of N-Boc Cleavage: Evidence of a Second-Order Dependence upon Acid Concentration. J. Org. Chem..

[31] Bashir-Uddin Surfraz M., Akhtar M., Allemann R.K. (2004). Bis-benzyl protected 6-amino cyclitols are poisonous to Pd/C catalysed hydrogenolysis of benzyl ethers. Tetrahedron Lett..

[32] Sridhar P.R., Anjaneyulu B., Rao B.U. (2021). Regioselective Anomeric O-Benzyl Deprotection in Carbohydrates. Eur. J. Org. Chem..

[33] Liu X.-P., Lv W., Zhao F., Ding J., Zhang J.-R., Xue F., Zhang J.-Z., Liu L.-Y., Cushman M., Li Y. (2022). Design and synthesis of novel macrolones bridged with linkers from 11,12-positions of macrolides. Bioorg. Med. Chem. Lett..

[34] Tintino S.R., Oliveira-Tintino C.D.M., Campina F.F., Weslley Limaverde P., Pereira P.S., Siqueira-Junior J.P., Coutinho H.D.M., Quintans-Júnior L.J., da Silva T.G., Leal-Balbino T.C. (2018). Vitamin K enhances the effect of antibiotics inhibiting the efflux pumps of Staphylococcus aureus strains. Med. Chem. Res..

[35] Tu D., Blaha G., Moore P.B., Steitz T.A. (2005). Structures of MLSBK antibiotics bound to mutated large ribosomal subunits provide a structural explanation for resistance. Cell.

[36] Trott O., Olson A.J. (2010). AutoDock Vina: Improving the speed and accuracy of docking with a new scoring function, efficient optimization, and multithreading. J. Comput. Chem..

[37] Morris G.M., Goodsell D.S., Halliday R.S., Huey R., Hart W.E., Belew R.K., Olson A.J. (1998). Automated docking using a Lamarckian genetic algorithm and an empirical binding free energy function. J. Comput. Chem..

[38] Schrödinger L., DeLano W., Pymol (2020). http://www.pymol.org/pymol.

[39] Krieger E., Koraimann G., Vriend G. (2002). Increasing the precision of comparative models with YASARA NOVA—A self-parameterizing force field. Proteins.

[40] Duan Y., Wu C., Chowdhury S., Lee M.C., Xiong G., Zhang W., Yang R., Cieplak P., Luo R., Lee T. (2003). A point-charge force field for molecular mechanics simulations of proteins based on condensed-phase quantum mechanical calculations. J. Comput. Chem..

