# Supplementary material for: Design, Synthesis and Biological Evaluation of Conjugates of 3-O-Descladinose-azithromycin and Nucleobases against rRNA A2058G- or A2059G-Mutated Strains
Source: Molecules. 2023 Jan 30;28(3):1327. doi: 10.3390/molecules28031327 (PMC9920417; doi:10.3390/molecules28031327)
Supplement: Supplementary file 1 [file molecules-28-01327-s001.zip › molecules-2108821-SI.pdf]

Supplementary Materials for

Design, Synthesis and Biological Evaluation of Conjugates of 3-*O*-Descladinose-azithromycin and Nucleobases against rRNA A2058G or A2059G mutated Strains

Xiao-Tian Lian, [1, 2] Wen-Tian Liu, [1] Bing-Zhi Fan, [1]

Ming-Jia Yu [1, *] Jian-Hua Liang [1, 2, *]

1 Key Laboratory of Medical Molecule Science and Pharmaceutics Engineering, School of Chemistry and Chemical Engineering, Beijing Institute of Technology, Beijing 102488, China

2 Yangtze Delta Region Academy of Beijing Institute of Technology, Jiaxing 314019, China

Contents

1) Figure S1. The RMSD profiles of MD simulations of the G2099A mutant 50S ribosomal subunit with the ligands (**37A** and **32A**).
2) Figure S2. Superposition of two complexes including the last structures obtained from the MD and the complexes obtained from docking.
3) Table S1. The interaction of hydrogen bonds between G2099A Large Ribosomal Subunits (PDB ID: 1YHQ) and **37A**.
4) Table S2. The interaction of hydrogen bonds between G2099A mutant 50S ribosomal subunit (PDB ID: 1YHQ) and **32A**.
5) Table S3. The interaction of hydrogen bonds between G2099A mutant 50S ribosomal subunit (PDB ID: 1YHQ) and **37A** after molecular dynamic simulation.
6) Table S4. The interaction of hydrogen bonds between G2099A mutant 50S ribosomal subunit (PDB ID: 1YHQ) and **32A** after molecular dynamic simulation.
7) Table S5. HRMS and NMR spectra checklist for the intermediates and target compounds (**1 - 16** and **26 - 37**).
8) ^{1}H and ^{13}C NMR Spectra for the representative compounds.

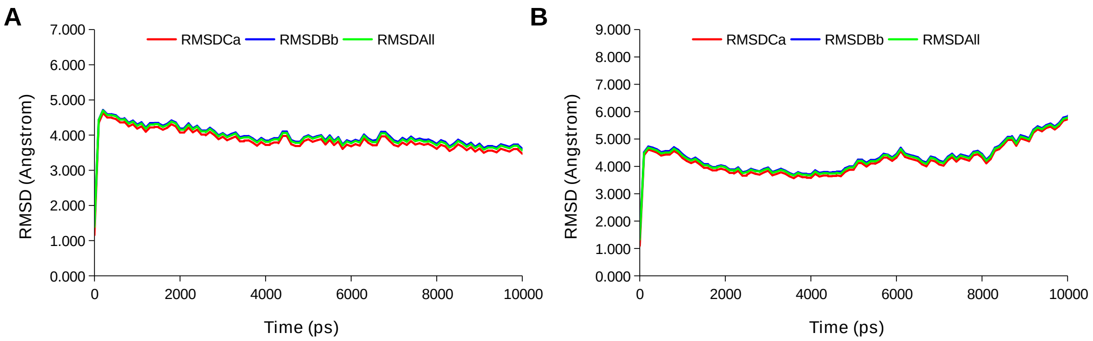

Figure S1. The RMSD profiles of MD simulations of the G2099A mutant 50S ribosomal subunit with the ligands (**37A** and **32A**). The RMSD values of complexes between A. G2099A mutant 50S ribosomal subunit and **37A** (40 ns), B. G2099A mutant 50S ribosomal subunit and **32A** (50 ns). The RMSD values of Calpha [RMSDCa], backbone [RMSDBb] and all-heavy atom [RMSDAll] are shown in red, blue and green, respectively.

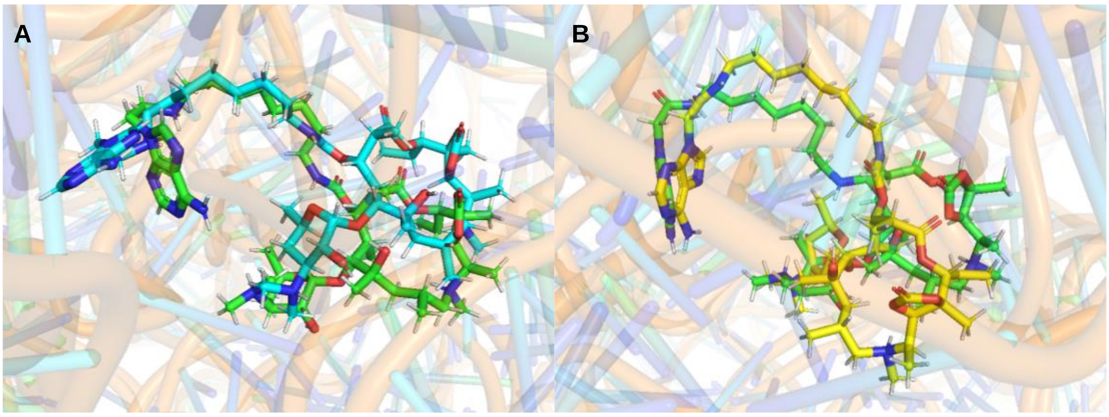

Figure S2. Superposition of two complexes including the last structures obtained from the MD and the complexes obtained from docking. The RMSD values of the structures aligned by PyMOL including the last structure from the MD and the complex obtained from docking are 2.971 and 2.802 respectively. A. G2099A mutant 50S ribosomal subunit with the ligand of **37A** (ligand of MD structure colored in cyan while that of docking structure colored in green). B. G2099A mutant 50S ribosomal subunit with the ligand of **32A** (ligand of MD structure colored in yellow while that of docking structure colored in green).

Table S1. The interaction of hydrogen bonds between G2099A Large Ribosomal Subunits (PDB ID: 1YHQ) and **37A**.

**Atoms in the Ribosomal Subunits**	**Atoms in the compound of 37A**	**Hydrogen bond (Å)**	**Hydrogen bond angle (°)**
A2096:HO2*	N5	2.96685	93.301
U2539:HO2*	N6	2.41225	137.64
G2611:H21	N4	2.85933	100.832
G2611:H22	N4	2.87959	99.593
U2539:O2*	H	2.42987	131.918
G2102:O6	HO	1.88273	139.413
G2540:O4*	HN	2.35251	133.353
U2541:O1P	H71	2.20592	138.283
U2541:O2P	H72	2.7853	107.691
G2540:O2*	H9	3.0211	131.659
C2487:O2	H14	2.97564	119.237
C2487:O2	H18	2.88956	124.92
C2487:O2	H262	2.72872	147.806
G2540:O3*	H271	2.51887	124.234
U2541:O1P	H271	2.85225	144.548
G2540:O2P	H321	2.87207	135.135
G2540:O4*	H321	3.04212	108.171
A2612:O2P	H391	2.97613	112.645
G2611:O1P	H42	2.67351	138.227
A2096:O2*	H43	3.07161	108.25
U2539:O3*	H43	2.91484	97.039
U2539:O2	H43	2.95632	127.691
A2095:O2P	H451	2.79739	123.175
G2097:O2P	H451	2.68124	130.17
G2094:O1P	H461	2.72497	130.008
A2095:O2P	H462	2.98838	121.184

Table S2. The interaction of hydrogen bonds between G2099A mutant 50S ribosomal subunit (PDB ID: 1YHQ) and **32A**.

Atoms in the Ribosomal Subunits	Atoms in the compound of 32A	Hydrogen bond (Å)	Hydrogen bond angle (°)
A2096:HO2*	N5	3.06553	93.523
G2540:O3*	H	2.95635	108.724
U2607:O2	HN81	2.71897	113.96
U2539:O2	HN82	2.3227	107.616
A2100:H2	O10	3.06658	119.984
A2538:H8	O3	2.76265	171.798
C2487:O2	H18	2.60408	157.811
G2540:O3*	H261	2.95574	125.148
U2541:O1P	H261	2.73889	157.804
C2487:O2	H262	2.81356	142.666
U2539:O2*	H263	2.7299	131.989
C2487:O2	H273	2.71451	145.599
C2104:O4*	H291	2.51104	159.424
G2540:O2P	H321	3.07242	129.665
G2097:O6	H391	3.08428	99.571
A2612:O2P	H391	2.4956	130.538
G2097:N7	H392	2.33957	156.451
G2611:O1P	H411	2.84204	126.264
G2097:O2P	H42	3.02797	119.662
U2541:O2P	H46	3.00114	119.815

Table S3. The interaction of hydrogen bonds between G2099A mutant 50S ribosomal subunit (PDB ID: 1YHQ) and **37A** after molecular dynamic simulation.

Atoms in the Ribosomal Subunits	Atoms in the compound of 37A	Hydrogen bond (Å)	Hydrogen bond angle (°)
G2540:O2P	HN81	2.3992	108.134
G2540:O1P	HN82	1.94309	145.085
A2099:H61	O1	2.93832	97.458
A2099:H62	O10	2.42	139.205
G2609:O3*	H71	2.52201	117.977
G2609:O2*	H71	2.10957	149.878
G2094:H5*1	N5	2.94857	102.83
A2100:H2	O10	2.63975	139.747
G2540:O2*	H14	2.74295	136.987
G2102:O6	H18	2.58355	131.962
C2487:O2	H262	2.49572	173.107
C2487:N3	H272	2.82287	142.596
G2102:O6	H273	2.26064	116.773
G2540:O1P	H42	2.70026	91.635
U2607:O4	H451	2.03383	131.991
A2095:O2P	H452	2.52185	124.369
A2096:O2*	H452	2.25123	126.036
U2607:O4	H462	2.86777	106.765

Table S4. The interaction of hydrogen bonds between G2099A mutant 50S ribosomal subunit (PDB ID: 1YHQ) and **32A** after molecular dynamic simulation.

Atoms in the Ribosomal Subunits	Atoms in the compound of 32A	Hydrogen bond (Å)	Hydrogen bond angle (°)
U2539:O1P	H	1.56196	171.248
G2102:H1	O7	2.06622	160.233
G2102:H21	O3	2.7009	141.187
C2104:H41	O7	2.65715	148.637
U2539:HO2*	N5	2.00121	145.234
G2611:H21	N7	2.58771	119.323
U2620:H3	O1	3.05445	93.8
U2541:O4*	HO	2.88069	110.223
C2104:O2	HN	1.91101	158.846
C2104:N3	H7	2.88319	115.454
U2539:O2	HN82	2.82441	111.826
U2539:H1*	N5	2.64062	134.268
G2540:H1*	O10	2.78106	147.738
C2104:O2	H2	2.67482	129.96
C2104:O2*	H10	2.1922	172.484
G2102:O6	H18	2.53202	157.698
U2539:O1P	H261	2.92808	97.267
A2486:N1	H262	2.92429	112.876
C2487:N3	H262	2.26448	139.975
G2102:O6	H263	2.64451	142.07
C2487:O2	H272	2.29241	144.62
U2539:O1P	H273	2.98835	90.525
A2486:N3	H282	2.26028	152.894
C2104:O2*	H291	2.18647	151.558
U2645:O2	H321	2.98063	117.358
U2645:O2	H322	3.07726	110.107
G2097:O6	H391	2.90565	120.308
G2540:O1P	H392	2.87747	122.421
G2540:O1P	H42	2.32524	154.78

Table S5. HMRS and NMR spectra checklist for the intermediates and target compounds.

Compounds	HRMS	^{1}H NMR	^{13}C NMR
**1**	√	√	
**3**	√	√	
**4**	√	√	
**5**		√	
**6**	√	√	
**7**		√	
**8**	√	√	
**9**		√	
**10**	√	√	
**12**		√	
**13**		√	
**14**	√	√	
**15**	√	√	
**16**	√	√	
**26T**	√	√	√
**28T**	√	√	√
**27U**	√	√	√
**28U**	√	√	√
**29U**	√	√	√
**30U**	√	√	√
**31U**	√	√	√
**32U**	√	√	√
**29A**	√	√	√
**30A**	√	√	√
**32A**	√	√	√
**33A**	√	√	√
**34A**	√	√	√
**35A**	√	√	√
**29G**	√	√	√
**30G**	√	√	√
**32G**	√	√	√
**29C**	√	√	√
**30C**	√	√	√
**36A**	√	√	
**37U**	√	√	√
**37A**	√	√	√

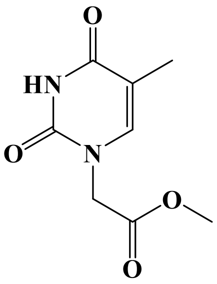

1

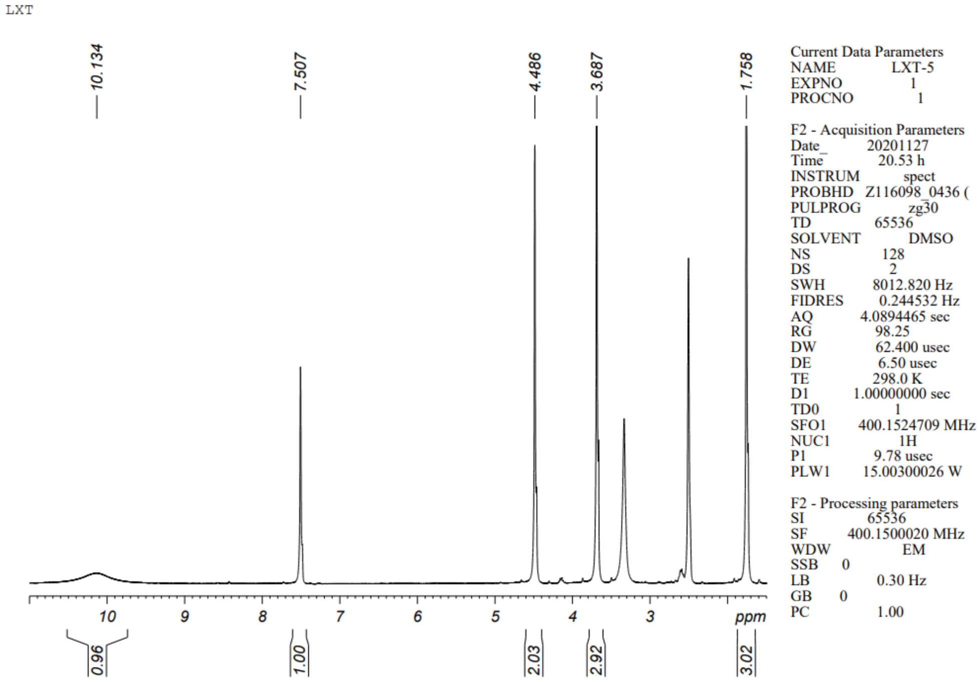
LXT
10.134
7.507
4.486
3.687
1.758
10
9
8
7
6
5
4
3
ppm
0.96
1.00
2.03
2.92
3.02
Current Data Parameters
NAME LXT-5
EXPNO 1
PROCNO 1
F2 - Acquisition Parameters
Date_ 20201127
Time 20.53 h
INSTRUM spect
PROBHD Z116098_0436 (
PULPROG zg30
TD 65536
SOLVENT DMSO
NS 128
DS 2
SWH 8012.820 Hz
FIDRES 0.244532 Hz
AQ 4.0894465 sec
RG 98.25
DW 62.400 usec
DE 6.50 usec
TE 298.0 K
D1 1.00000000 sec
TD0 1
SFO1 400.1524709 MHz
NUC1 1H
P1 9.78 usec
PLW1 15.00300026 W
F2 - Processing parameters
SI 65536
SF 400.1500020 MHz
WDW EM
SSB 0
LB 0.30 Hz
GB 0
PC 1.00

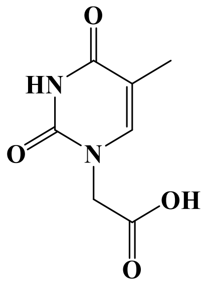

3

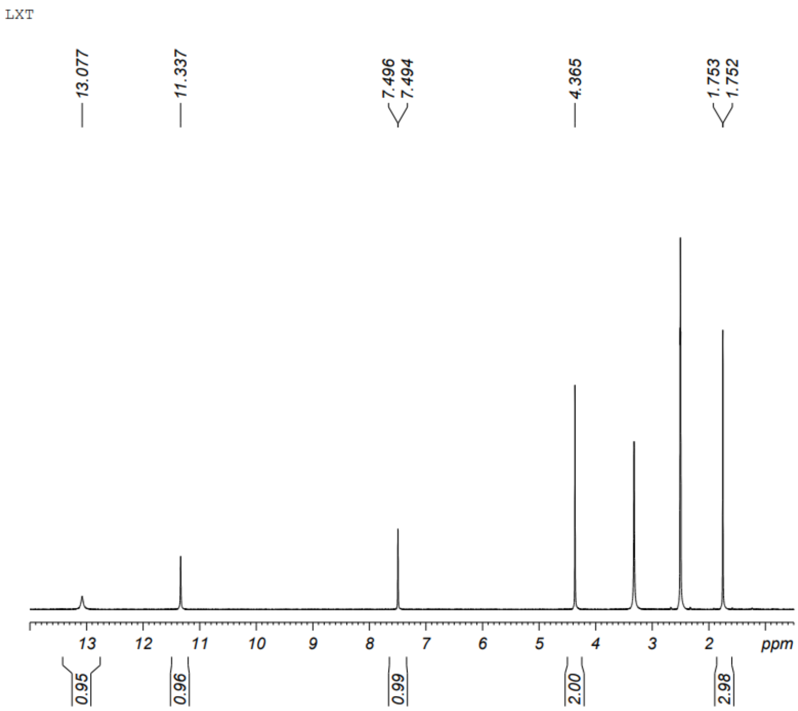

Current Data Parameters
NAME LXT-6
EXPNO 1
PROCNO 1

F2 - Acquisition Parameters
Date_ 20201211
Time 10.34 h
INSTRUM spect
PROBHD Z116098_0436 (
PULPROG zg30
TD 65536
SOLVENT DMSO
NS 16
DS 2
SWH 8012.820 Hz
FIDRES 0.244532 Hz
AQ 4.0894465 sec
RG 127.24
DW 62.400 usec
DE 6.50 usec
TE 298.0 K
D1 1.00000000 sec
TD0 1
SFO1 400.1524709 MHz
NUC1 1H
P1 9.78 usec
PLW1 15.00300026 W

F2 - Processing parameters
SI 65536
SF 400.1500026 MHz
WDW EM
SSB 0
LB 0.30 Hz
GB 0
PC 1.00

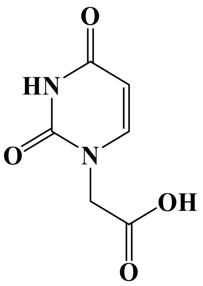
O
HN
O
N
OH
O

4

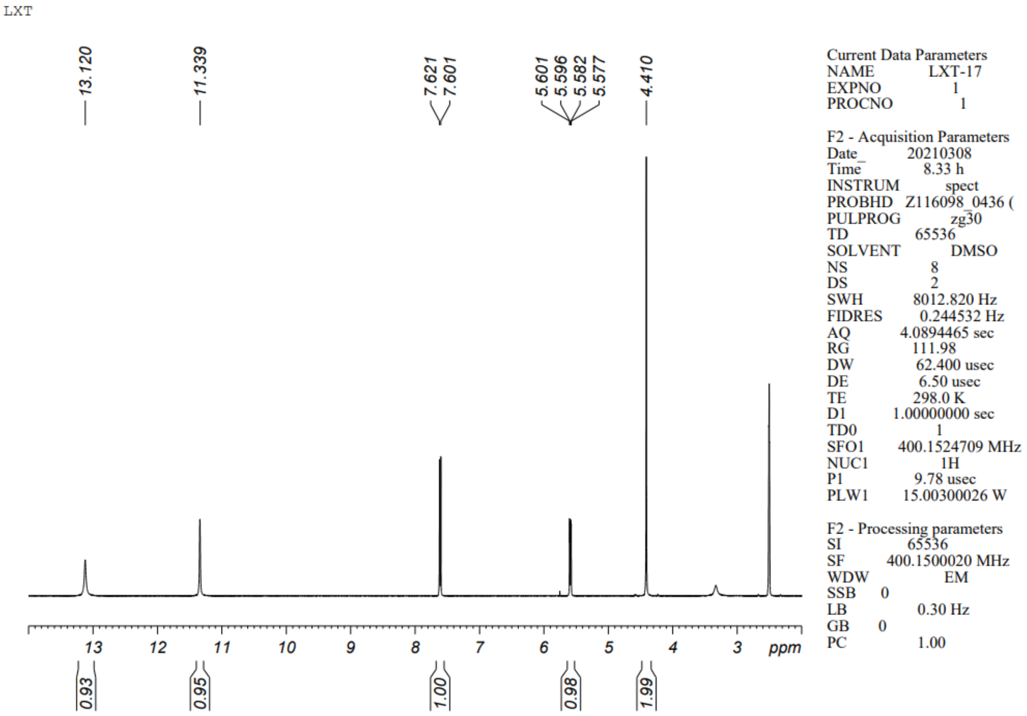
LXT
13.120
11.339
7.621
7.601
5.601
5.596
5.582
5.577
4.410
13
12
11
10
9
8
7
6
5
4
3
ppm
0.93
0.95
1.00
0.98
1.99
Current Data Parameters
NAME LXT-17
EXPNO 1
PROCNO 1
F2 - Acquisition Parameters
Date_ 20210308
Time 8.33 h
INSTRUM spect
PROBHD Z116098_0436 (
PULPROG zg30
TD 65536
SOLVENT DMSO
NS 8
DS 2
SWH 8012.820 Hz
FIDRES 0.244532 Hz
AQ 4.0894465 sec
RG 111.98
DW 62.400 usec
DE 6.50 usec
TE 298.0 K
D1 1.00000000 sec
TD0 1
SFO1 400.1524709 MHz
NUC1 1H
P1 9.78 usec
PLW1 15.00300026 W
F2 - Processing parameters
SI 65536
SF 400.1500020 MHz
WDW EM
SSB 0
LB 0.30 Hz
GB 0
PC 1.00

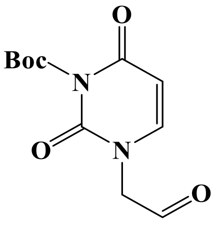

5

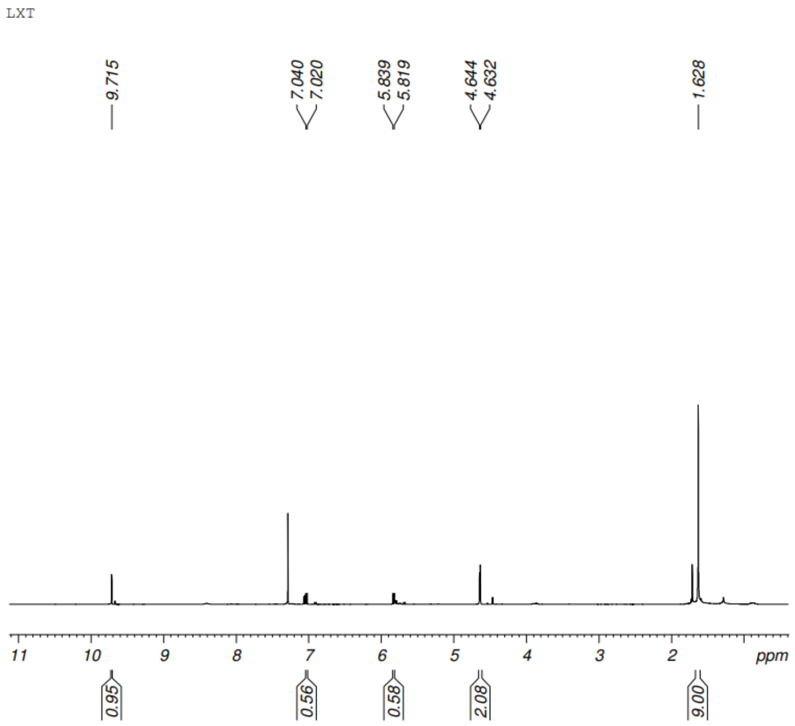

Current Data Parameters
NAME LXT-92
EXPNO 1
PROCNO 1

F2 - Acquisition Parameters
Date_ 20220505
Time 18.53 h
INSTRUM spect
PROBHD Z116098_0436 (
PULPROG zg30
TD 65536
SOLVENT CDCl3
NS 16
DS 2
SWH 8012.820 Hz
FIDRES 0.244532 Hz
AQ 4.0894465 sec
RG 143.44
DW 62.400 usec
DE 6.50 usec
TE 298.0 K
D1 1.00000000 sec
TD0 1
SFO1 400.1524709 MHz
NUC1 1H
P1 9.78 usec
PLW1 15.00300026 W

F2 - Processing parameters
SI 65536
SF 400.1500000 MHz
WDW EM
SSB 0
LB 0.30 Hz
GB 0
PC 1.00

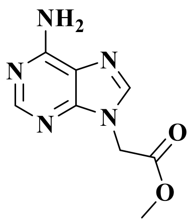

6

FBZ

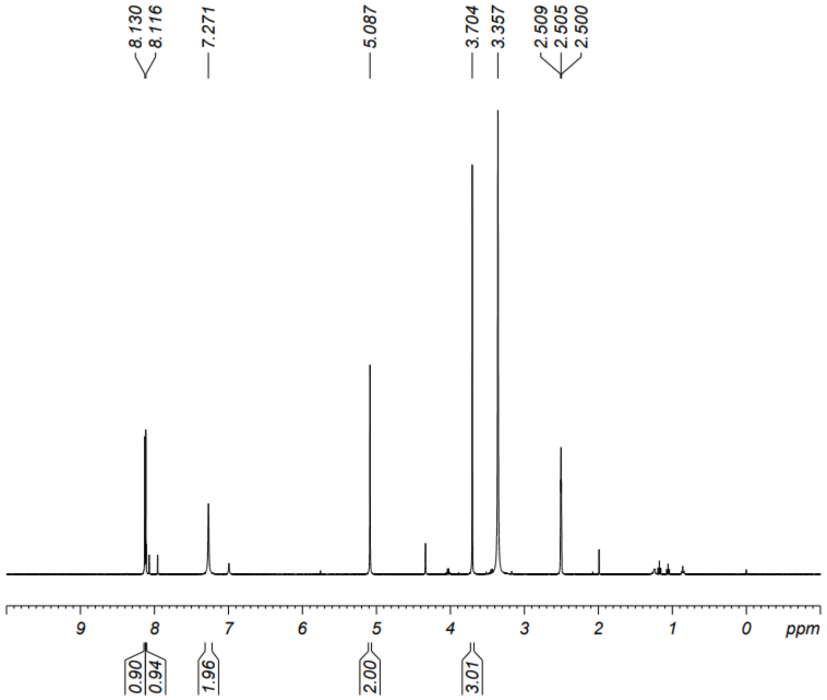

Current Data Parameters
NAME FBZ-77-1
EXPNO 1
PROCNO 1

F2 - Acquisition Parameters
Date_ 20190911
Time 16.10 h
INSTRUM spect
PROBHD Z116098_0436 (
PULPROG zg30
TD 65536
SOLVENT DMSO
NS 16
DS 2
SWH 8012.820 Hz
FIDRES 0.244532 Hz
AQ 4.0894465 sec
RG 98.25
DW 62.400 usec
DE 6.50 usec
TE 299.0 K
D1 1.00000000 sec
TD0 1
SFO1 400.1524709 MHz
NUC1 1H
P1 9.69 usec
PLW1 15.00300026 W

F2 - Processing parameters
SI 65536
SF 400.1500021 MHz
WDW EM
SSB 0
LB 0.30 Hz
GB 0
PC 1.00

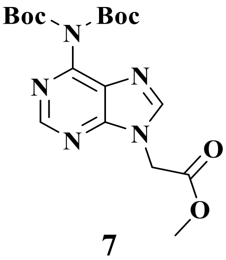

Boc
N
Boc
N
N
N
N
O
O

7

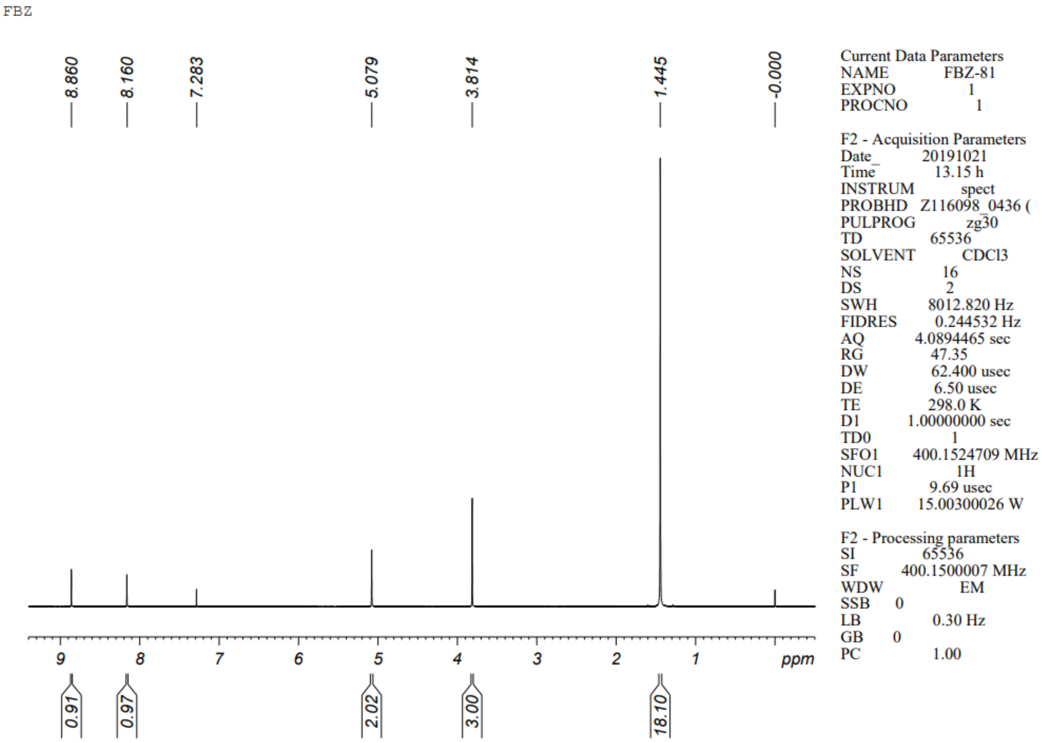

FBZ
8.860
8.160
7.283
5.079
3.814
1.445
-0.000
9
8
7
6
5
4
3
2
1
ppm
0.91
0.97
2.02
3.00
18.10
Current Data Parameters
NAME FBZ-81
EXPNO 1
PROCNO 1
F2 - Acquisition Parameters
Date_ 20191021
Time 13.15 h
INSTRUM spect
PROBHD Z116098_0436 (
PULPROG zg30
TD 65536
SOLVENT CDCl3
NS 16
DS 2
SWH 8012.820 Hz
FIDRES 0.244532 Hz
AQ 4.0894465 sec
RG 47.35
DW 62.400 usec
DE 6.50 usec
TE 298.0 K
D1 1.00000000 sec
TD0 1
SFO1 400.1524709 MHz
NUC1 1H
P1 9.69 usec
PLW1 15.00300026 W
F2 - Processing parameters
SI 65536
SF 400.1500007 MHz
WDW EM
SSB 0
LB 0.30 Hz
GB 0
PC 1.00

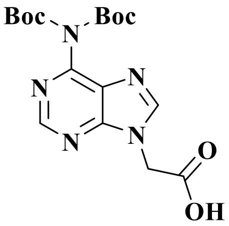

8

MCX

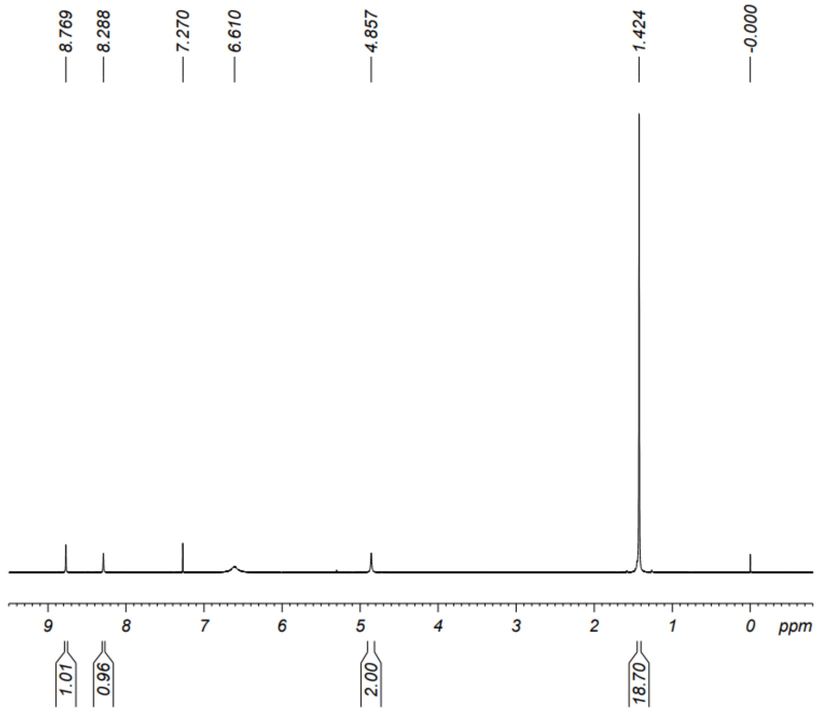

Current Data Parameters
NAME FBZ-82
EXPNO 1
PROCNO 1

F2 - Acquisition Parameters
Date_ 20191025
Time 4.00 h
INSTRUM spect
PROBHD Z116098_0436 (
PULPROG zg30
TD 65536
SOLVENT CDCl3
NS 16
DS 2
SWH 8012.820 Hz
FIDRES 0.244532 Hz
AQ 4.0894465 sec
RG 64.09
DW 62.400 usec
DE 6.50 usec
TE 297.8 K
D1 1.00000000 sec
TD0 1
SFO1 400.1524709 MHz
NUC1 1H
P1 9.69 usec
PLW1 15.00300026 W

F2 - Processing parameters
SI 65536
SF 400.1500062 MHz
WDW EM
SSB 0
LB 0.30 Hz
GB 0
PC 1.00

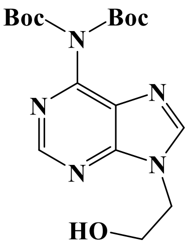

9

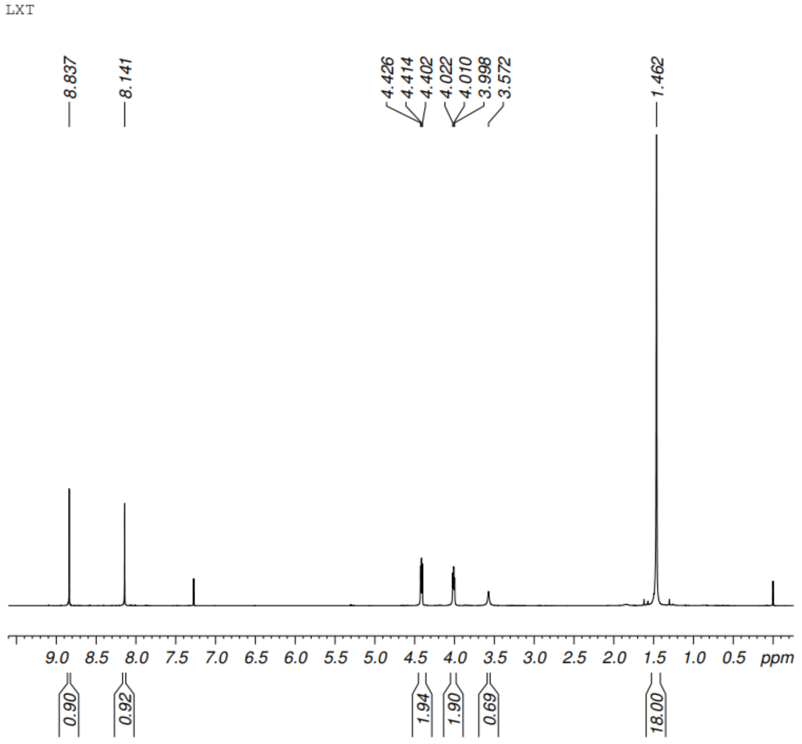

Current Data Parameters
NAME LXT-75
EXPNO 1
PROCNO 1

F2 - Acquisition Parameters
Date_ 20220221
Time 9.19 h
INSTRUM spect
PROBHD Z116098_0436 (
PULPROG zg30
TD 65536
SOLVENT CDCl3
NS 16
DS 2
SWH 8012.820 Hz
FIDRES 0.244532 Hz
AQ 4.0894465 sec
RG 64.09
DW 62.400 usec
DE 6.50 usec
TE 298.0 K
D1 1.00000000 sec
TD0 1
SFO1 400.1524709 MHz
NUC1 1H
P1 9.78 usec
PLW1 15.00300026 W

F2 - Processing parameters
SI 65536
SF 400.1500046 MHz
WDW EM
SSB 0
LB 0.30 Hz
GB 0
PC 1.00

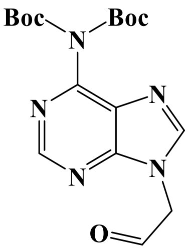

10

LXT

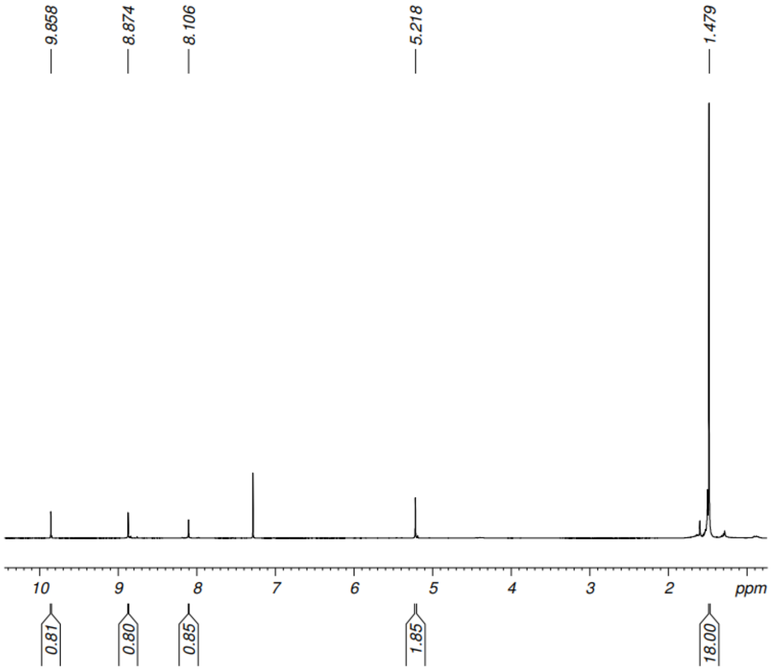

Current Data Parameters
NAME LXT-84-1
EXPNO 1
PROCNO 1

F2 - Acquisition Parameters
Date_ 20220506
Time 10.00 h
INSTRUM spect
PROBHD Z116098_0436 (
PULPROG zg30
TD 65536
SOLVENT CDCl3
NS 16
DS 2
SWH 8012.820 Hz
FIDRES 0.244532 Hz
AQ 4.0894465 sec
RG 127.24
DW 62.400 usec
DE 6.50 usec
TE 298.0 K
D1 1.00000000 sec
TD0 1
SFO1 400.1524709 MHz
NUC1 1H
P1 9.78 usec
PLW1 15.00300026 W

F2 - Processing parameters
SI 65536
SF 400.1500000 MHz
WDW EM
SSB 0
LB 0.30 Hz
GB 0
PC 1.00

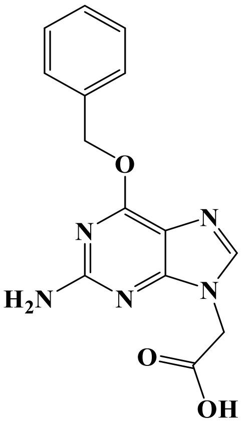

12

LXT,NS=32

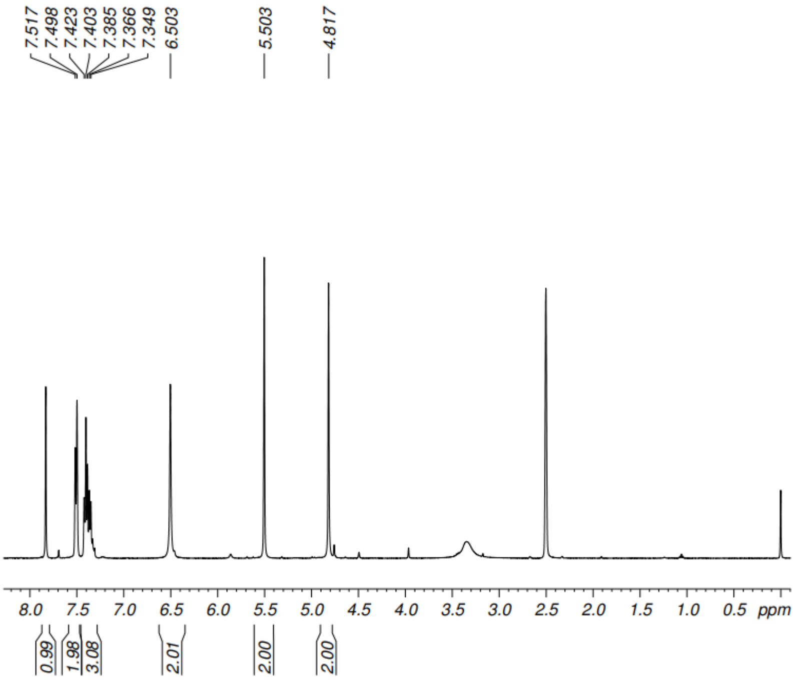

Current Data Parameters
NAME LXT-29-1
EXPNO 1
PROCNO 1

F2 - Acquisition Parameters
Date_ 20210517
Time 8.18 h
INSTRUM spect
PROBHD Z116098_0436 (
PULPROG zg30
TD 65536
SOLVENT DMSO
NS 32
DS 2
SWH 8012.820 Hz
FIDRES 0.244532 Hz
AQ 4.0894465 sec
RG 127.24
DW 62.400 usec
DE 6.50 usec
TE 298.0 K
D1 1.00000000 sec
TD0 1
SFO1 400.1524709 MHz
NUC1 1H
P1 9.78 usec
PLW1 15.00300026 W

F2 - Processing parameters
SI 65536
SF 400.1500030 MHz
WDW EM
SSB 0
LB 0.30 Hz
GB 0
PC 1.00

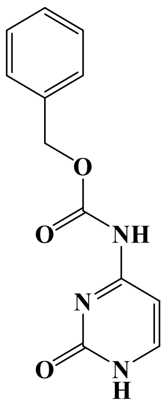

13

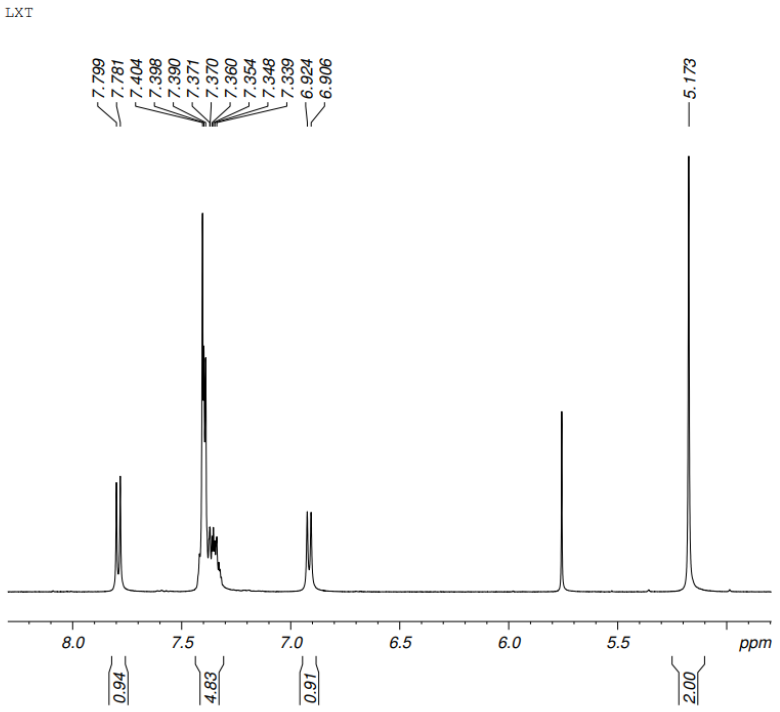

Current Data Parameters
NAME LXT-11-1
EXPNO 1
PROCNO 1

F2 - Acquisition Parameters
Date_ 20210324
Time 9.40 h
INSTRUM spect
PROBHD Z116098_0436 (
PULPROG zg30
TD 65536
SOLVENT DMSO
NS 32
DS 2
SWH 8012.820 Hz
FIDRES 0.244532 Hz
AQ 4.0894465 sec
RG 127.24
DW 62.400 usec
DE 6.50 usec
TE 298.0 K
D1 1.00000000 sec
TD0 1
SFO1 400.1524709 MHz
NUC1 1H
P1 9.78 usec
PLW1 15.00300026 W

F2 - Processing parameters
SI 65536
SF 400.1500029 MHz
WDW EM
SSB 0
LB 0.30 Hz
GB 0
PC 1.00

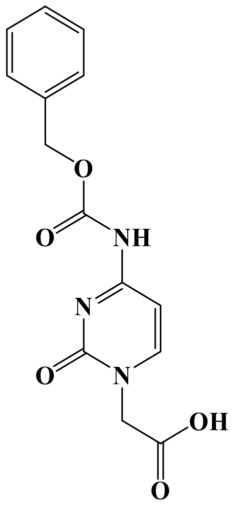
O
O
NH
N
O
N
OH
O

14

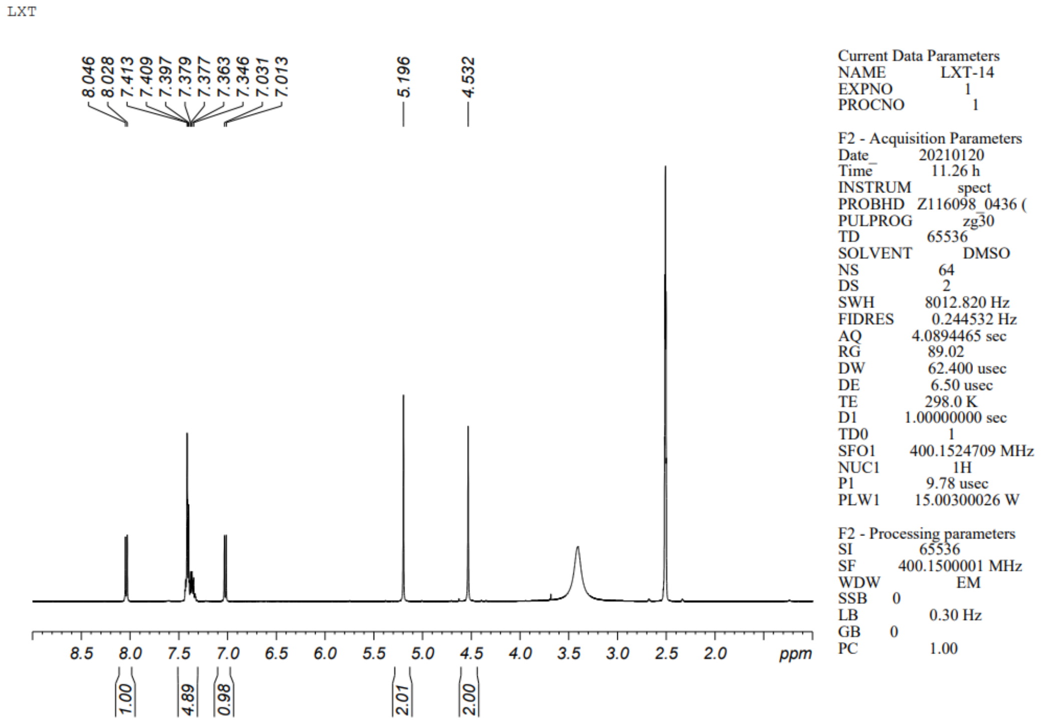
LXT
8.046
8.028
7.413
7.409
7.397
7.379
7.377
7.363
7.346
7.031
7.013
5.196
4.532
8.5 8.0 7.5 7.0 6.5 6.0 5.5 5.0 4.5 4.0 3.5 3.0 2.5 2.0 ppm
1.00
4.89
0.98
2.01
2.00
Current Data Parameters
NAME LXT-14
EXPNO 1
PROCNO 1
F2 - Acquisition Parameters
Date_ 20210120
Time 11.26 h
INSTRUM spect
PROBHD Z116098_0436 (
PULPROG zg30
TD 65536
SOLVENT DMSO
NS 64
DS 2
SWH 8012.820 Hz
FIDRES 0.244532 Hz
AQ 4.0894465 sec
RG 89.02
DW 62.400 usec
DE 6.50 usec
TE 298.0 K
D1 1.00000000 sec
TD0 1
SFO1 400.1524709 MHz
NUC1 1H
P1 9.78 usec
PLW1 15.00300026 W
F2 - Processing parameters
SI 65536
SF 400.1500001 MHz
WDW EM
SSB 0
LB 0.30 Hz
GB 0
PC 1.00

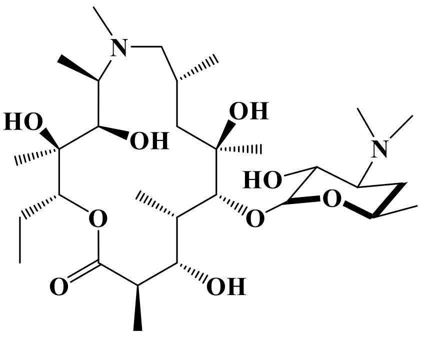
HO
OH
OH
N
N
HO
O
O
O
O
OH

15

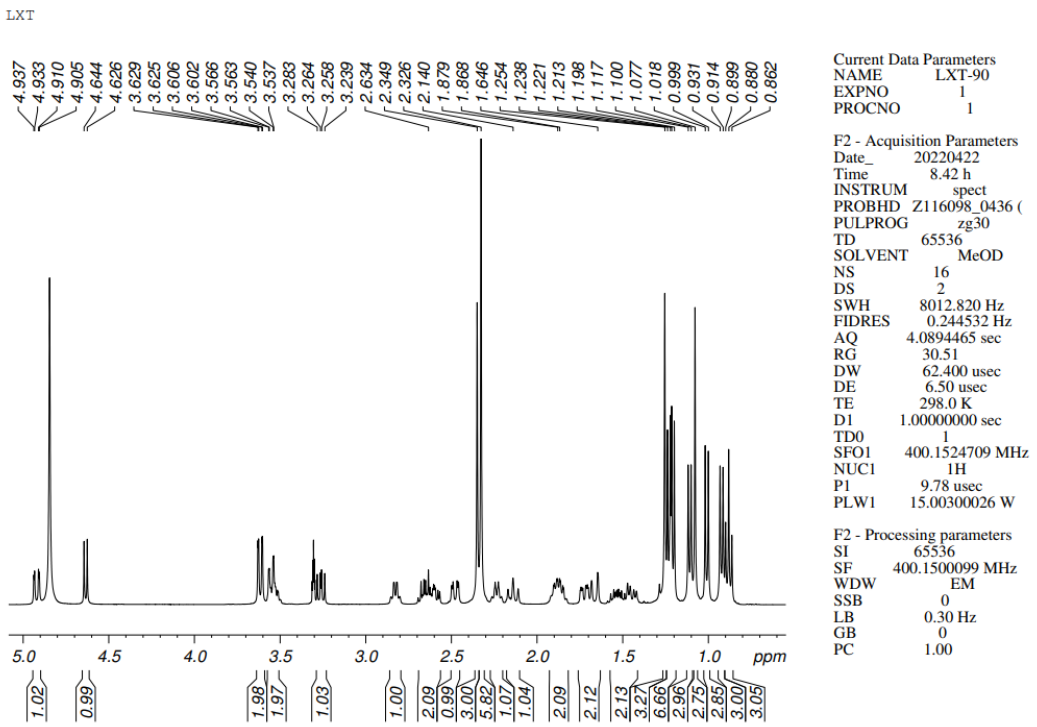
LXT
4.937 4.933 4.910 4.905 4.644 4.626 3.629 3.625 3.606 3.602 3.566 3.563 3.540 3.537 3.283 3.264 3.258 3.239 2.634 2.349 2.326 2.140 1.879 1.868 1.646 1.254 1.238 1.221 1.213 1.198 1.117 1.100 1.077 1.018 0.999 0.931 0.914 0.899 0.880 0.862
5.0 4.5 4.0 3.5 3.0 2.5 2.0 1.5 1.0 ppm
1.02 0.99 1.98 1.97 1.03 1.00 2.09 0.99 3.00 5.82 1.07 1.04 2.09 2.12 2.13 3.27 6.66 2.96 2.75 2.85 3.00 3.05
Current Data Parameters
NAME LXT-90
EXPNO 1
PROCNO 1
F2 - Acquisition Parameters
Date_ 20220422
Time 8.42 h
INSTRUM spect
PROBHD Z116098_0436 (
PULPROG zg30
TD 65536
SOLVENT MeOD
NS 16
DS 2
SWH 8012.820 Hz
FIDRES 0.244532 Hz
AQ 4.0894465 sec
RG 30.51
DW 62.400 usec
DE 6.50 usec
TE 298.0 K
D1 1.00000000 sec
TD0 1
SFO1 400.1524709 MHz
NUC1 1H
P1 9.78 usec
PLW1 15.00300026 W
F2 - Processing parameters
SI 65536
SF 400.1500099 MHz
WDW EM
SSB 0
LB 0.30 Hz
GB 0
PC 1.00

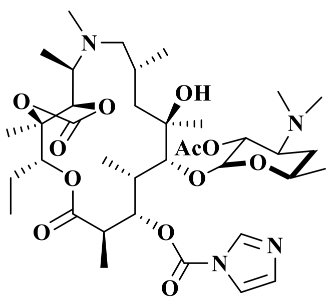

16

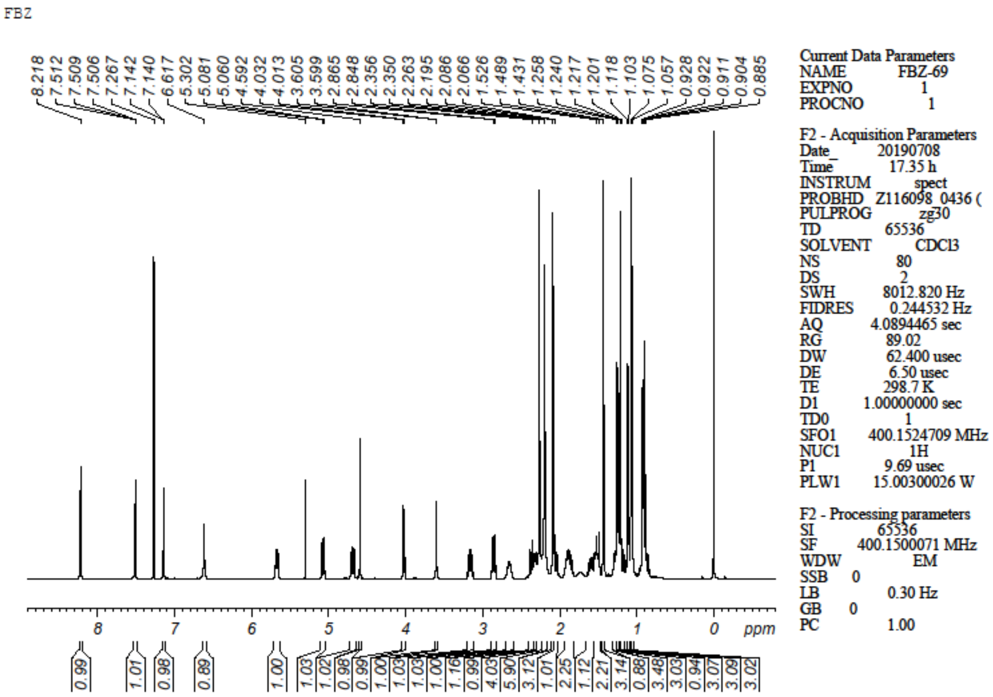
FBZ
8.218 7.512 7.509 7.506 7.267 7.142 7.140 6.617 5.302 5.081 5.060 4.592 4.032 4.013 3.605 3.599 2.865 2.848 2.356 2.350 2.263 2.195 2.086 2.066 1.526 1.489 1.431 1.258 1.240 1.217 1.201 1.118 1.103 1.075 1.057 0.928 0.922 0.911 0.904 0.885
8 7 6 5 4 3 2 1 0 ppm
Current Data Parameters
NAME FBZ-69
EXPNO 1
PROCNO 1
F2 - Acquisition Parameters
Date_ 20190708
Time 17.35 h
INSTRUM spect
PROBHD Z116098_0436 (
PULPROG zg30
TD 65536
SOLVENT CDCl3
NS 80
DS 2
SWH 8012.820 Hz
FIDRES 0.244532 Hz
AQ 4.0894465 sec
RG 89.02
DW 62.400 usec
DE 6.50 usec
TE 298.7 K
D1 1.00000000 sec
TD0 1
SFO1 400.1524709 MHz
NUC1 1H
P1 9.69 usec
PLW1 15.00300026 W
F2 - Processing parameters
SI 65536
SF 400.1500071 MHz
WDW EM
SSB 0
LB 0.30 Hz
GB 0
PC 1.00

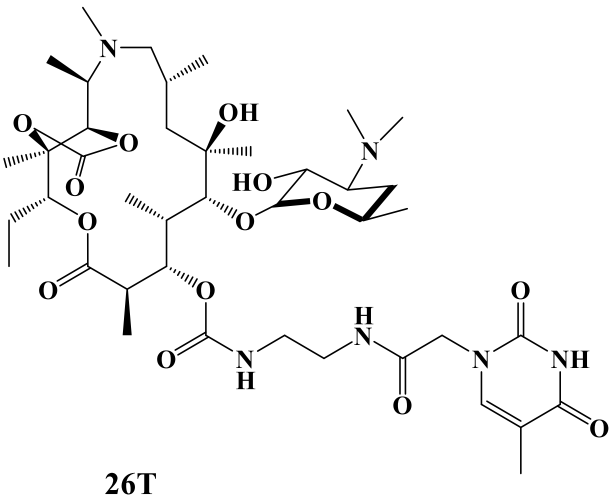
OH
HO
O
N
NH
H
O

26T

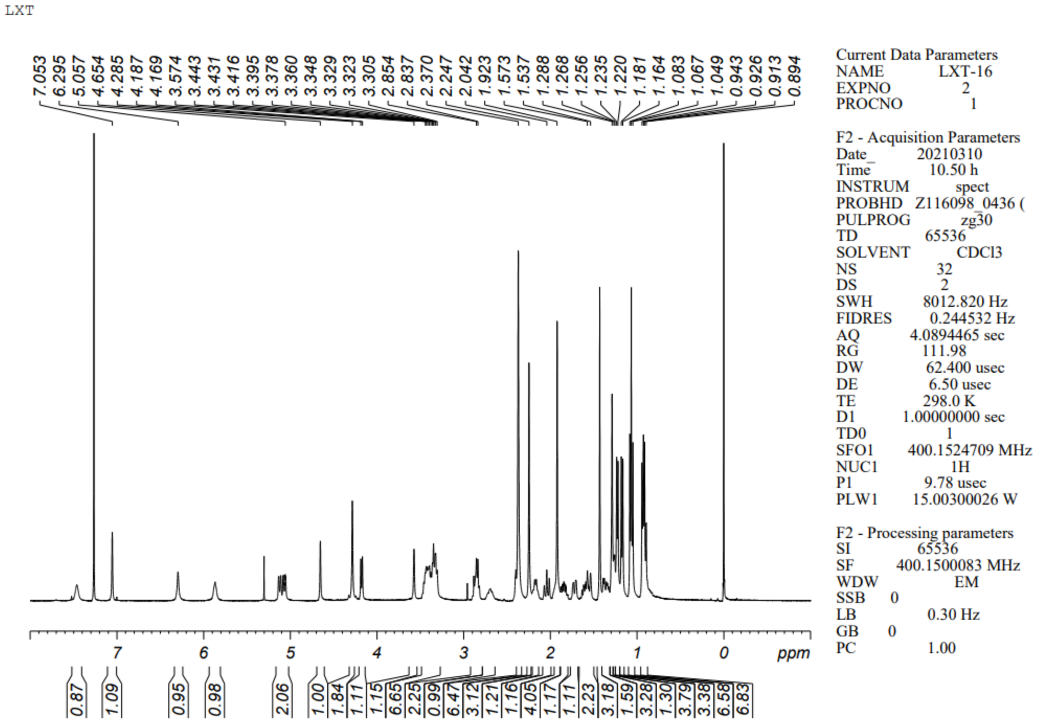
LXT
7.053 6.295 5.057 4.654 4.285 4.187 4.169 3.574 3.443 3.431 3.416 3.395 3.378 3.360 3.348 3.329 3.323 3.305 2.854 2.837 2.370 2.247 2.042 1.923 1.573 1.537 1.288 1.268 1.256 1.235 1.220 1.181 1.164 1.083 1.067 1.049 0.943 0.926 0.913 0.894
Current Data Parameters
NAME LXT-16
EXPNO 2
PROCNO 1
F2 - Acquisition Parameters
Date_ 20210310
Time 10.50 h
INSTRUM spect
PROBHD Z116098_0436 (
PULPROG zg30
TD 65536
SOLVENT CDCl3
NS 32
DS 2
SWH 8012.820 Hz
FIDRES 0.244532 Hz
AQ 4.0894465 sec
RG 111.98
DW 62.400 usec
DE 6.50 usec
TE 298.0 K
D1 1.00000000 sec
TD0 1
SFO1 400.1524709 MHz
NUC1 1H
P1 9.78 usec
PLW1 15.00300026 W
F2 - Processing parameters
SI 65536
SF 400.1500083 MHz
WDW EM
SSB 0
LB 0.30 Hz
GB 0
PC 1.00
7 6 5 4 3 2 1 0 ppm
0.87 1.09 0.95 0.98 2.06 1.00 1.84 1.11 1.15 6.65 2.25 0.99 6.47 3.12 1.21 1.16 4.05 1.17 1.11 2.23 3.18 1.59 3.28 1.30 3.79 3.38 6.58 6.83

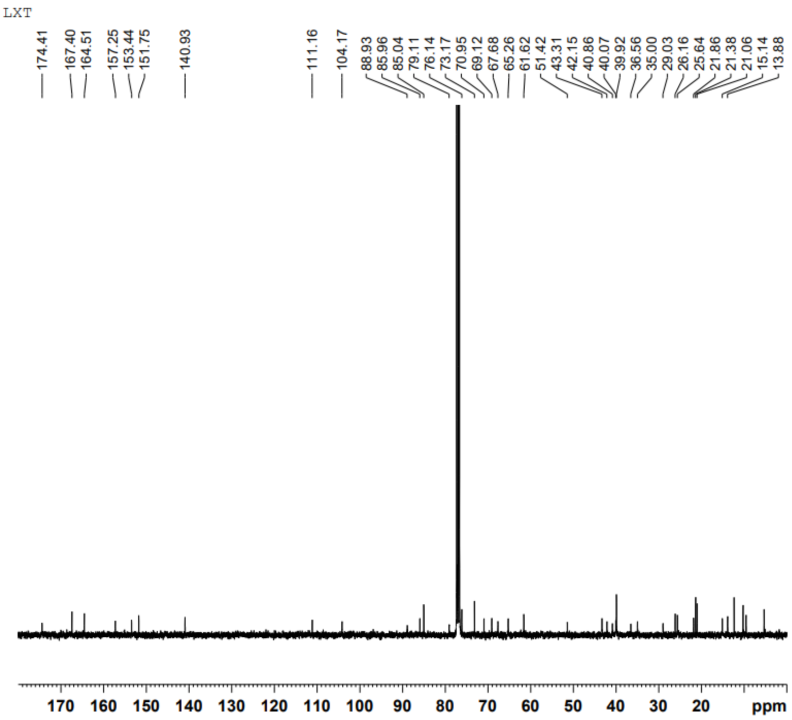

Current Data Parameters
NAME LXT-16
EXPNO 3
PROCNO 1

F2 - Acquisition Parameters
Date_ 20210312
Time 21.41 h
INSTRUM spect
PROBHD Z116098_0436 (
PULPROG zgpg30
TD 65536
SOLVENT CDCl3
NS 1024
DS 4
SWH 24038.461 Hz
FIDRES 0.733596 Hz
AQ 1.3631488 sec
RG 202.1
DW 20.800 usec
DE 6.50 usec
TE 298.0 K
D1 2.00000000 sec
D11 0.03000000 sec
TD0 1
SFO1 100.6278593 MHz
NUC1 13C
P1 10.58 usec
PLW1 68.07700348 W
SFO2 400.1516006 MHz
NUC2 1H
CPDPRG[2 waltz16
PCPD2 80.00 usec
PLW2 15.00300026 W
PLW12 0.22421999 W
PLW13 0.11260000 W

F2 - Processing parameters
SI 32768
SF 100.6177975 MHz
WDW EM
SSB 0
LB 1.00 Hz
GB 0
PC 1.40

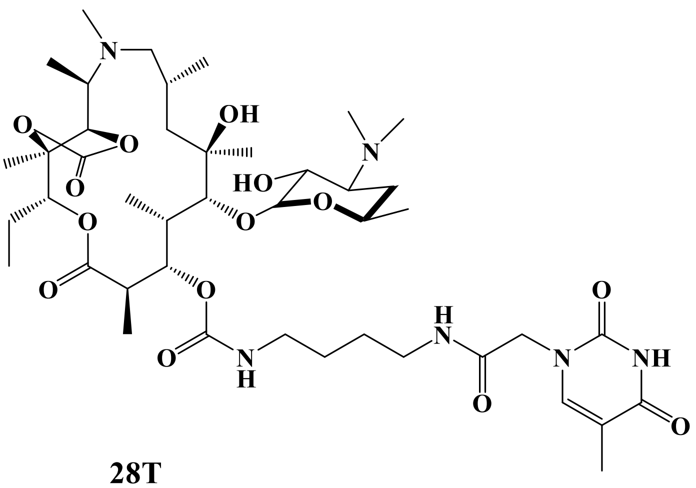

28T

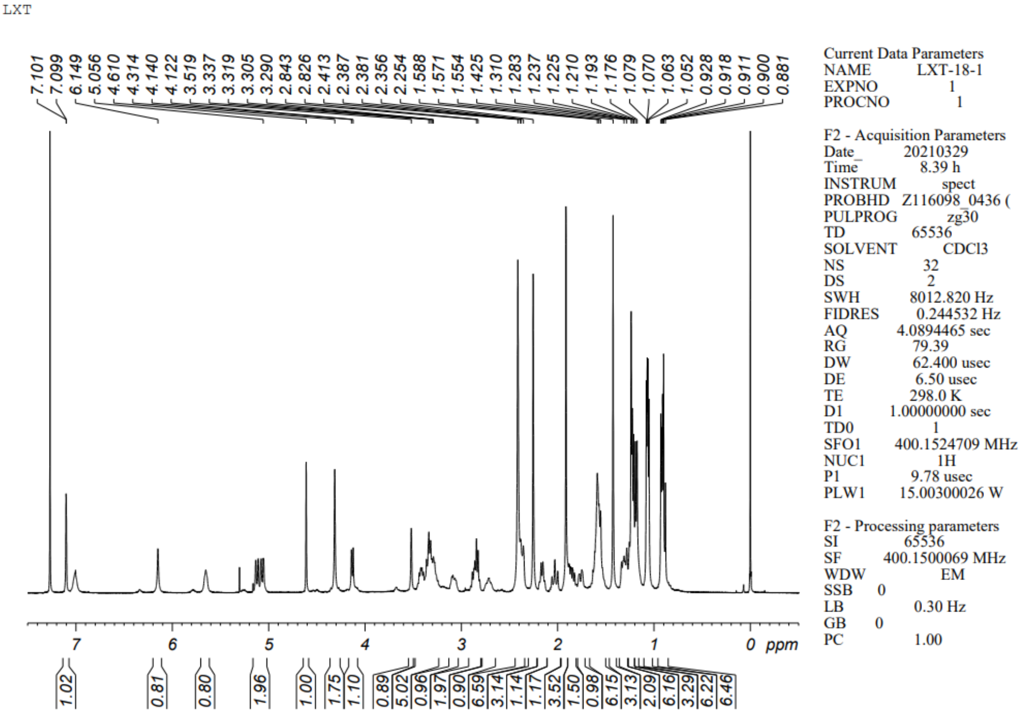
LXT
7.101
7.099
6.149
5.056
4.610
4.314
4.140
4.122
3.519
3.337
3.319
3.305
3.290
2.843
2.826
2.413
2.387
2.381
2.356
2.254
1.588
1.571
1.554
1.425
1.310
1.283
1.237
1.225
1.210
1.193
1.176
1.079
1.070
1.063
1.052
0.928
0.918
0.911
0.900
0.881
7 6 5 4 3 2 1 0 ppm
1.02
0.81
0.80
1.96
1.00
1.75
1.10
0.89
5.02
0.96
1.97
0.90
6.59
3.14
1.14
1.17
3.52
1.50
0.98
6.15
3.13
2.09
6.16
3.29
6.22
6.46
Current Data Parameters
NAME LXT-18-1
EXPNO 1
PROCNO 1
F2 - Acquisition Parameters
Date_ 20210329
Time 8.39 h
INSTRUM spect
PROBHD Z116098_0436 (
PULPROG zg30
TD 65536
SOLVENT CDCl3
NS 32
DS 2
SWH 8012.820 Hz
FIDRES 0.244532 Hz
AQ 4.0894465 sec
RG 79.39
DW 62.400 usec
DE 6.50 usec
TE 298.0 K
D1 1.00000000 sec
TD0 1
SFO1 400.1524709 MHz
NUC1 1H
P1 9.78 usec
PLW1 15.00300026 W
F2 - Processing parameters
SI 65536
SF 400.1500069 MHz
WDW EM
SSB 0
LB 0.30 Hz
GB 0
PC 1.00

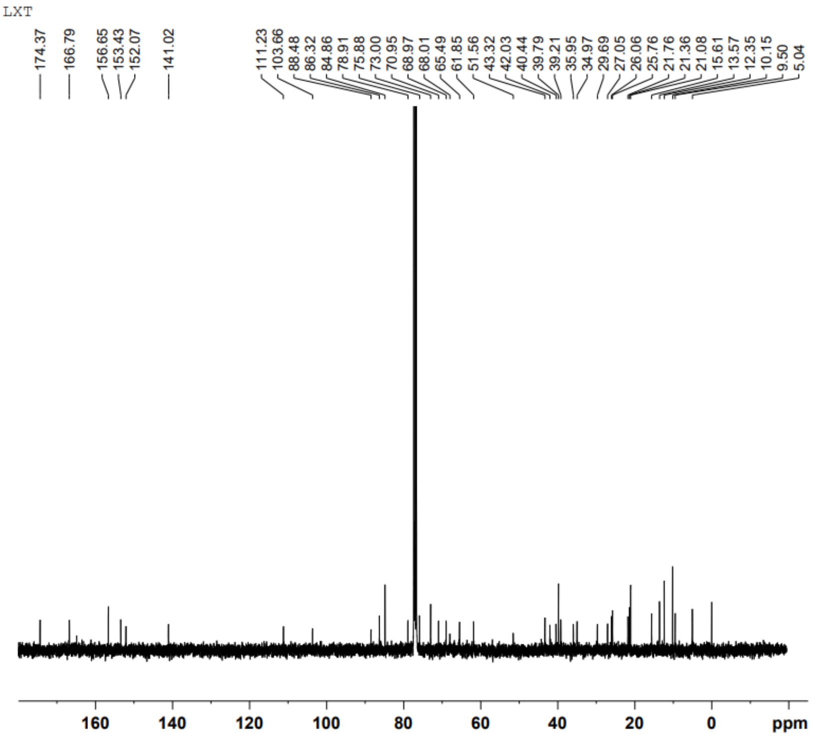

Current Data Parameters
NAME LXT-18-1
EXPNO 3
PROCNO 1

F2 - Acquisition Parameters
Date_ 20210330
Time 23.43 h
INSTRUM spect
PROBHD Z116098_0436 (
PULPROG zgpg30
TD 65536
SOLVENT CDCl3
NS 1024
DS 4
SWH 24038.461 Hz
FIDRES 0.733596 Hz
AQ 1.3631488 sec
RG 202.1
DW 20.800 usec
DE 6.50 usec
TE 298.0 K
D1 2.00000000 sec
D11 0.03000000 sec
TD0 1
SFO1 100.6278593 MHz
NUC1 13C
P1 10.58 usec
PLW1 68.07700348 W
SFO2 400.1516006 MHz
NUC2 1H
CPDPRG[2 waltz16
PCPD2 80.00 usec
PLW2 15.00300026 W
PLW12 0.22421999 W
PLW13 0.11260000 W

F2 - Processing parameters
SI 32768
SF 100.6177979 MHz
WDW EM
SSB 0
LB 1.00 Hz
GB 0
PC 1.40

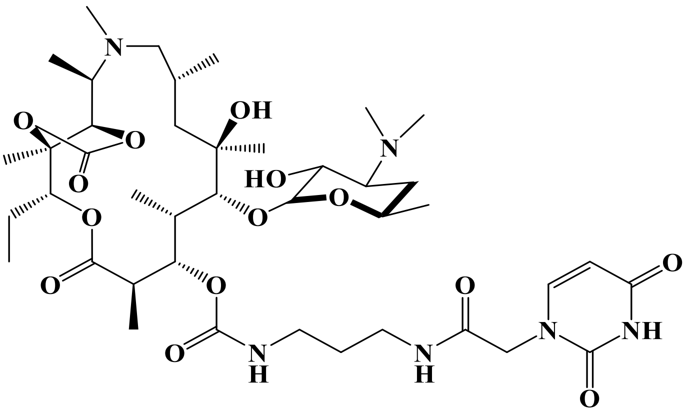

OH
HO
N
O
NH
H

27U

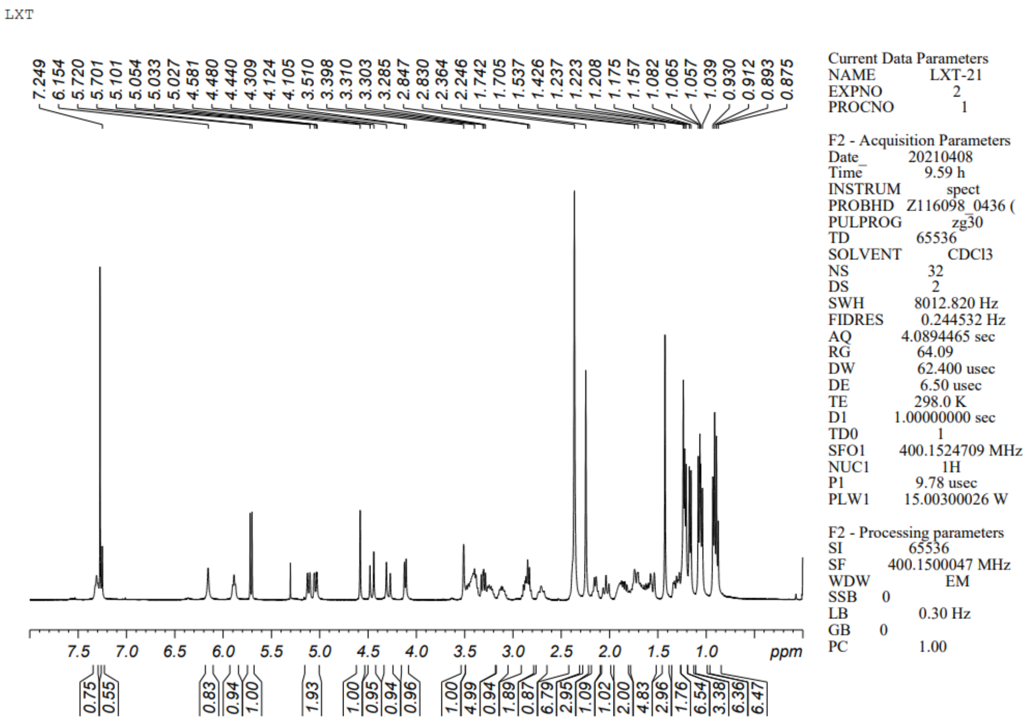

LXT
Current Data Parameters
NAME LXT-21
EXPNO 2
PROCNO 1
F2 - Acquisition Parameters
Date_ 20210408
Time 9.59 h
INSTRUM spect
PROBHD Z116098_0436 (
PULPROG zg30
TD 65536
SOLVENT CDCl3
NS 32
DS 2
SWH 8012.820 Hz
FIDRES 0.244532 Hz
AQ 4.0894465 sec
RG 64.09
DW 62.400 usec
DE 6.50 usec
TE 298.0 K
D1 1.00000000 sec
TD0 1
SFO1 400.1524709 MHz
NUC1 1H
P1 9.78 usec
PLW1 15.00300026 W
F2 - Processing parameters
SI 65536
SF 400.1500047 MHz
WDW EM
SSB 0
LB 0.30 Hz
GB 0
PC 1.00
ppm

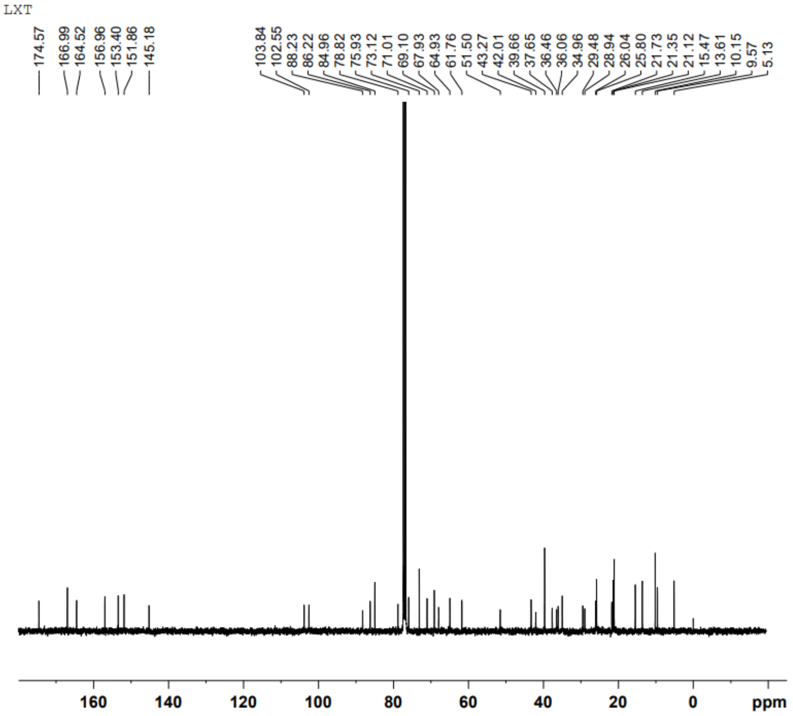

Current Data Parameters
NAME LXT-21
EXPNO 3
PROCNO 1

F2 - Acquisition Parameters
Date_ 20210409
Time 4.42 h
INSTRUM spect
PROBHD Z116098_0436 (
PULPROG zgpg30
TD 65536
SOLVENT CDCl3
NS 1024
DS 4
SWH 24038.461 Hz
FIDRES 0.733596 Hz
AQ 1.3631488 sec
RG 202.1
DW 20.800 usec
DE 6.50 usec
TE 298.0 K
D1 2.00000000 sec
D11 0.03000000 sec
TD0 1
SFO1 100.6278593 MHz
NUC1 13C
P1 10.58 usec
PLW1 68.07700348 W
SFO2 400.1516006 MHz
NUC2 1H
CPDPRG[2 waltz16
PCPD2 80.00 usec
PLW2 15.00300026 W
PLW12 0.22421999 W
PLW13 0.11260000 W

F2 - Processing parameters
SI 32768
SF 100.6177975 MHz
WDW EM
SSB 0
LB 1.00 Hz
GB 0
PC 1.40

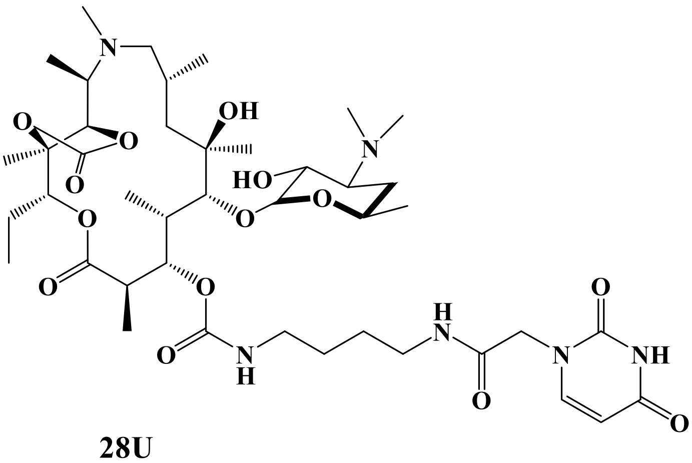

28U

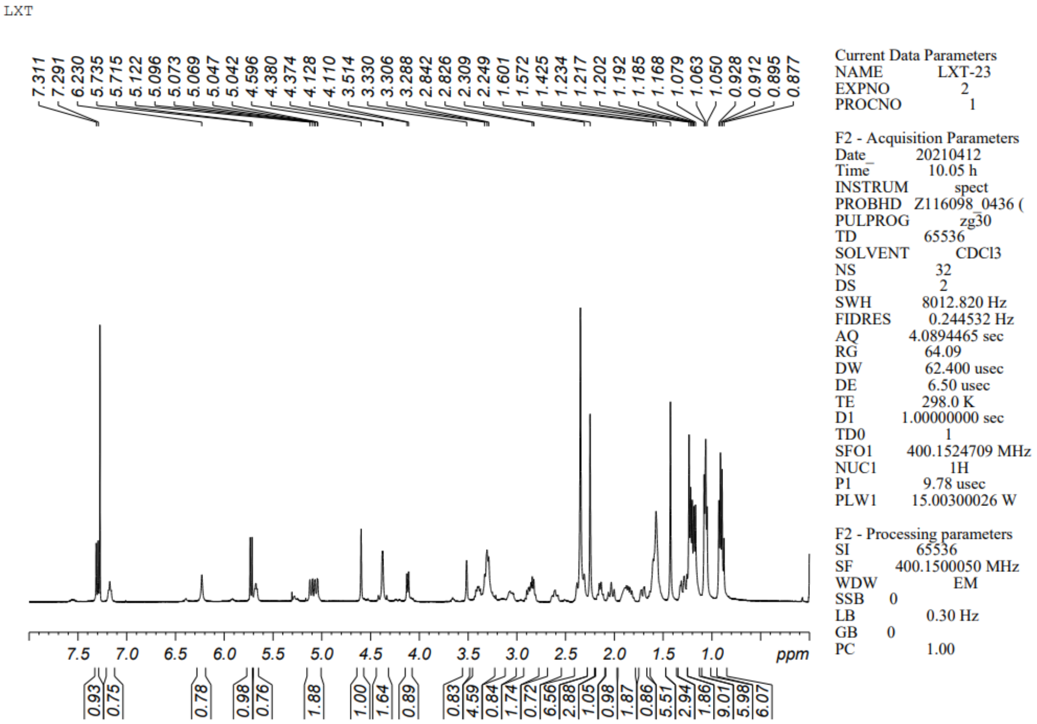
LXT
Current Data Parameters
NAME LXT-23
EXPNO 2
PROCNO 1
F2 - Acquisition Parameters
Date_ 20210412
Time 10.05 h
INSTRUM spect
PROBHD Z116098_0436 (
PULPROG zg30
TD 65536
SOLVENT CDCl3
NS 32
DS 2
SWH 8012.820 Hz
FIDRES 0.244532 Hz
AQ 4.0894465 sec
RG 64.09
DW 62.400 usec
DE 6.50 usec
TE 298.0 K
D1 1.00000000 sec
TD0 1
SFO1 400.1524709 MHz
NUC1 1H
P1 9.78 usec
PLW1 15.00300026 W
F2 - Processing parameters
SI 65536
SF 400.1500050 MHz
WDW EM
SSB 0
LB 0.30 Hz
GB 0
PC 1.00
ppm

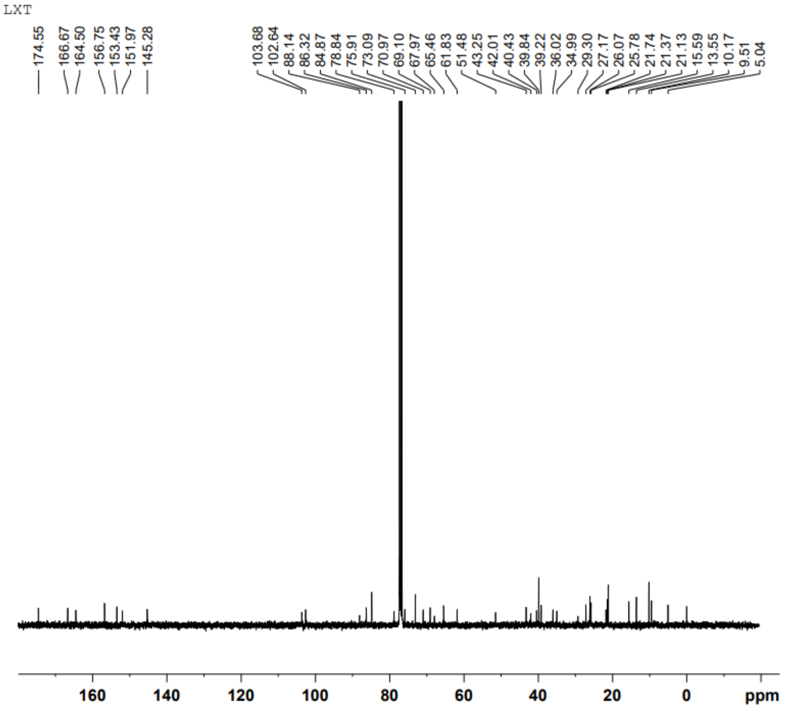

Current Data Parameters
NAME LXT-23
EXPNO 3
PROCNO 1

F2 - Acquisition Parameters
Date_ 20210413
Time 4.08 h
INSTRUM spect
PROBHD Z116098_0436 (
PULPROG zgpg30
TD 65536
SOLVENT CDCl3
NS 1024
DS 4
SWH 24038.461 Hz
FIDRES 0.733596 Hz
AQ 1.3631488 sec
RG 202.1
DW 20.800 usec
DE 6.50 usec
TE 298.0 K
D1 2.00000000 sec
D11 0.03000000 sec
TD0 1
SFO1 100.6278593 MHz
NUC1 13C
P1 10.58 usec
PLW1 68.07700348 W
SFO2 400.1516006 MHz
NUC2 1H
CPDPRG[2 waltz16
PCPD2 80.00 usec
PLW2 15.00300026 W
PLW12 0.22421999 W
PLW13 0.11260000 W

F2 - Processing parameters
SI 32768
SF 100.6177975 MHz
WDW EM
SSB 0
LB 1.00 Hz
GB 0
PC 1.40

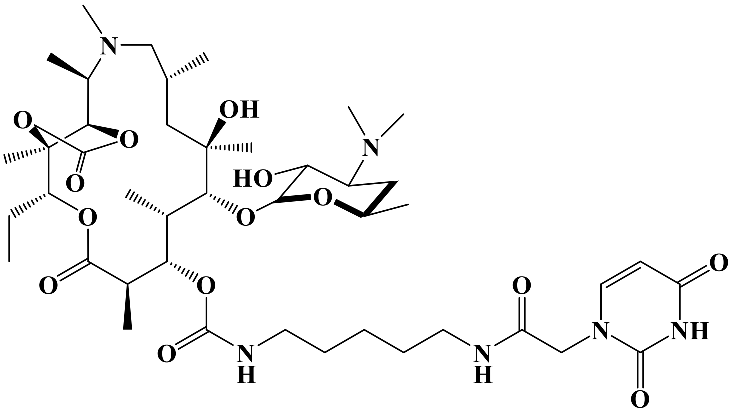
OH
HO
O
N
NH
H

29U

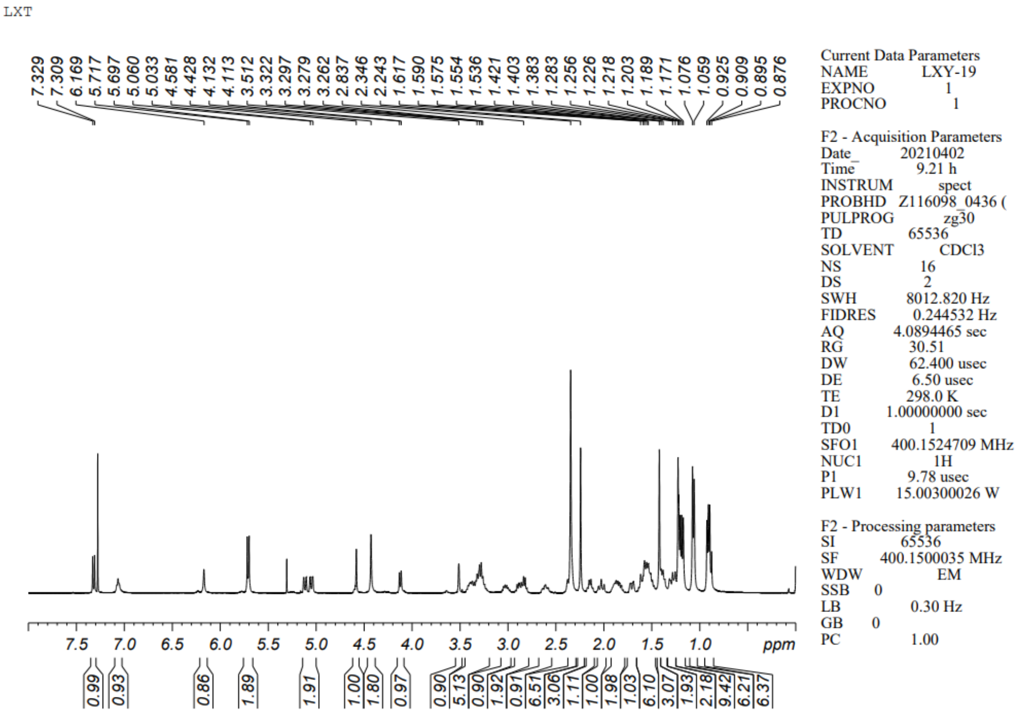
LXT
7.329
7.309
6.169
5.717
5.697
5.060
5.033
4.581
4.428
4.132
4.113
3.512
3.322
3.297
3.279
3.262
2.837
2.346
2.243
1.617
1.590
1.575
1.554
1.536
1.421
1.403
1.383
1.283
1.256
1.226
1.218
1.203
1.189
1.171
1.076
1.059
0.925
0.909
0.895
0.876
Current Data Parameters
NAME LXY-19
EXPNO 1
PROCNO 1
F2 - Acquisition Parameters
Date_ 20210402
Time 9.21 h
INSTRUM spect
PROBHD Z116098_0436 (
PULPROG zg30
TD 65536
SOLVENT CDCl3
NS 16
DS 2
SWH 8012.820 Hz
FIDRES 0.244532 Hz
AQ 4.0894465 sec
RG 30.51
DW 62.400 usec
DE 6.50 usec
TE 298.0 K
D1 1.00000000 sec
TD0 1
SFO1 400.1524709 MHz
NUC1 1H
P1 9.78 usec
PLW1 15.00300026 W
F2 - Processing parameters
SI 65536
SF 400.1500035 MHz
WDW EM
SSB 0
LB 0.30 Hz
GB 0
PC 1.00
7.5 7.0 6.5 6.0 5.5 5.0 4.5 4.0 3.5 3.0 2.5 2.0 1.5 1.0 ppm
0.99
0.93
0.86
1.89
1.91
1.00
1.80
0.97
0.90
5.13
0.90
1.92
0.91
6.51
3.06
1.11
1.00
1.98
1.03
6.10
3.07
1.93
2.18
9.42
6.21
6.37

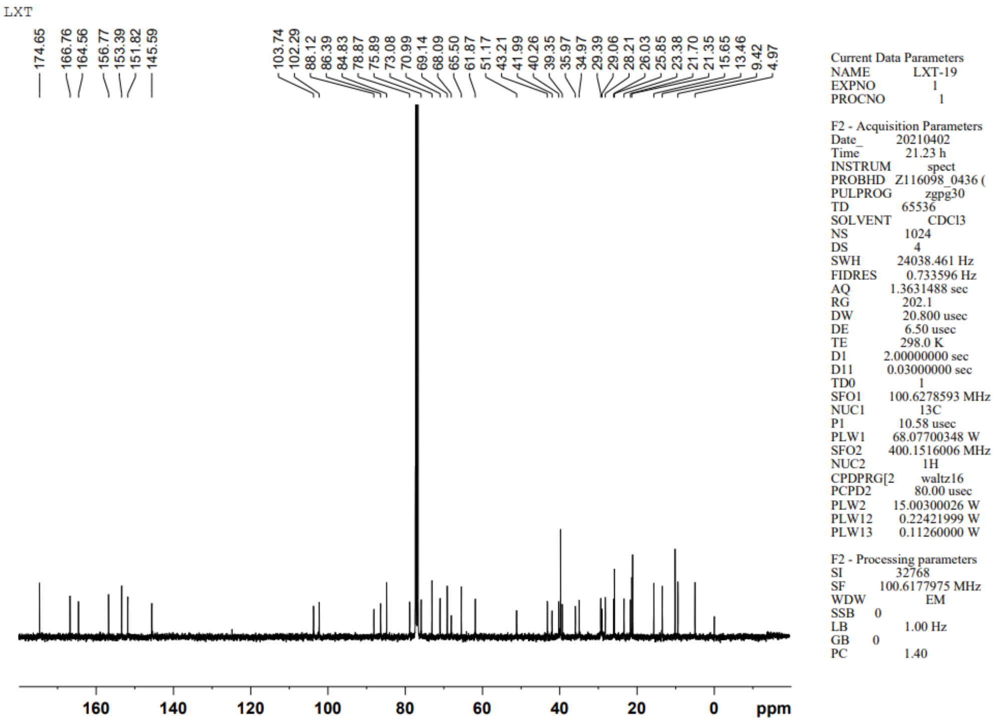
LXT
174.65
166.76
164.56
156.77
153.39
151.82
145.59
103.74
102.29
88.12
86.39
84.83
78.87
75.89
73.08
70.99
69.14
68.09
65.50
61.87
51.17
43.21
41.99
40.26
39.35
35.97
34.97
29.39
29.06
28.21
26.03
25.85
23.38
21.70
21.35
15.65
13.46
9.42
4.97
160
140
120
100
80
60
40
20
0
ppm
Current Data Parameters
NAME LXT-19
EXPNO 1
PROCNO 1
F2 - Acquisition Parameters
Date_ 20210402
Time 21.23 h
INSTRUM spect
PROBHD Z116098_0436 (
PULPROG zgpg30
TD 65536
SOLVENT CDCl3
NS 1024
DS 4
SWH 24038.461 Hz
FIDRES 0.733596 Hz
AQ 1.3631488 sec
RG 202.1
DW 20.800 usec
DE 6.50 usec
TE 298.0 K
D1 2.00000000 sec
D11 0.03000000 sec
TD0 1
SFO1 100.6278593 MHz
NUC1 13C
P1 10.58 usec
PLW1 68.07700348 W
SFO2 400.1516006 MHz
NUC2 1H
CPDPRG[2 waltz16
PCPD2 80.00 usec
PLW2 15.00300026 W
PLW12 0.22421999 W
PLW13 0.11260000 W
F2 - Processing parameters
SI 32768
SF 100.6177975 MHz
WDW EM
SSB 0
LB 1.00 Hz
GB 0
PC 1.40

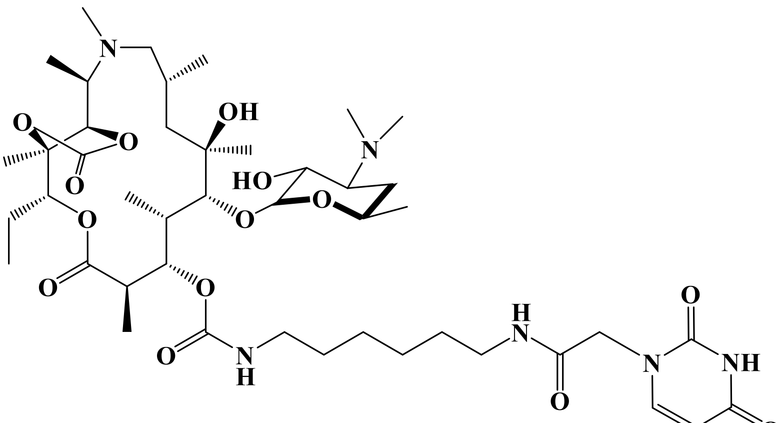

30U

LXT

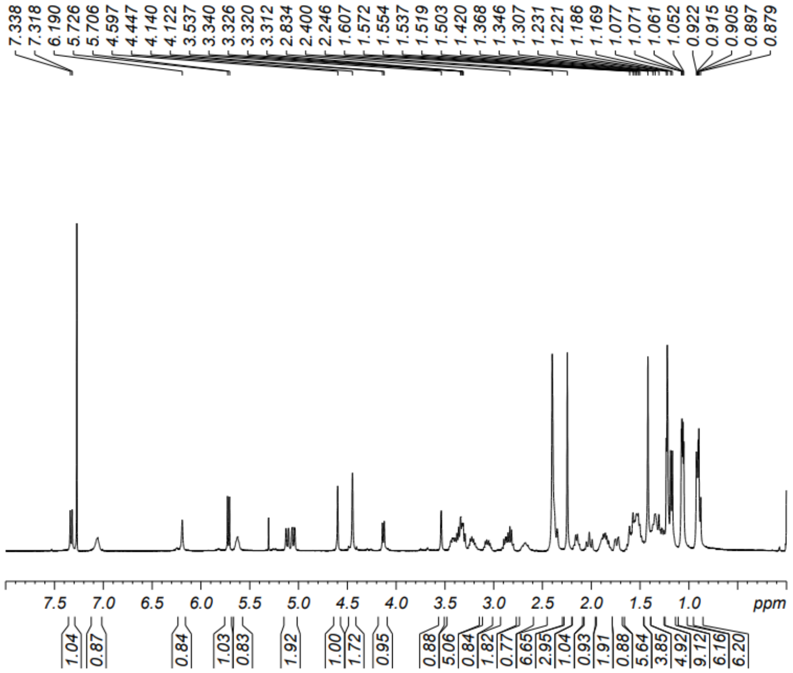

Current Data Parameters
NAME LXT-24
EXPNO 1
PROCNO 1

F2 - Acquisition Parameters
Date_ 20210412
Time 10.11 h
INSTRUM spect
PROBHD Z116098_0436 (
PULPROG zg30
TD 65536
SOLVENT CDCl3
NS 32
DS 2
SWH 8012.820 Hz
FIDRES 0.244532 Hz
AQ 4.0894465 sec
RG 79.39
DW 62.400 usec
DE 6.50 usec
TE 298.0 K
D1 1.00000000 sec
TD0 1
SFO1 400.1524709 MHz
NUC1 1H
P1 9.78 usec
PLW1 15.00300026 W

F2 - Processing parameters
SI 65536
SF 400.1500062 MHz
WDW EM
SSB 0
LB 0.30 Hz
GB 0
PC 1.00

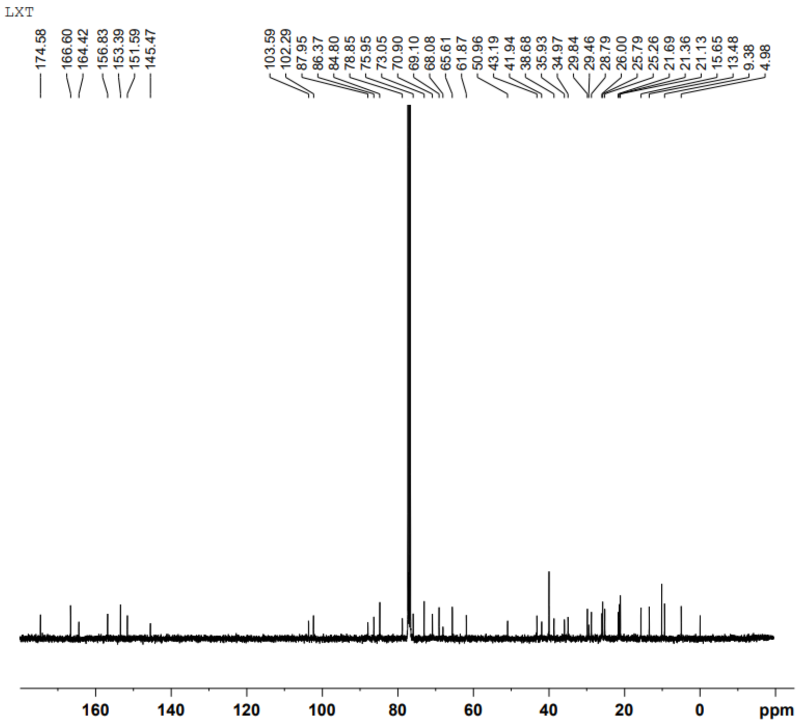

Current Data Parameters
NAME LXT-24
EXPNO 2
PROCNO 1

F2 - Acquisition Parameters
Date_ 20210413
Time 5.11 h
INSTRUM spect
PROBHD Z116098_0436 (
PULPROG zgpg30
TD 65536
SOLVENT CDCl3
NS 1024
DS 4
SWH 24038.461 Hz
FIDRES 0.733596 Hz
AQ 1.3631488 sec
RG 202.1
DW 20.800 usec
DE 6.50 usec
TE 298.0 K
D1 2.00000000 sec
D11 0.03000000 sec
TD0 1
SFO1 100.6278593 MHz
NUC1 13C
P1 10.58 usec
PLW1 68.07700348 W
SFO2 400.1516006 MHz
NUC2 1H
CPDPRG[2 waltz16
PCPD2 80.00 usec
PLW2 15.00300026 W
PLW12 0.22421999 W
PLW13 0.11260000 W

F2 - Processing parameters
SI 32768
SF 100.6177988 MHz
WDW EM
SSB 0
LB 1.00 Hz
GB 0
PC 1.40

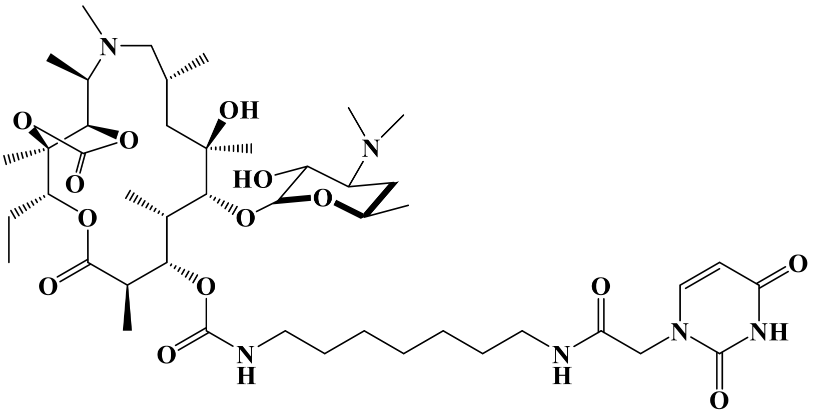

31U

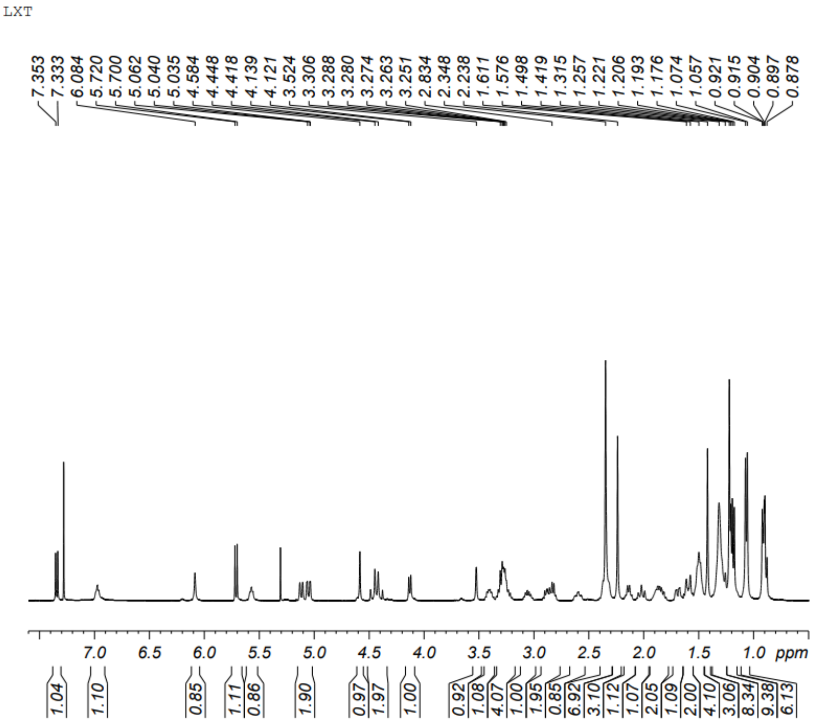

Current Data Parameters
NAME LXT-25
EXPNO 2
PROCNO 1

F2 - Acquisition Parameters
Date_ 20210427
Time 9.10 h
INSTRUM spect
PROBHD Z116098_0436 (
PULPROG zg30
TD 65536
SOLVENT CDCl3
NS 32
DS 2
SWH 8012.820 Hz
FIDRES 0.244532 Hz
AQ 4.0894465 sec
RG 30.51
DW 62.400 usec
DE 6.50 usec
TE 298.0 K
D1 1.00000000 sec
TD0 1
SFO1 400.1524709 MHz
NUC1 1H
P1 9.78 usec
PLW1 15.00300026 W

F2 - Processing parameters
SI 65536
SF 400.1500025 MHz
WDW EM
SSB 0
LB 0.30 Hz
GB 0
PC 1.00

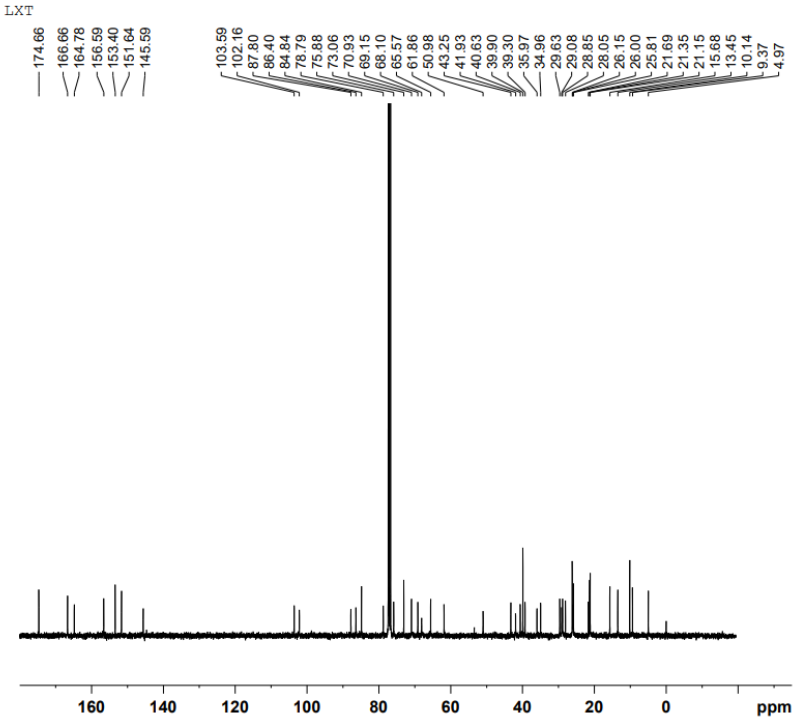

Current Data Parameters
NAME LXT-25
EXPNO 3
PROCNO 1

F2 - Acquisition Parameters
Date_ 20210429
Time 4.44 h
INSTRUM spect
PROBHD Z116098_0436 (
PULPROG zgpg30
TD 65536
SOLVENT CDCl3
NS 750
DS 4
SWH 24038.461 Hz
FIDRES 0.733596 Hz
AQ 1.3631488 sec
RG 202.1
DW 20.800 usec
DE 6.50 usec
TE 298.0 K
D1 2.00000000 sec
D11 0.03000000 sec
TD0 1
SFO1 100.6278593 MHz
NUC1 13C
P1 10.58 usec
PLW1 68.07700348 W
SFO2 400.1516006 MHz
NUC2 1H
CPDPRG[2 waltz16
PCPD2 80.00 usec
PLW2 15.00300026 W
PLW12 0.22421999 W
PLW13 0.11260000 W

F2 - Processing parameters
SI 32768
SF 100.6177975 MHz
WDW EM
SSB 0
LB 1.00 Hz
GB 0
PC 1.40

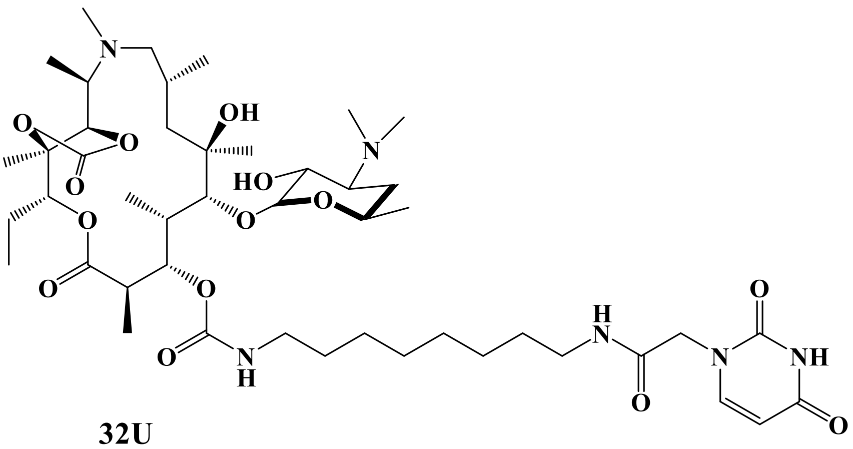

32U

LXT NS=32

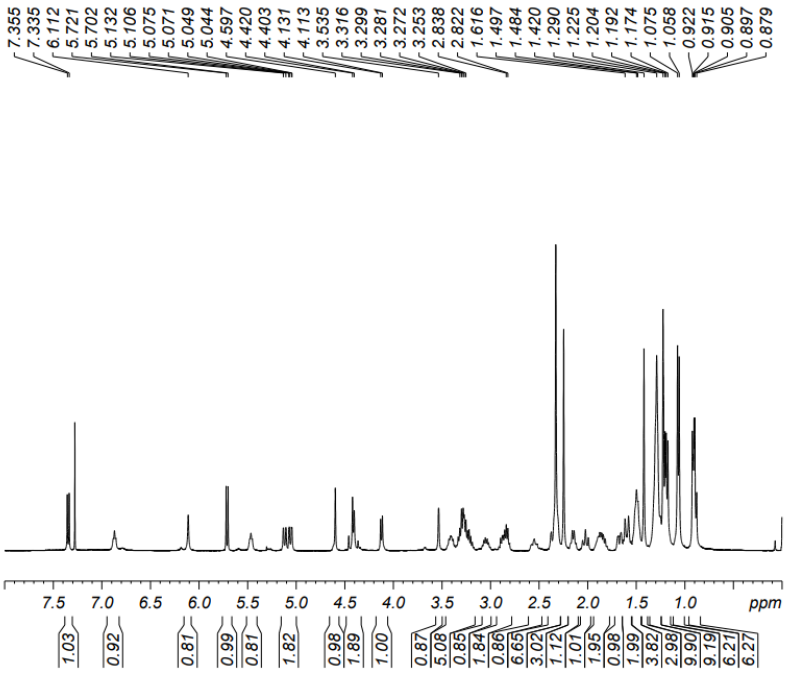

Current Data Parameters
NAME LXT-27
EXPNO 1
PROCNO 1

F2 - Acquisition Parameters
Date_ 20210506
Time 9.31 h
INSTRUM spect
PROBHD Z116098_0436 (
PULPROG zg30
TD 65536
SOLVENT CDCl3
NS 32
DS 2
SWH 8012.820 Hz
FIDRES 0.244532 Hz
AQ 4.0894465 sec
RG 47.35
DW 62.400 usec
DE 6.50 usec
TE 298.0 K
D1 1.00000000 sec
TD0 1
SFO1 400.1524709 MHz
NUC1 1H
P1 9.78 usec
PLW1 15.00300026 W

F2 - Processing parameters
SI 65536
SF 400.1500034 MHz
WDW EM
SSB 0
LB 0.30 Hz
GB 0
PC 1.00

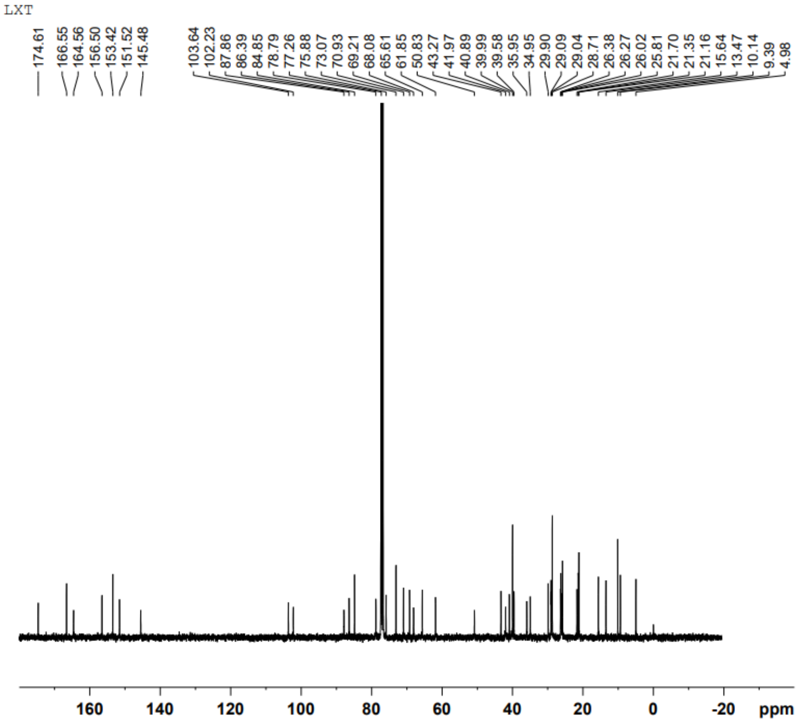

Current Data Parameters
NAME LXT-27
EXPNO 2
PROCNO 1

F2 - Acquisition Parameters
Date_ 20210511
Time 4.25 h
INSTRUM spect
PROBHD Z116098_0436 (
PULPROG zgpg30
TD 65536
SOLVENT CDCl3
NS 1024
DS 4
SWH 24038.461 Hz
FIDRES 0.733596 Hz
AQ 1.3631488 sec
RG 202.1
DW 20.800 usec
DE 6.50 usec
TE 298.0 K
D1 2.00000000 sec
D11 0.03000000 sec
TD0 1
SFO1 100.6278593 MHz
NUC1 13C
P1 10.58 usec
PLW1 68.07700348 W
SFO2 400.1516006 MHz
NUC2 1H
CPDPRG[2 waltz16
PCPD2 80.00 usec
PLW2 15.00300026 W
PLW12 0.22421999 W
PLW13 0.11260000 W

F2 - Processing parameters
SI 32768
SF 100.6177975 MHz
WDW EM
SSB 0
LB 1.00 Hz
GB 0
PC 1.40

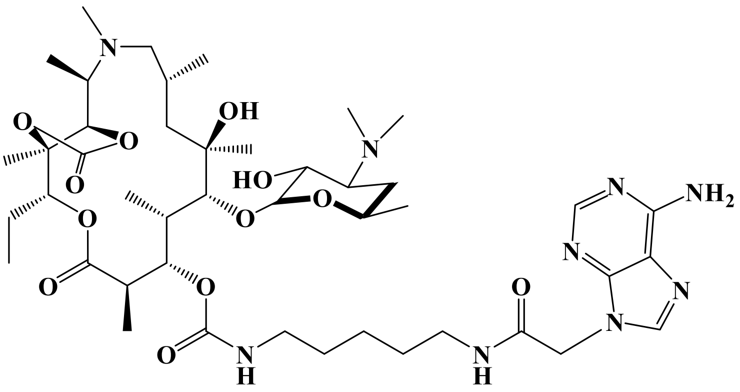

29A

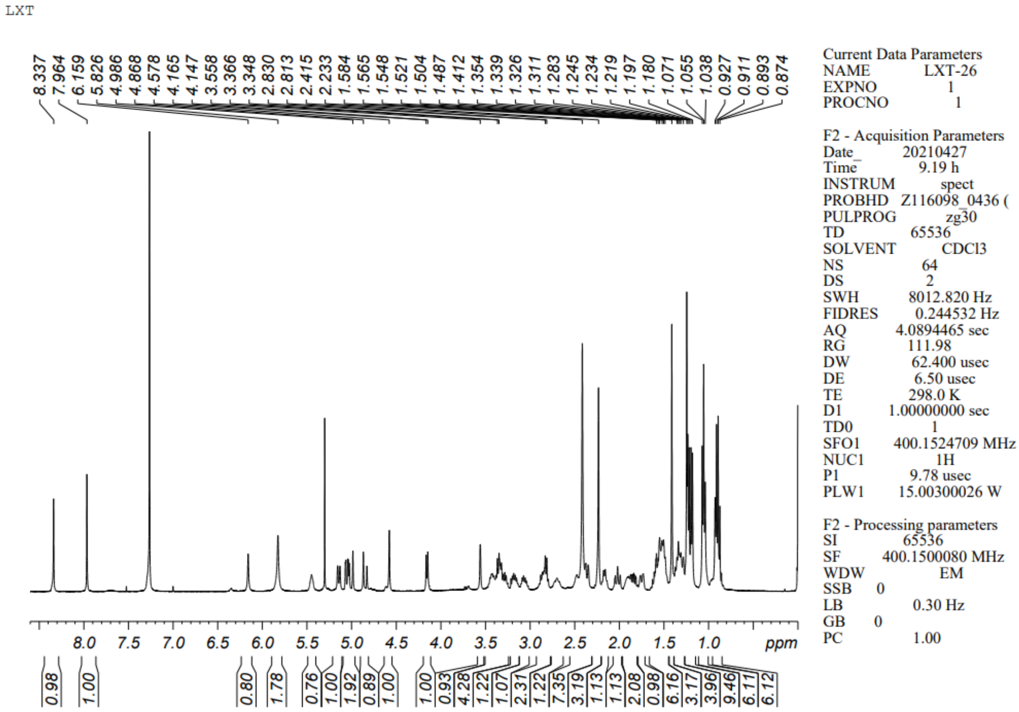
LXT
8.337 7.964 6.159 5.826 4.986 4.868 4.578 4.165 4.147 3.558 3.366 3.348 2.830 2.813 2.415 2.233 1.584 1.565 1.548 1.521 1.504 1.487 1.412 1.354 1.339 1.326 1.311 1.283 1.245 1.234 1.219 1.197 1.180 1.071 1.055 1.038 0.927 0.911 0.893 0.874
8.0 7.5 7.0 6.5 6.0 5.5 5.0 4.5 4.0 3.5 3.0 2.5 2.0 1.5 1.0 ppm
0.98 1.00 0.80 1.78 0.76 1.00 1.92 0.89 1.00 1.00 0.93 4.28 1.22 1.07 2.31 1.22 7.35 3.19 1.13 1.13 2.08 0.98 6.16 3.17 3.96 9.46 6.11 6.12
Current Data Parameters
NAME LXT-26
EXPNO 1
PROCNO 1
F2 - Acquisition Parameters
Date_ 20210427
Time 9.19 h
INSTRUM spect
PROBHD Z116098_0436 (
PULPROG zg30
TD 65536
SOLVENT CDCl3
NS 64
DS 2
SWH 8012.820 Hz
FIDRES 0.244532 Hz
AQ 4.0894465 sec
RG 111.98
DW 62.400 usec
DE 6.50 usec
TE 298.0 K
D1 1.00000000 sec
TD0 1
SFO1 400.1524709 MHz
NUC1 1H
P1 9.78 usec
PLW1 15.00300026 W
F2 - Processing parameters
SI 65536
SF 400.1500080 MHz
WDW EM
SSB 0
LB 0.30 Hz
GB 0
PC 1.00

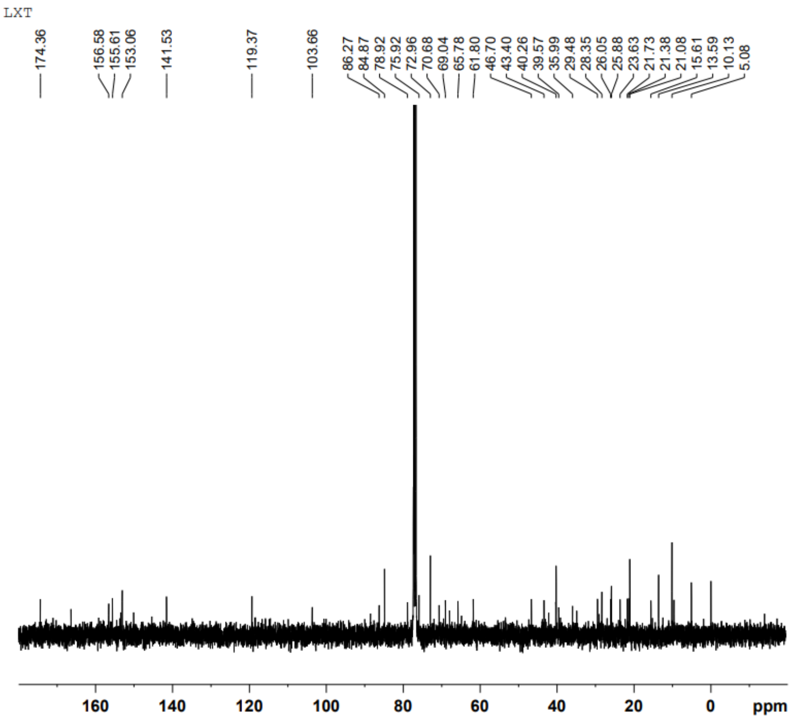

Current Data Parameters
NAME LXT-26
EXPNO 2
PROCNO 1

F2 - Acquisition Parameters
Date_ 20210429
Time 5.31 h
INSTRUM spect
PROBHD Z116098_0436 (
PULPROG zgpg30
TD 65536
SOLVENT CDCl3
NS 750
DS 4
SWH 24038.461 Hz
FIDRES 0.733596 Hz
AQ 1.3631488 sec
RG 202.1
DW 20.800 usec
DE 6.50 usec
TE 298.0 K
D1 2.00000000 sec
D11 0.03000000 sec
TD0 1
SFO1 100.6278593 MHz
NUC1 13C
P1 10.58 usec
PLW1 68.07700348 W
SFO2 400.1516006 MHz
NUC2 1H
CPDPRG[2 waltz16
PCPD2 80.00 usec
PLW2 15.00300026 W
PLW12 0.22421999 W
PLW13 0.11260000 W

F2 - Processing parameters
SI 32768
SF 100.6177975 MHz
WDW EM
SSB 0
LB 1.00 Hz
GB 0
PC 1.40

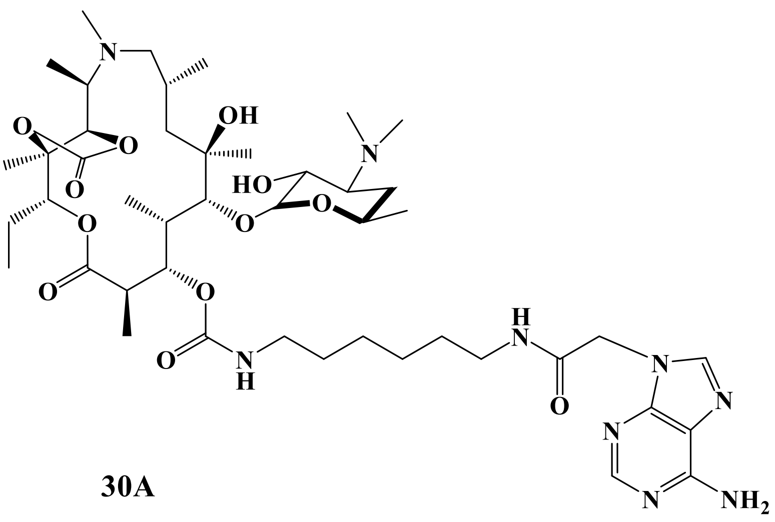

30A

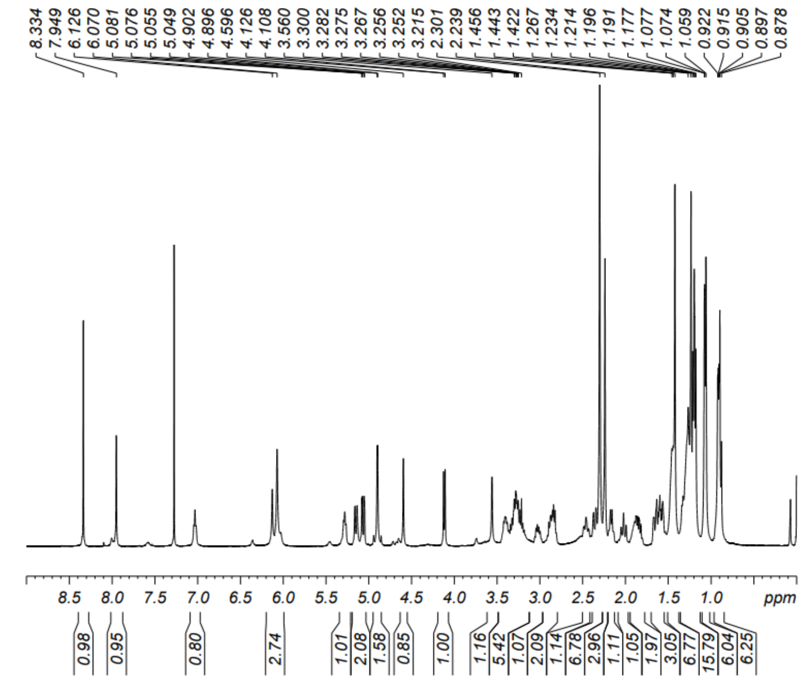

Current Data Parameters
NAME LXT-28-2
EXPNO 1
PROCNO 1

F2 - Acquisition Parameters
Date_ 20210512
Time 9.56 h
INSTRUM spect
PROBHD Z116098_0436 (
PULPROG zg30
TD 65536
SOLVENT CDCl3
NS 64
DS 2
SWH 8012.820 Hz
FIDRES 0.244532 Hz
AQ 4.0894465 sec
RG 64.09
DW 62.400 usec
DE 6.50 usec
TE 298.0 K
D1 1.00000000 sec
TD0 1
SFO1 400.1524709 MHz
NUC1 1H
P1 9.78 usec
PLW1 15.00300026 W

F2 - Processing parameters
SI 65536
SF 400.1500050 MHz
WDW EM
SSB 0
LB 0.30 Hz
GB 0
PC 1.00

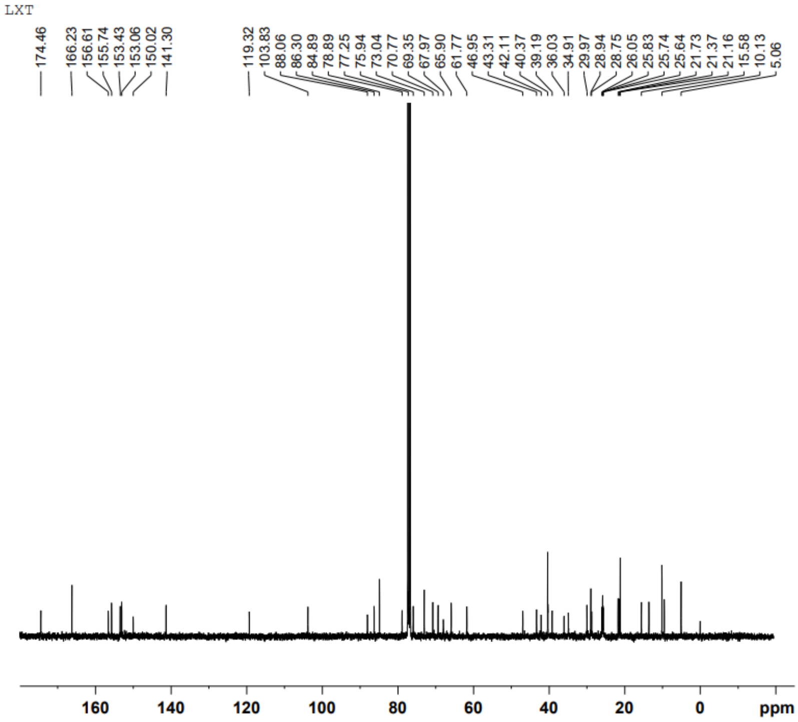

Current Data Parameters
NAME LXT-28
EXPNO 3
PROCNO 1

F2 - Acquisition Parameters
Date_ 20210514
Time 4.08 h
INSTRUM spect
PROBHD Z116098_0436 (
PULPROG zgpg30
TD 65536
SOLVENT CDCl3
NS 1024
DS 4
SWH 24038.461 Hz
FIDRES 0.733596 Hz
AQ 1.3631488 sec
RG 202.1
DW 20.800 usec
DE 6.50 usec
TE 298.0 K
D1 2.00000000 sec
D11 0.03000000 sec
TD0 1
SFO1 100.6278593 MHz
NUC1 13C
P1 10.58 usec
PLW1 68.07700348 W
SFO2 400.1516006 MHz
NUC2 1H
CPDPRG[2 waltz16
PCPD2 80.00 usec
PLW2 15.00300026 W
PLW12 0.22421999 W
PLW13 0.11260000 W

F2 - Processing parameters
SI 32768
SF 100.6177975 MHz
WDW EM
SSB 0
LB 1.00 Hz
GB 0
PC 1.40

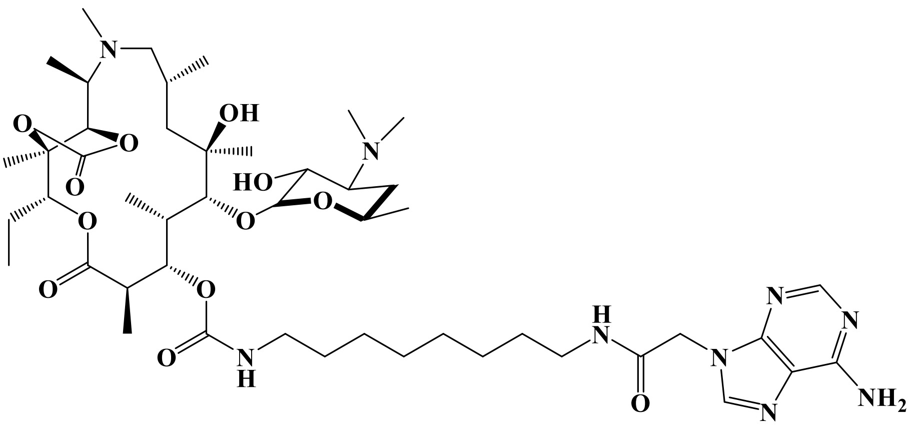

32A

LXT NS=64

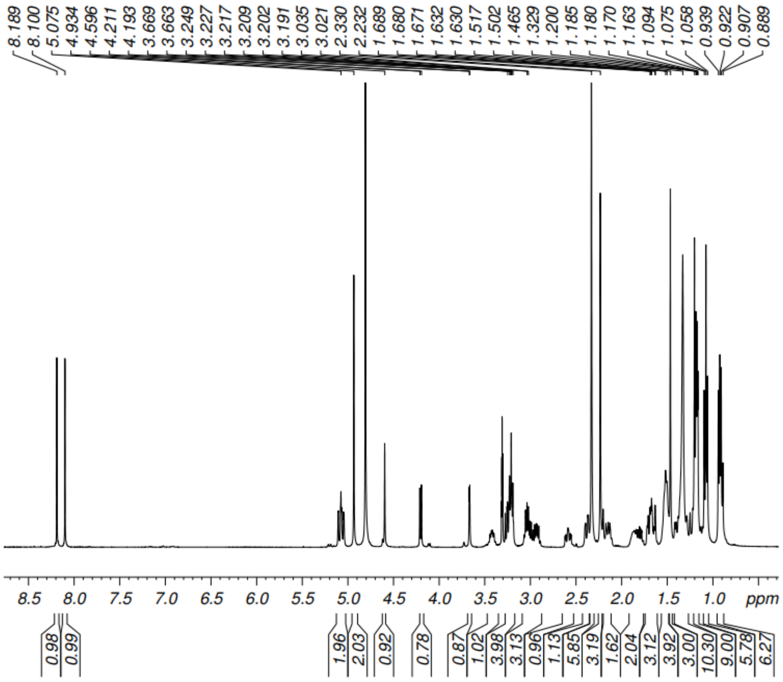

Current Data Parameters
NAME LXT-74
EXPNO 1
PROCNO 1

F2 - Acquisition Parameters
Date_ 20220114
Time 14.27 h
INSTRUM spect
PROBHD Z116098_0436 (
PULPROG zg30
TD 65536
SOLVENT MeOD
NS 64
DS 2
SWH 8012.820 Hz
FIDRES 0.244532 Hz
AQ 4.0894465 sec
RG 30.51
DW 62.400 usec
DE 6.50 usec
TE 298.0 K
D1 1.00000000 sec
TD0 1
SFO1 400.1524709 MHz
NUC1 1H
P1 9.78 usec
PLW1 15.00300026 W

F2 - Processing parameters
SI 65536
SF 400.1500085 MHz
WDW EM
SSB 0
LB 0.30 Hz
GB 0
PC 1.00

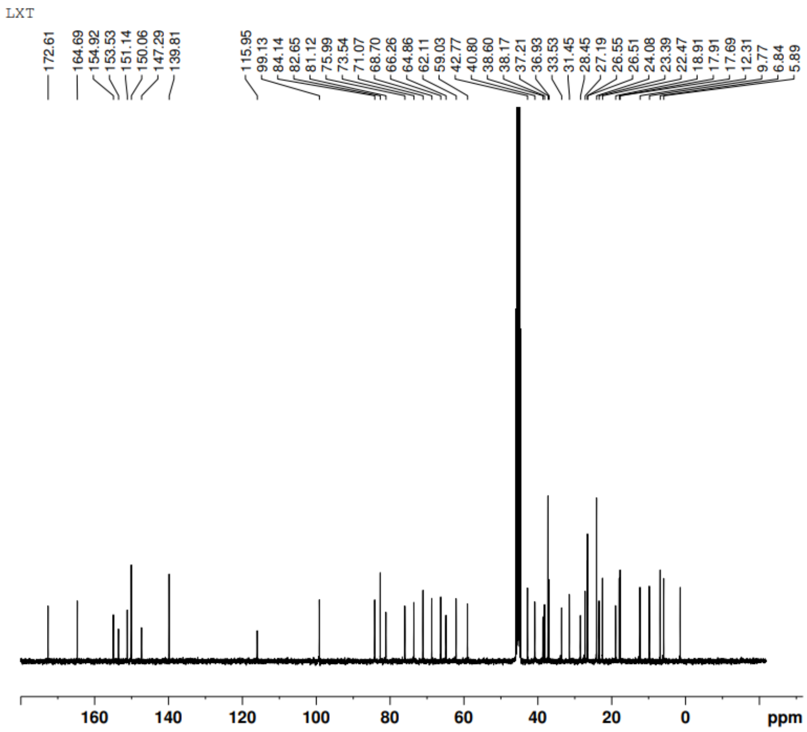

Current Data Parameters
NAME LXT-74
EXPNO 2
PROCNO 1

F2 - Acquisition Parameters
Date_ 20220115
Time 11.17 h
INSTRUM spect
PROBHD Z116098_0436 (
PULPROG zgpg30
TD 65536
SOLVENT MeOD
NS 1024
DS 4
SWH 24038.461 Hz
FIDRES 0.733596 Hz
AQ 1.3631488 sec
RG 202.1
DW 20.800 usec
DE 6.50 usec
TE 298.0 K
D1 2.00000000 sec
D11 0.03000000 sec
TD0 1
SFO1 100.6278593 MHz
NUC1 13C
P1 10.58 usec
PLW1 68.07700348 W
SFO2 400.1516006 MHz
NUC2 1H
CPDPRG[2 waltz16
PCPD2 80.00 usec
PLW2 15.00300026 W
PLW12 0.22421999 W
PLW13 0.11260000 W

F2 - Processing parameters
SI 32768
SF 100.6180361 MHz
WDW EM
SSB 0
LB 1.00 Hz
GB 0
PC 1.40

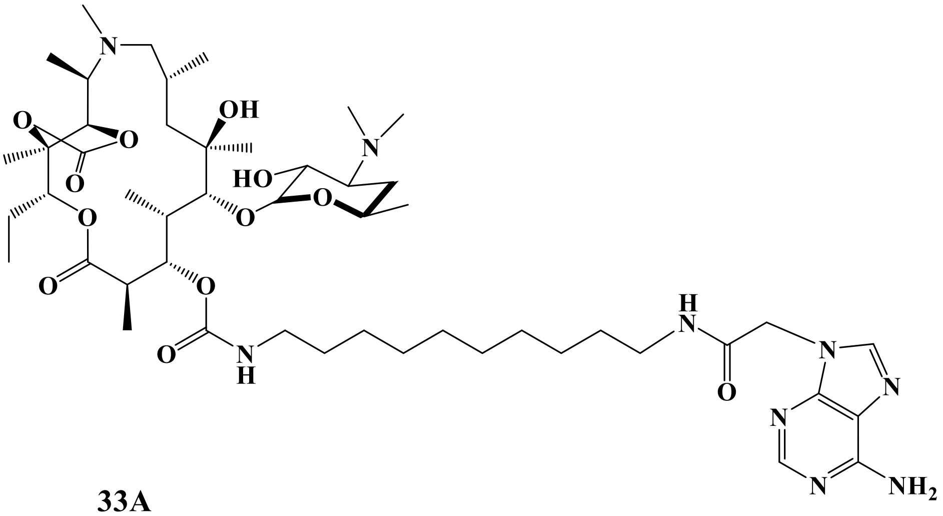

33A

LXT NS=64

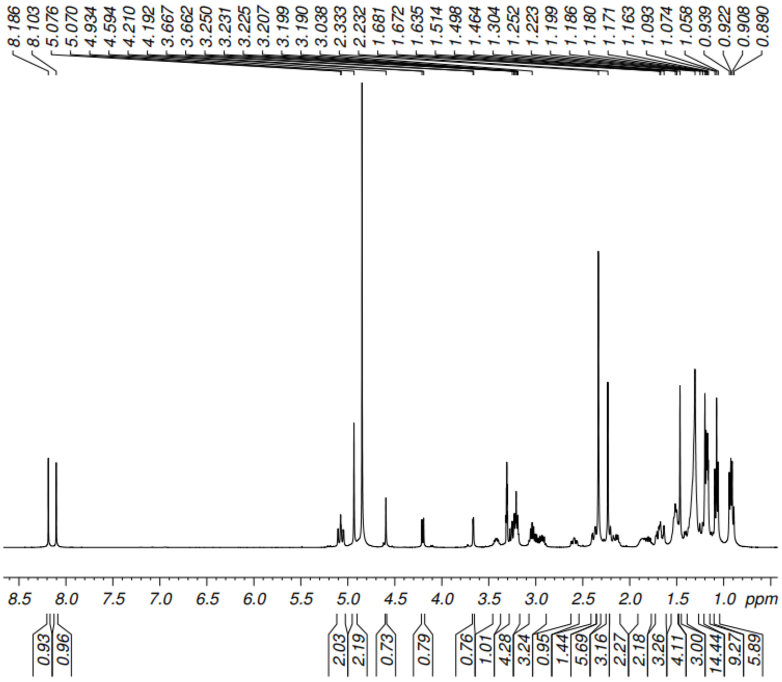

Current Data Parameters
NAME LXT-91
EXPNO 1
PROCNO 1

F2 - Acquisition Parameters
Date_ 20220422
Time 8.50 h
INSTRUM spect
PROBHD Z116098_0436 (
PULPROG zg30
TD 65536
SOLVENT MeOD
NS 64
DS 2
SWH 8012.820 Hz
FIDRES 0.244532 Hz
AQ 4.0894465 sec
RG 30.51
DW 62.400 usec
DE 6.50 usec
TE 298.0 K
D1 1.00000000 sec
TD0 1
SFO1 400.1524709 MHz
NUC1 1H
P1 9.78 usec
PLW1 15.00300026 W

F2 - Processing parameters
SI 65536
SF 400.1500090 MHz
WDW EM
SSB 0
LB 0.30 Hz
GB 0
PC 1.00

13C

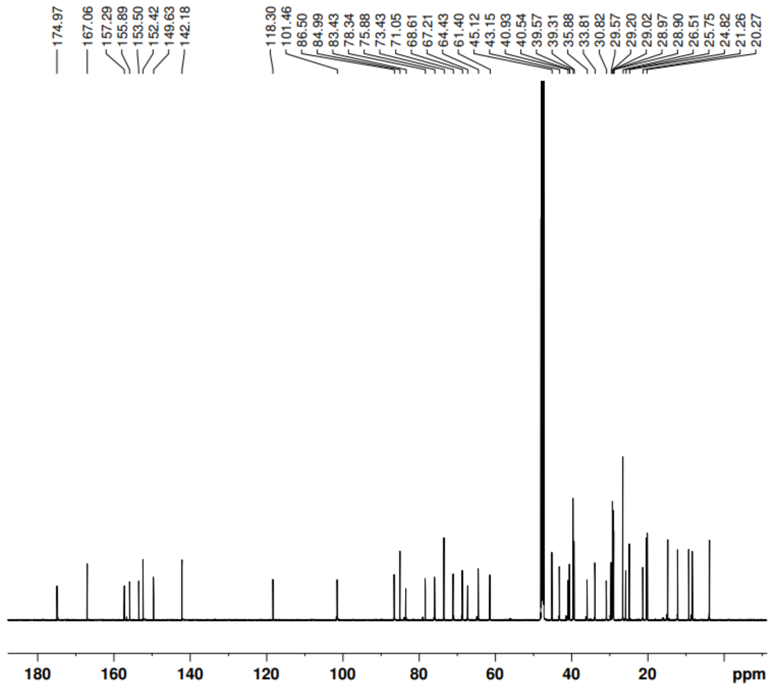

Current Data Parameters
NAME LXT-91
EXPNO 1
PROCNO 1

F2 - Acquisition Parameters
Date_ 20220426
Time 12.55 h
INSTRUM spect
PROBHD Z152837_0001 (
PULPROG zgpg30
TD 65536
SOLVENT MeOD
NS 951
DS 4
SWH 42613.637 Hz
FIDRES 1.300465 Hz
AQ 0.7689557 sec
RG 187.62
DW 11.733 usec
DE 18.00 usec
TE 298.0 K
D1 2.00000000 sec
D11 0.03000000 sec
TD0 1
SFO1 176.0704628 MHz
NUC1 13C
P1 12.87 usec
PLW1 26.46899986 W
SFO2 700.1528006 MHz
NUC2 1H
CPDPRG[2 waltz16
PCPD2 65.00 usec
PLW2 10.69499969 W
PLW12 0.26130000 W
PLW13 0.13158000 W

F2 - Processing parameters
SI 32768
SF 176.0528575 MHz
WDW EM
SSB 0
LB 1.00 Hz
GB 0
PC 1.40

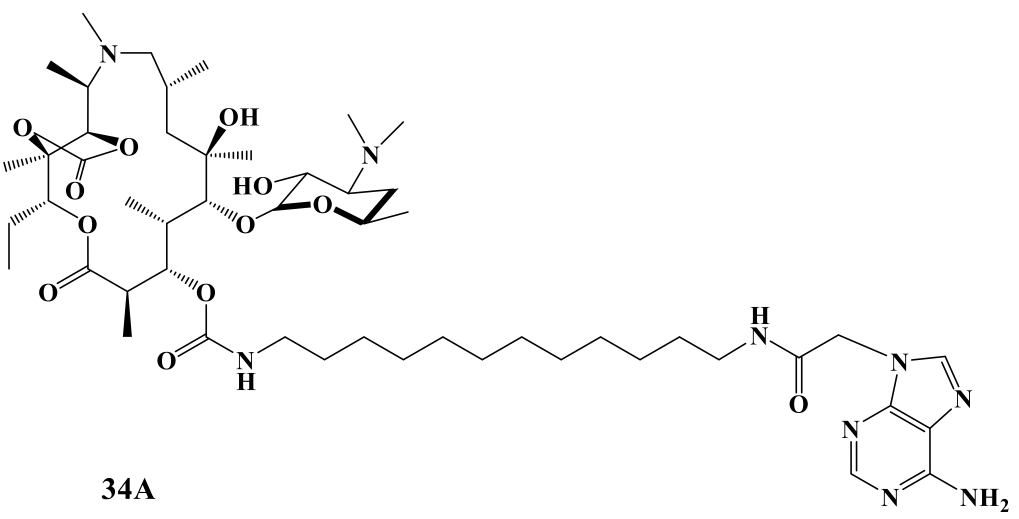

34A

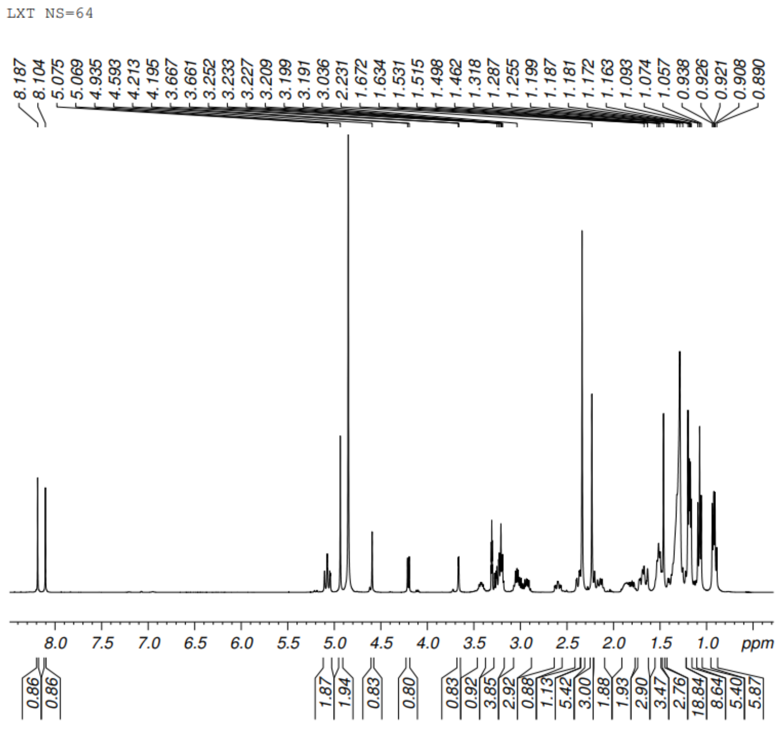

Current Data Parameters
NAME LXT-89
EXPNO 1
PROCNO 1

F2 - Acquisition Parameters
Date_ 20220418
Time 10.20 h
INSTRUM spect
PROBHD Z116098_0436 (
PULPROG zg30
TD 65536
SOLVENT MeOD
NS 64
DS 2
SWH 8012.820 Hz
FIDRES 0.244532 Hz
AQ 4.0894465 sec
RG 30.51
DW 62.400 usec
DE 6.50 usec
TE 298.0 K
D1 1.00000000 sec
TD0 1
SFO1 400.1524709 MHz
NUC1 1H
P1 9.78 usec
PLW1 15.00300026 W

F2 - Processing parameters
SI 65536
SF 400.1500087 MHz
WDW EM
SSB 0
LB 0.30 Hz
GB 0
PC 1.00

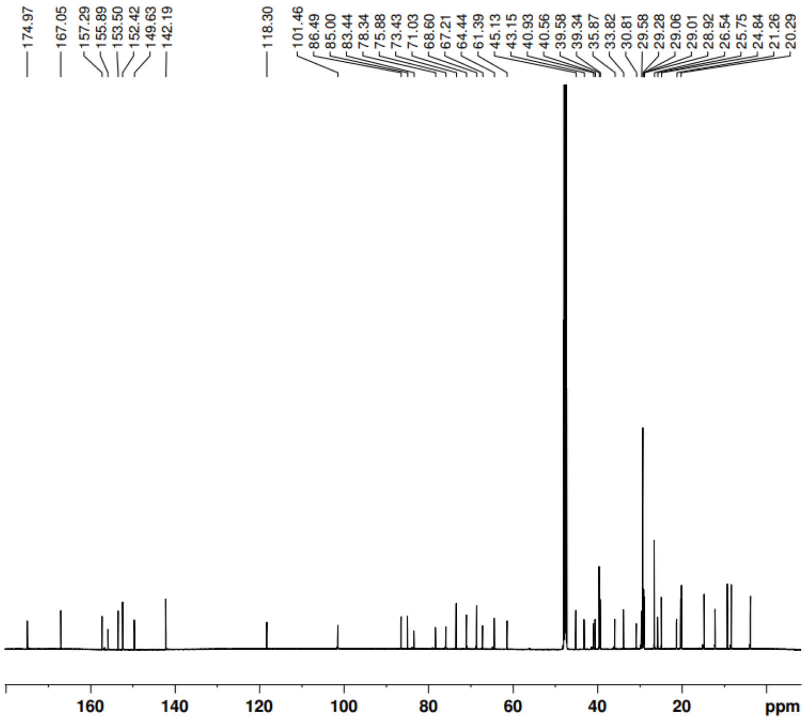

Current Data Parameters
NAME LXT-89
EXPNO 2
PROCNO 1

F2 - Acquisition Parameters
Date_ 20220419
Time 16.52 h
INSTRUM spect
PROBHD Z152837_0001 (
PULPROG zgpg30
TD 65536
SOLVENT MeOD
NS 671
DS 4
SWH 42613.637 Hz
FIDRES 0.650232 Hz
AQ 0.7689557 sec
RG 187.62
DW 11.733 usec
DE 18.00 usec
TE 298.0 K
D1 2.00000000 sec
D11 0.03000000 sec
TD0 1
SFO1 176.0704628 MHz
NUC1 13C
P1 12.00 usec
PLW1 26.46899986 W
SFO2 700.1528006 MHz
NUC2 1H
CPDPRG[2 waltz16
PCPD2 65.00 usec
PLW2 10.69499969 W
PLW12 0.36452001 W
PLW13 0.18355000 W

F2 - Processing parameters
SI 32768
SF 176.0528575 MHz
WDW EM
SSB 0
LB 1.00 Hz
GB 0
PC 1.40

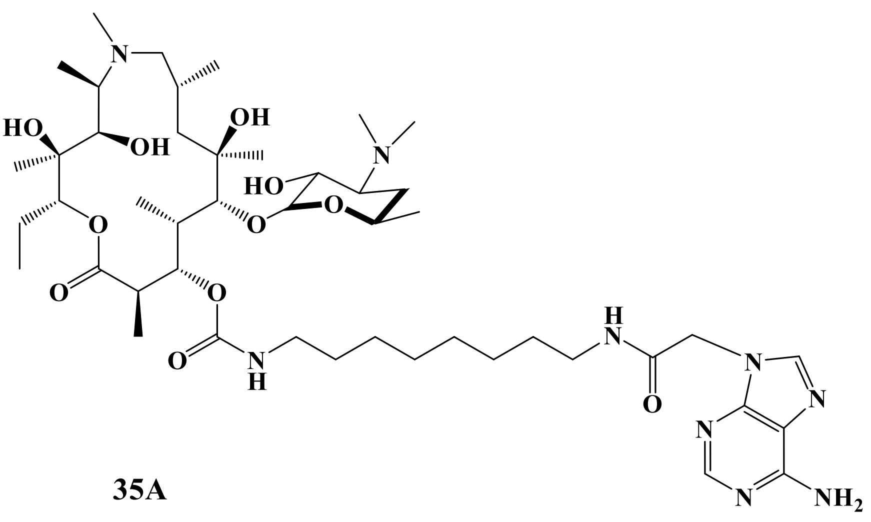

35A

LXT NS=64

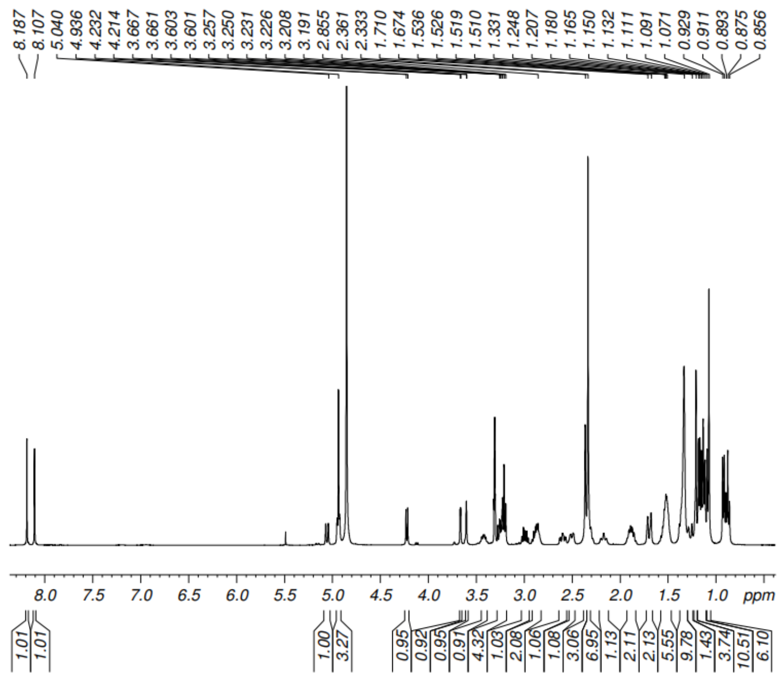

Current Data Parameters
NAME LXT-88
EXPNO 1
PROCNO 1

F2 - Acquisition Parameters
Date_ 20220419
Time 16.17 h
INSTRUM spect
PROBHD Z116098_0436 (
PULPROG zg30
TD 65536
SOLVENT MeOD
NS 64
DS 2
SWH 8012.820 Hz
FIDRES 0.244532 Hz
AQ 4.0894465 sec
RG 64.09
DW 62.400 usec
DE 6.50 usec
TE 298.0 K
D1 1.00000000 sec
TD0 1
SFO1 400.1524709 MHz
NUC1 1H
P1 9.78 usec
PLW1 15.00300026 W

F2 - Processing parameters
SI 65536
SF 400.1500091 MHz
WDW EM
SSB 0
LB 0.30 Hz
GB 0
PC 1.00

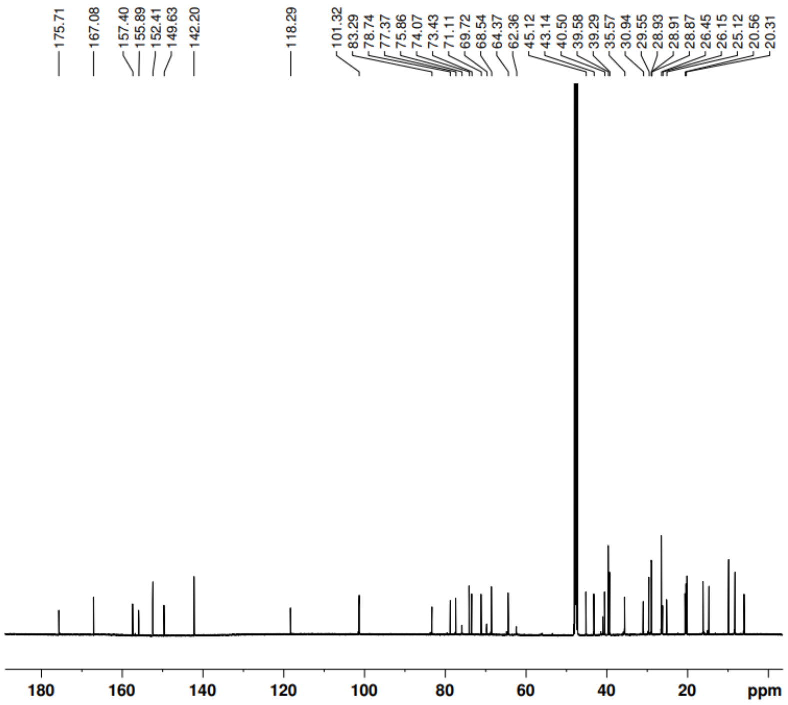

Current Data Parameters
NAME LXT-88
EXPNO 2
PROCNO 1

F2 - Acquisition Parameters
Date_ 20220421
Time 15.19 h
INSTRUM spect
PROBHD Z152837_0001 (
PULPROG zgpg30
TD 65536
SOLVENT MeOD
NS 640
DS 4
SWH 42613.637 Hz
FIDRES 0.650232 Hz
AQ 0.7689557 sec
RG 187.62
DW 11.733 usec
DE 18.00 usec
TE 298.0 K
D1 2.00000000 sec
D11 0.03000000 sec
TD0 1
SFO1 176.0704628 MHz
NUC1 13C
P1 12.00 usec
PLW1 26.46899986 W
SFO2 700.1528006 MHz
NUC2 1H
CPDPRG[2 waltz16
PCPD2 65.00 usec
PLW2 10.69499969 W
PLW12 0.36452001 W
PLW13 0.18355000 W

F2 - Processing parameters
SI 32768
SF 176.0528575 MHz
WDW EM
SSB 0
LB 1.00 Hz
GB 0
PC 1.40

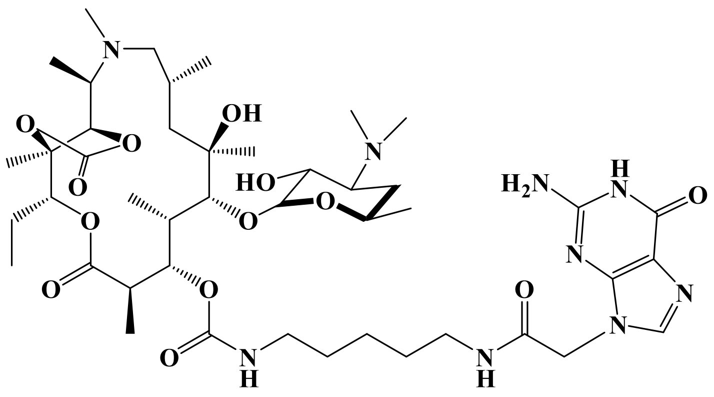

29G

LXT,NS=64

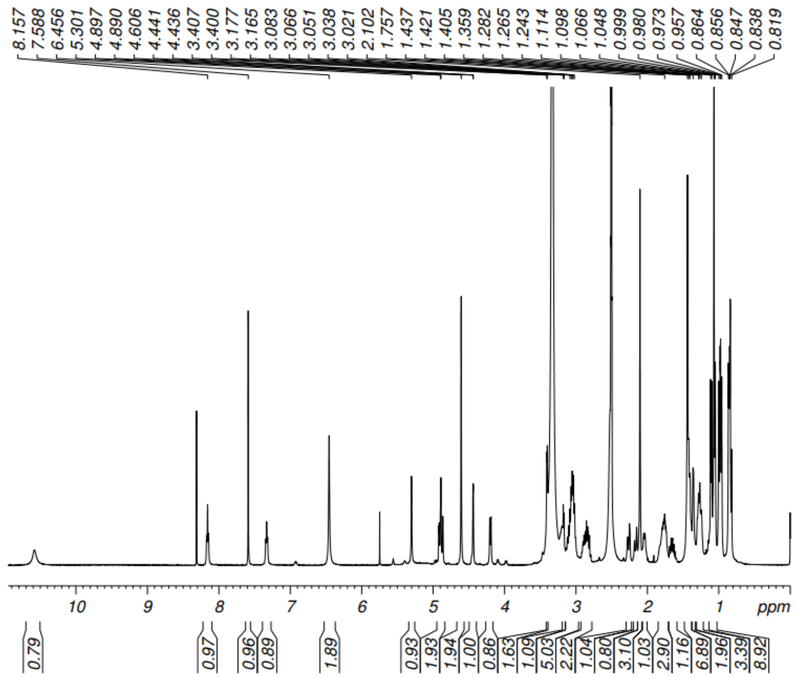

Current Data Parameters
NAME LXT-32-1
EXPNO 1
PROCNO 1

F2 - Acquisition Parameters
Date_ 20210617
Time 9.17 h
INSTRUM spect
PROBHD Z116098_0436 (
PULPROG zg30
TD 65536
SOLVENT DMSO
NS 64
DS 2
SWH 8012.820 Hz
FIDRES 0.244532 Hz
AQ 4.0894465 sec
RG 79.39
DW 62.400 usec
DE 6.50 usec
TE 303.0 K
D1 1.00000000 sec
TD0 1
SFO1 400.1524709 MHz
NUC1 1H
P1 9.78 usec
PLW1 15.00300026 W

F2 - Processing parameters
SI 65536
SF 400.1500027 MHz
WDW EM
SSB 0
LB 0.30 Hz
GB 0
PC 1.00

13C

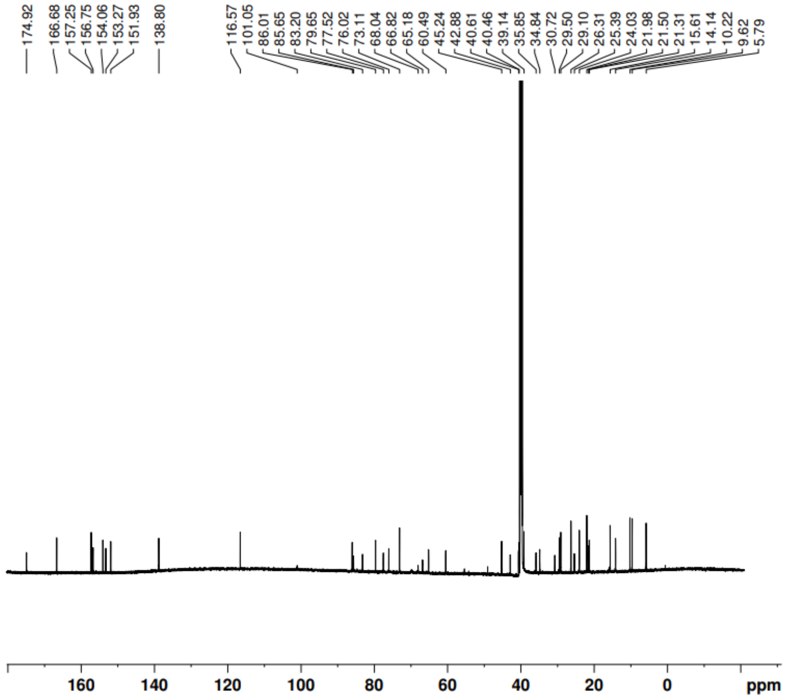

Current Data Parameters
NAME LXT-32-1
EXPNO 1
PROCNO 1

F2 - Acquisition Parameters
Date_ 20210618
Time 11.44 h
INSTRUM spect
PROBHD Z152837_0001 (
PULPROG zgpg30
TD 65536
SOLVENT DMSO
NS 600
DS 4
SWH 42613.637 Hz
FIDRES 0.650232 Hz
AQ 0.7689557 sec
RG 187.62
DW 11.733 usec
DE 18.00 usec
TE 298.0 K
D1 2.00000000 sec
D11 0.03000000 sec
TD0 1
SFO1 176.0704628 MHz
NUC1 13C
P1 12.00 usec
PLW1 26.46899986 W
SFO2 700.1528006 MHz
NUC2 1H
CPDPRG[2 waltz16
PCPD2 65.00 usec
PLW2 10.69499969 W
PLW12 0.36452001 W
PLW13 0.18355000 W

F2 - Processing parameters
SI 32768
SF 176.0528575 MHz
WDW EM
SSB 0
LB 1.00 Hz
GB 0
PC 1.40

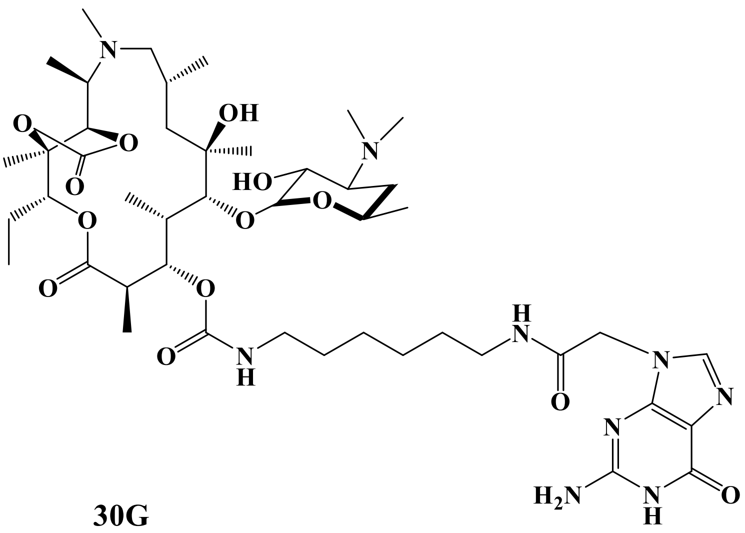

30G

LXT,NS=64

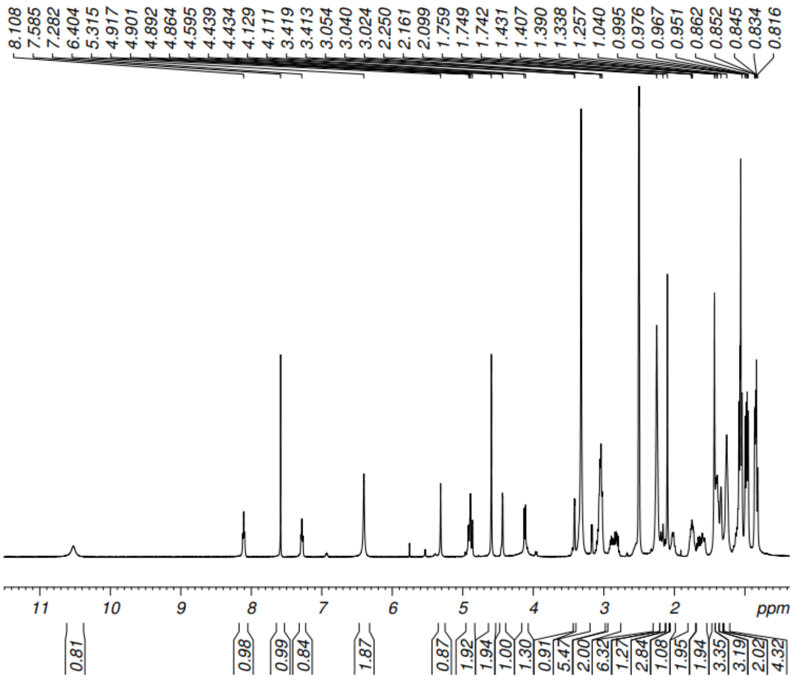

Current Data Parameters
NAME LXT-30-2
EXPNO 1
PROCNO 1

F2 - Acquisition Parameters
Date_ 20210528
Time 8.45 h
INSTRUM spect
PROBHD Z116098_0436 (
PULPROG zg30
TD 65536
SOLVENT DMSO
NS 64
DS 2
SWH 8012.820 Hz
FIDRES 0.244532 Hz
AQ 4.0894465 sec
RG 98.25
DW 62.400 usec
DE 6.50 usec
TE 296.9 K
D1 1.00000000 sec
TD0 1
SFO1 400.1524709 MHz
NUC1 1H
P1 9.78 usec
PLW1 15.00300026 W

F2 - Processing parameters
SI 65536
SF 400.1500031 MHz
WDW EM
SSB 0
LB 0.30 Hz
GB 0
PC 1.00

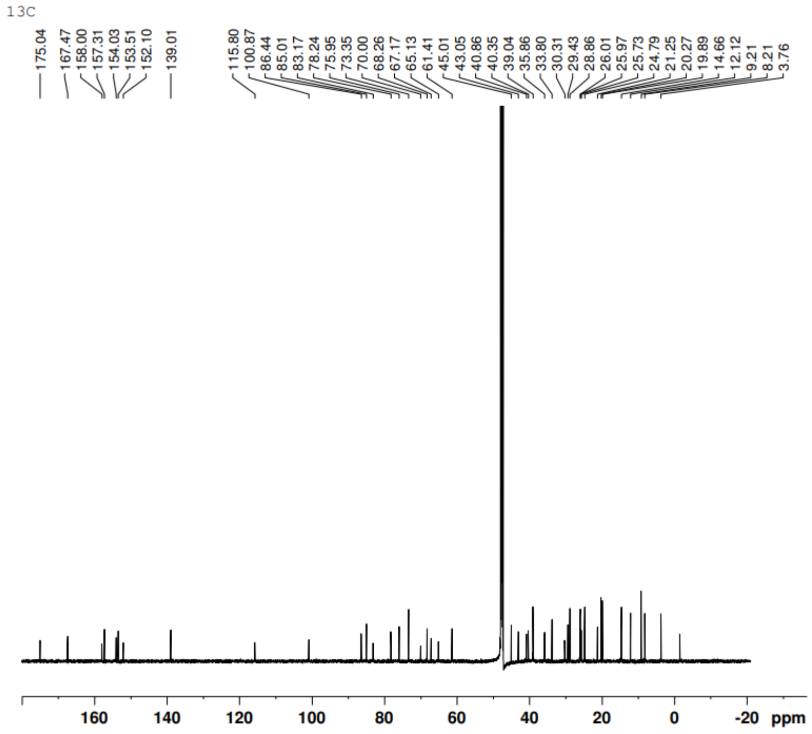

Current Data Parameters
NAME LXT-30-C
EXPNO 1
PROCNO 1

F2 - Acquisition Parameters
Date_ 20210728
Time 15.29 h
INSTRUM spect
PROBHD Z152837_0001 (
PULPROG zgpg30
TD 65536
SOLVENT MeOD
NS 407
DS 4
SWH 42613.637 Hz
FIDRES 0.650232 Hz
AQ 0.7689557 sec
RG 187.62
DW 11.733 usec
DE 18.00 usec
TE 298.0 K
D1 2.00000000 sec
D11 0.03000000 sec
TD0 1
SFO1 176.0704628 MHz
NUC1 13C
P1 12.00 usec
PLW1 26.46899986 W
SFO2 700.1528006 MHz
NUC2 1H
CPDPRG[2 waltz16
PCPD2 65.00 usec
PLW2 10.69499969 W
PLW12 0.36452001 W
PLW13 0.18355000 W

F2 - Processing parameters
SI 32768
SF 176.0528575 MHz
WDW EM
SSB 0
LB 1.00 Hz
GB 0
PC 1.40

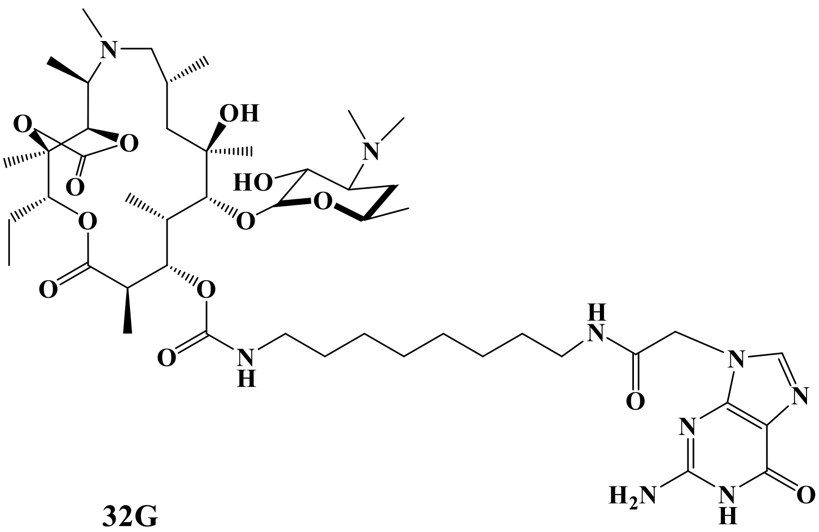

32G

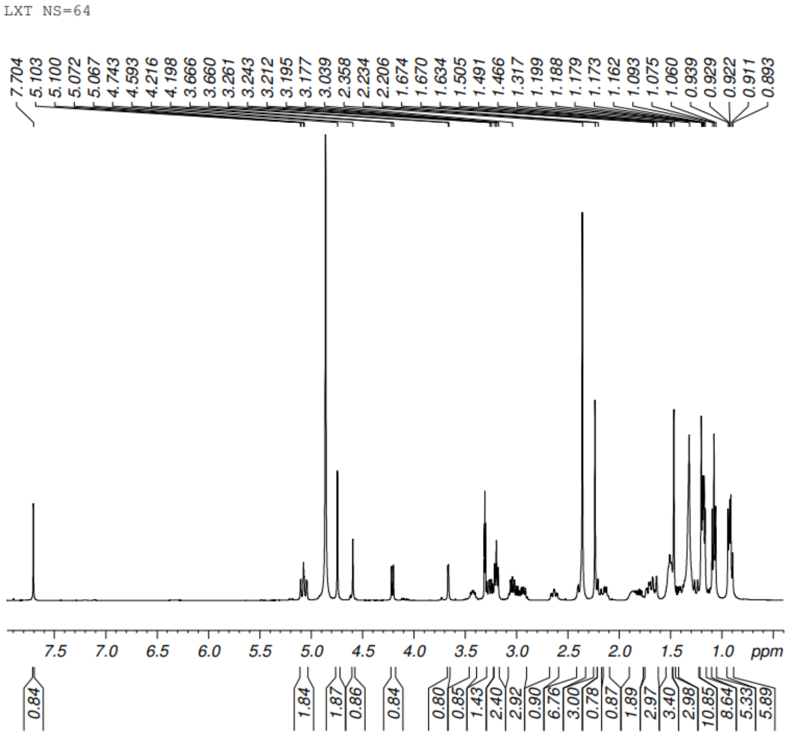

Current Data Parameters
NAME LXT-87
EXPNO 1
PROCNO 1

F2 - Acquisition Parameters
Date_ 20220418
Time 10.07 h
INSTRUM spect
PROBHD Z116098_0436 (
PULPROG zg30
TD 65536
SOLVENT MeOD
NS 64
DS 2
SWH 8012.820 Hz
FIDRES 0.244532 Hz
AQ 4.0894465 sec
RG 47.35
DW 62.400 usec
DE 6.50 usec
TE 298.0 K
D1 1.00000000 sec
TD0 1
SFO1 400.1524709 MHz
NUC1 1H
P1 9.78 usec
PLW1 15.00300026 W

F2 - Processing parameters
SI 65536
SF 400.1500090 MHz
WDW EM
SSB 0
LB 0.30 Hz
GB 0
PC 1.00

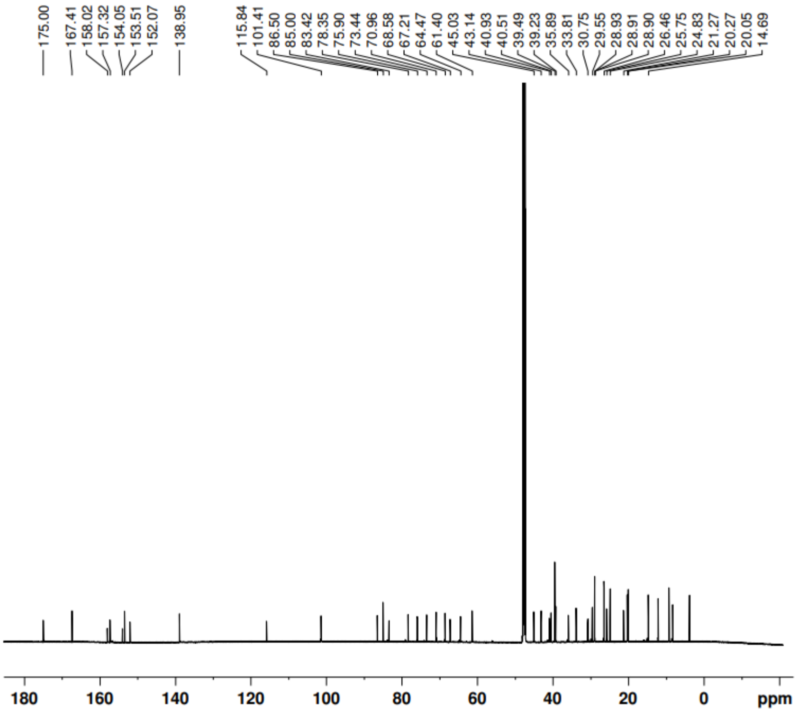

Current Data Parameters
NAME LXT-87
EXPNO 2
PROCNO 1

F2 - Acquisition Parameters
Date_ 20220419
Time 17.19 h
INSTRUM spect
PROBHD Z152837_0001 (
PULPROG zgpg30
TD 65536
SOLVENT MeOD
NS 474
DS 4
SWH 42613.637 Hz
FIDRES 0.650232 Hz
AQ 0.7689557 sec
RG 187.62
DW 11.733 usec
DE 18.00 usec
TE 298.0 K
D1 2.00000000 sec
D11 0.03000000 sec
TD0 1
SFO1 176.0704628 MHz
NUC1 13C
P1 12.00 usec
PLW1 26.46899986 W
SFO2 700.1528006 MHz
NUC2 1H
CPDPRG[2 waltz16
PCPD2 65.00 usec
PLW2 10.69499969 W
PLW12 0.36452001 W
PLW13 0.18355000 W

F2 - Processing parameters
SI 32768
SF 176.0528575 MHz
WDW EM
SSB 0
LB 1.00 Hz
GB 0
PC 1.40

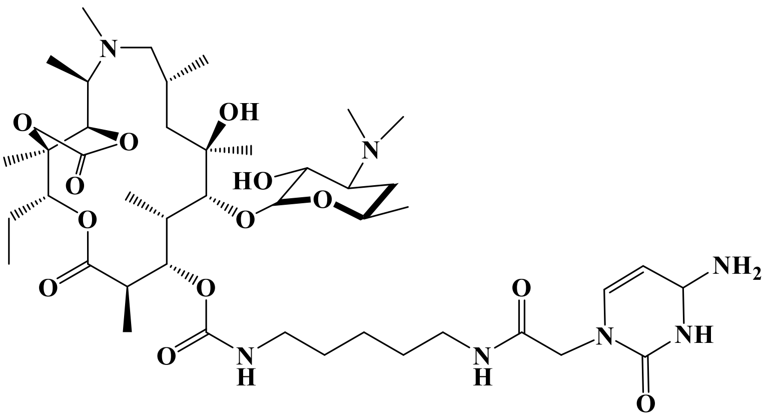

29C

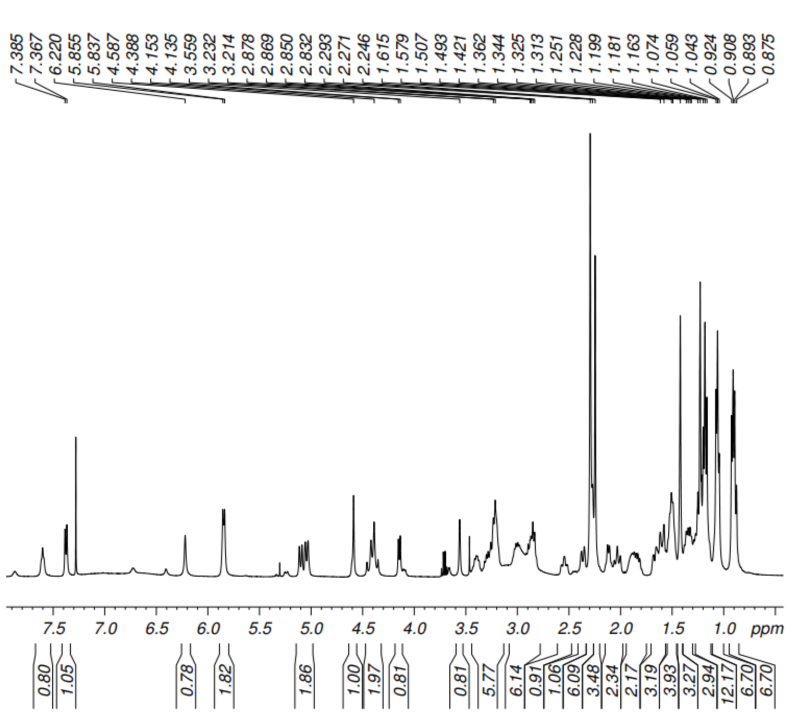

Current Data Parameters
NAME LXT-37-1
EXPNO 1
PROCNO 1

F2 - Acquisition Parameters
Date_ 20210630
Time 11.10 h
INSTRUM spect
PROBHD Z116098_0436 (
PULPROG zg30
TD 65536
SOLVENT CDCl3
NS 64
DS 2
SWH 8012.820 Hz
FIDRES 0.244532 Hz
AQ 4.0894465 sec
RG 30.51
DW 62.400 usec
DE 6.50 usec
TE 303.0 K
D1 1.00000000 sec
TD0 1
SFO1 400.1524709 MHz
NUC1 1H
P1 9.78 usec
PLW1 15.00300026 W

F2 - Processing parameters
SI 65536
SF 400.1500015 MHz
WDW EM
SSB 0
LB 0.30 Hz
GB 0
PC 1.00

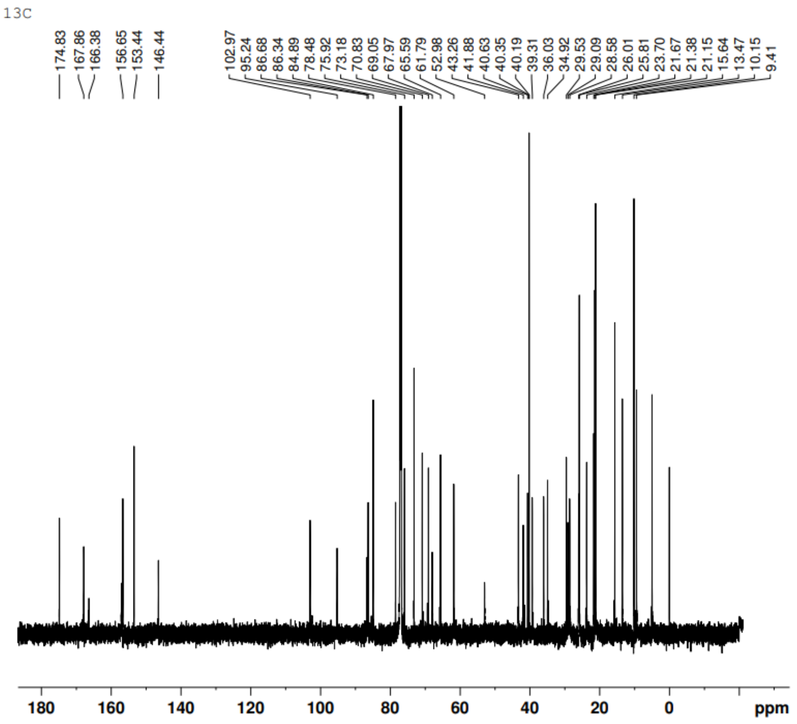

Current Data Parameters
NAME LXT-37-C
EXPNO 1
PROCNO 1

F2 - Acquisition Parameters
Date_ 20210730
Time 11.20 h
INSTRUM spect
PROBHD Z152837_0001 (
PULPROG zgpg30
TD 65536
SOLVENT CDCl3
NS 402
DS 4
SWH 42613.637 Hz
FIDRES 0.650232 Hz
AQ 0.7689557 sec
RG 187.62
DW 11.733 usec
DE 18.00 usec
TE 298.0 K
D1 2.00000000 sec
D11 0.03000000 sec
TD0 1
SFO1 176.0704628 MHz
NUC1 13C
P1 12.00 usec
PLW1 26.46899986 W
SFO2 700.1528006 MHz
NUC2 1H
CPDPRG[2 waltz16
PCPD2 65.00 usec
PLW2 10.69499969 W
PLW12 0.36452001 W
PLW13 0.18355000 W

F2 - Processing parameters
SI 32768
SF 176.0528575 MHz
WDW EM
SSB 0
LB 1.00 Hz
GB 0
PC 1.40

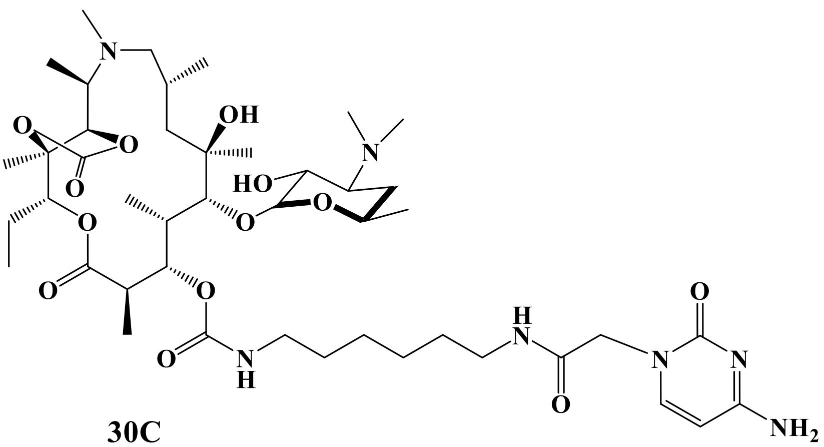

30C

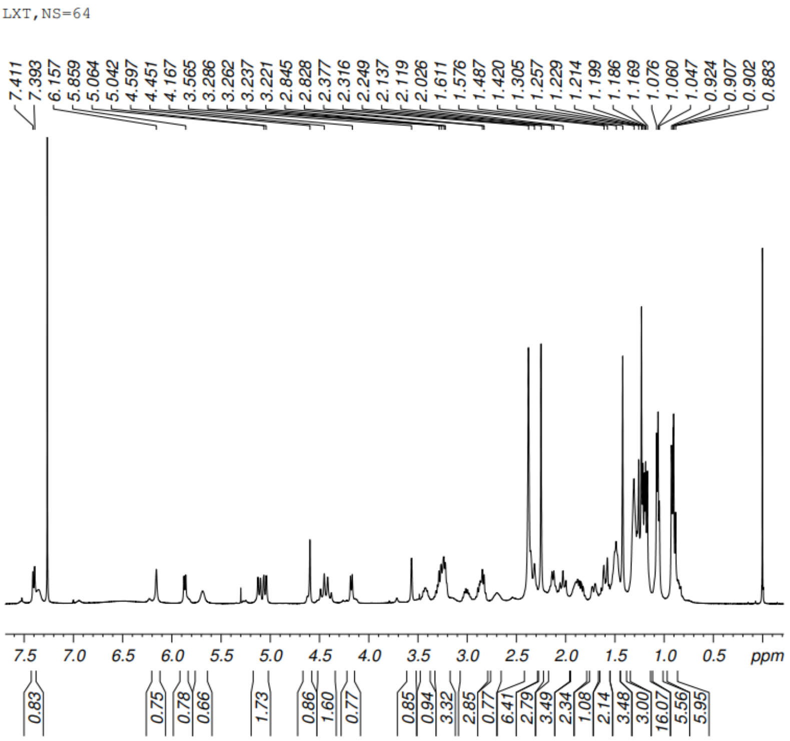

Current Data Parameters
NAME LXT-33-2
EXPNO 1
PROCNO 1

F2 - Acquisition Parameters
Date_ 20210609
Time 9.23 h
INSTRUM spect
PROBHD Z116098_0436 (
PULPROG zg30
TD 65536
SOLVENT CDCl3
NS 64
DS 2
SWH 8012.820 Hz
FIDRES 0.244532 Hz
AQ 4.0894465 sec
RG 111.98
DW 62.400 usec
DE 6.50 usec
TE 303.0 K
D1 1.00000000 sec
TD0 1
SFO1 400.1524709 MHz
NUC1 1H
P1 9.78 usec
PLW1 15.00300026 W

F2 - Processing parameters
SI 65536
SF 400.1500075 MHz
WDW EM
SSB 0
LB 0.30 Hz
GB 0
PC 1.00

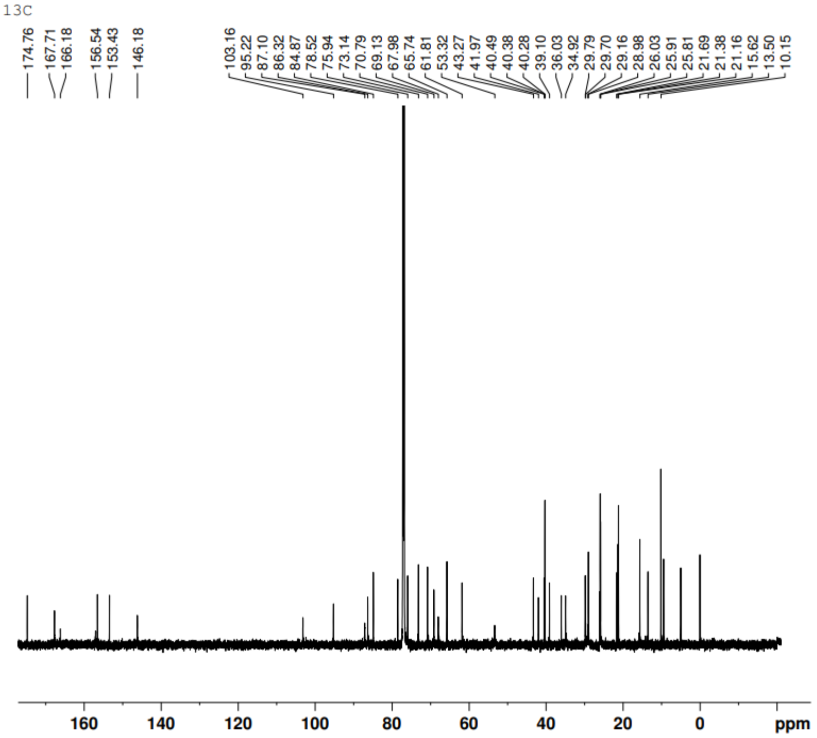

Current Data Parameters
NAME LXT-33-C
EXPNO 1
PROCNO 1

F2 - Acquisition Parameters
Date_ 20210730
Time 10.57 h
INSTRUM spect
PROBHD Z152837_0001 (
PULPROG zgpg30
TD 65536
SOLVENT CDCl3
NS 711
DS 4
SWH 42613.637 Hz
FIDRES 0.650232 Hz
AQ 0.7689557 sec
RG 187.62
DW 11.733 usec
DE 18.00 usec
TE 298.0 K
D1 2.00000000 sec
D11 0.03000000 sec
TD0 1
SFO1 176.0704628 MHz
NUC1 13C
P1 12.00 usec
PLW1 26.46899986 W
SFO2 700.1528006 MHz
NUC2 1H
CPDPRG[2 waltz16
PCPD2 65.00 usec
PLW2 10.69499969 W
PLW12 0.36452001 W
PLW13 0.18355000 W

F2 - Processing parameters
SI 32768
SF 176.0528575 MHz
WDW EM
SSB 0
LB 1.00 Hz
GB 0
PC 1.40

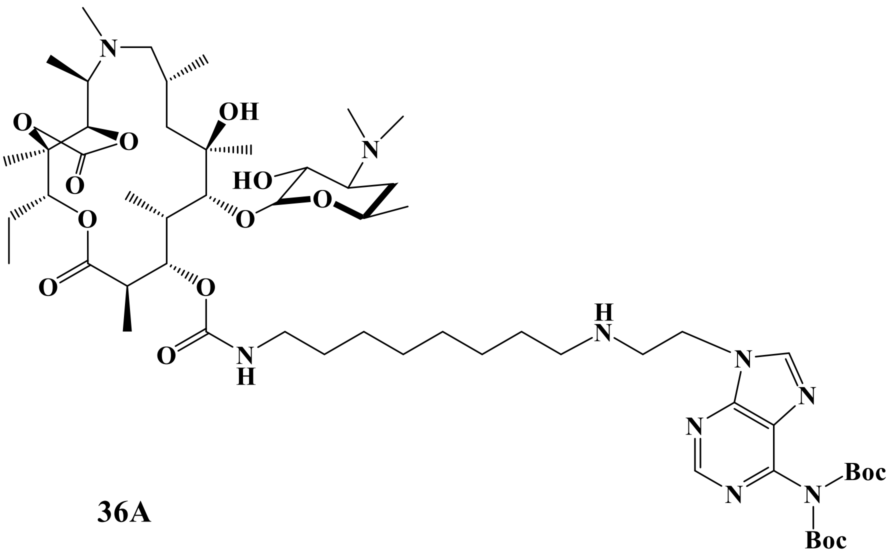

36A

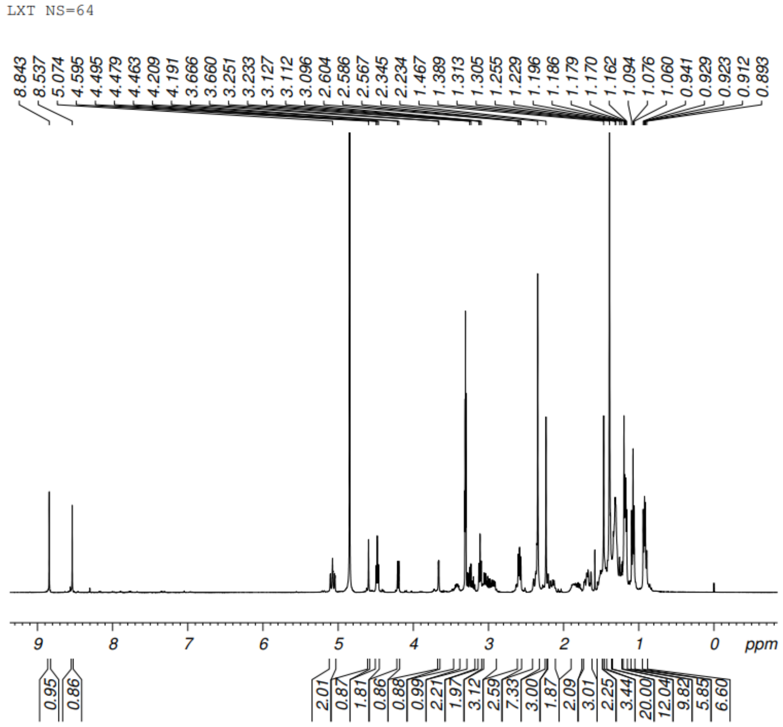

Current Data Parameters
NAME LXT-85
EXPNO 1
PROCNO 1

F2 - Acquisition Parameters
Date_ 20220422
Time 8.58 h
INSTRUM spect
PROBHD Z116098_0436 (
PULPROG zg30
TD 65536
SOLVENT MeOD
NS 64
DS 2
SWH 8012.820 Hz
FIDRES 0.244532 Hz
AQ 4.0894465 sec
RG 79.39
DW 62.400 usec
DE 6.50 usec
TE 298.0 K
D1 1.00000000 sec
TD0 1
SFO1 400.1524709 MHz
NUC1 1H
P1 9.78 usec
PLW1 15.00300026 W

F2 - Processing parameters
SI 65536
SF 400.1500095 MHz
WDW EM
SSB 0
LB 0.30 Hz
GB 0
PC 1.00

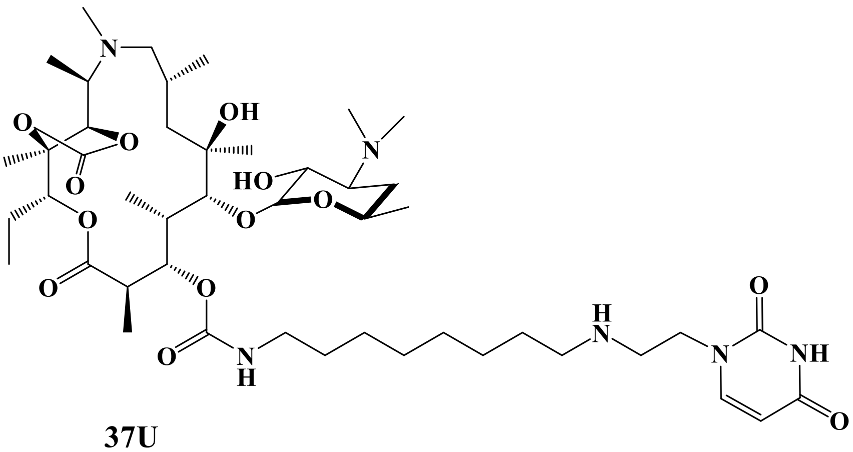

37U

LXT NS=64

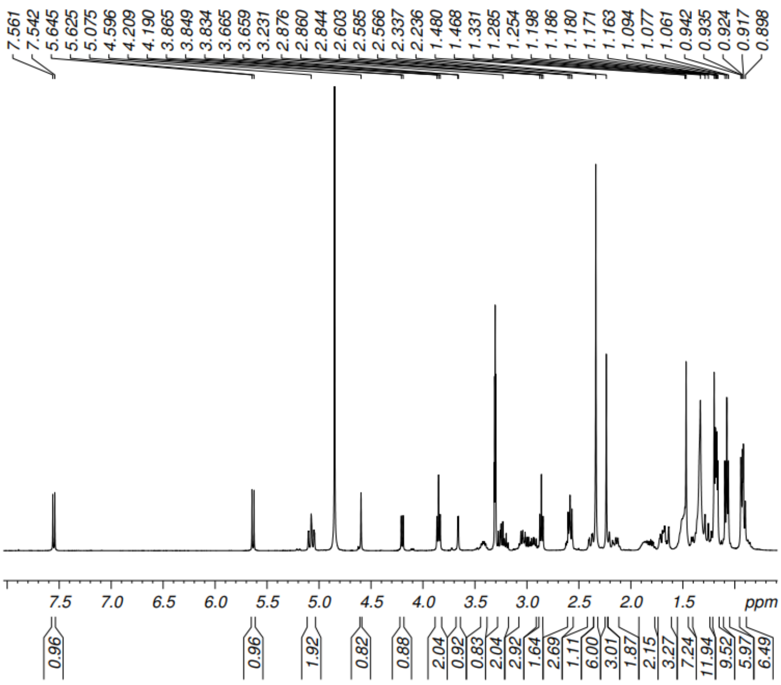

Current Data Parameters
NAME LXT-93
EXPNO 1
PROCNO 1

F2 - Acquisition Parameters
Date_ 20220501
Time 18.27 h
INSTRUM spect
PROBHD Z116098_0436 (
PULPROG zg30
TD 65536
SOLVENT MeOD
NS 64
DS 2
SWH 8012.820 Hz
FIDRES 0.244532 Hz
AQ 4.0894465 sec
RG 71.99
DW 62.400 usec
DE 6.50 usec
TE 298.0 K
D1 1.00000000 sec
TD0 1
SFO1 400.1524709 MHz
NUC1 1H
P1 9.78 usec
PLW1 15.00300026 W

F2 - Processing parameters
SI 65536
SF 400.1500099 MHz
WDW EM
SSB 0
LB 0.30 Hz
GB 0
PC 1.00

13C

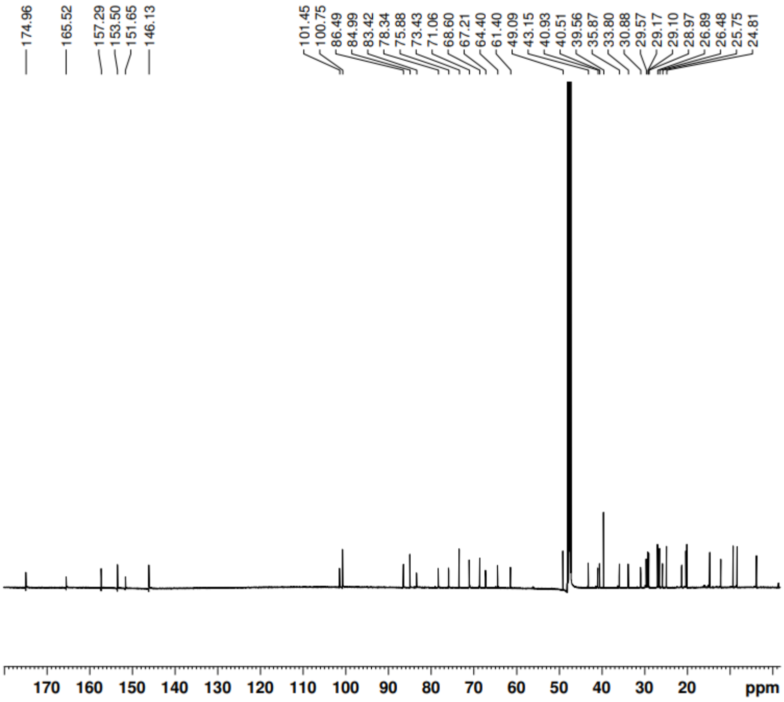

Current Data Parameters
NAME LXT-93
EXPNO 1
PROCNO 1

F2 - Acquisition Parameters
Date_ 20220505
Time 11.11 h
INSTRUM spect
PROBHD Z152837_0001 (
PULPROG zgpg30
TD 65536
SOLVENT MeOD
NS 1200
DS 4
SWH 42613.637 Hz
FIDRES 0.650232 Hz
AQ 0.7689557 sec
RG 187.62
DW 11.733 usec
DE 18.00 usec
TE 298.0 K
D1 2.00000000 sec
D11 0.03000000 sec
TD0 1
SFO1 176.0704628 MHz
NUC1 13C
P1 12.87 usec
PLW1 26.46899986 W
SFO2 700.1528006 MHz
NUC2 1H
CPDPRG[2 waltz16
PCPD2 65.00 usec
PLW2 10.69499969 W
PLW12 0.26130000 W
PLW13 0.13158000 W

F2 - Processing parameters
SI 32768
SF 176.0528575 MHz
WDW EM
SSB 0
LB 1.00 Hz
GB 0
PC 1.40

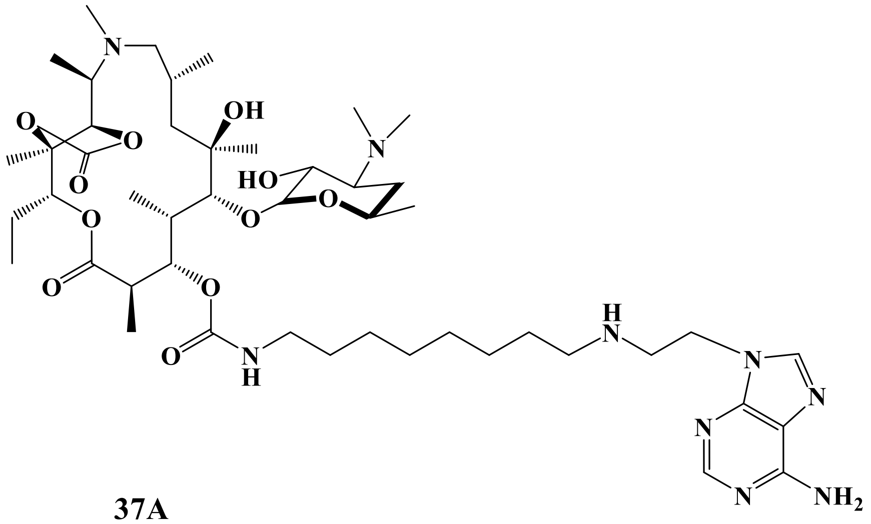

37A

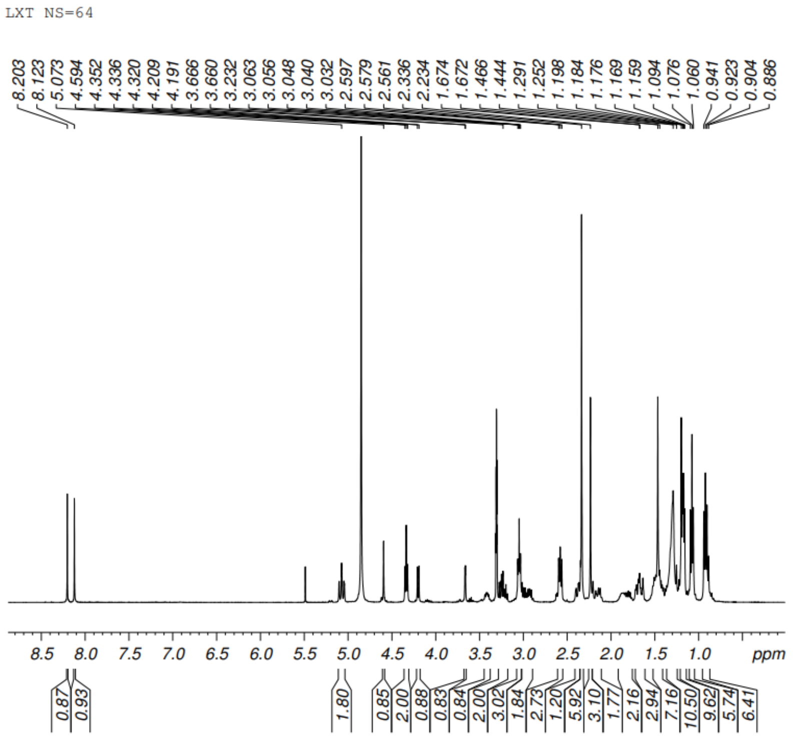

Current Data Parameters
NAME LXT-86
EXPNO 1
PROCNO 1

F2 - Acquisition Parameters
Date_ 20220419
Time 16.09 h
INSTRUM spect
PROBHD Z116098_0436 (
PULPROG zg30
TD 65536
SOLVENT MeOD
NS 64
DS 2
SWH 8012.820 Hz
FIDRES 0.244532 Hz
AQ 4.0894465 sec
RG 71.99
DW 62.400 usec
DE 6.50 usec
TE 298.0 K
D1 1.00000000 sec
TD0 1
SFO1 400.1524709 MHz
NUC1 1H
P1 9.78 usec
PLW1 15.00300026 W

F2 - Processing parameters
SI 65536
SF 400.1500091 MHz
WDW EM
SSB 0
LB 0.30 Hz
GB 0
PC 1.00

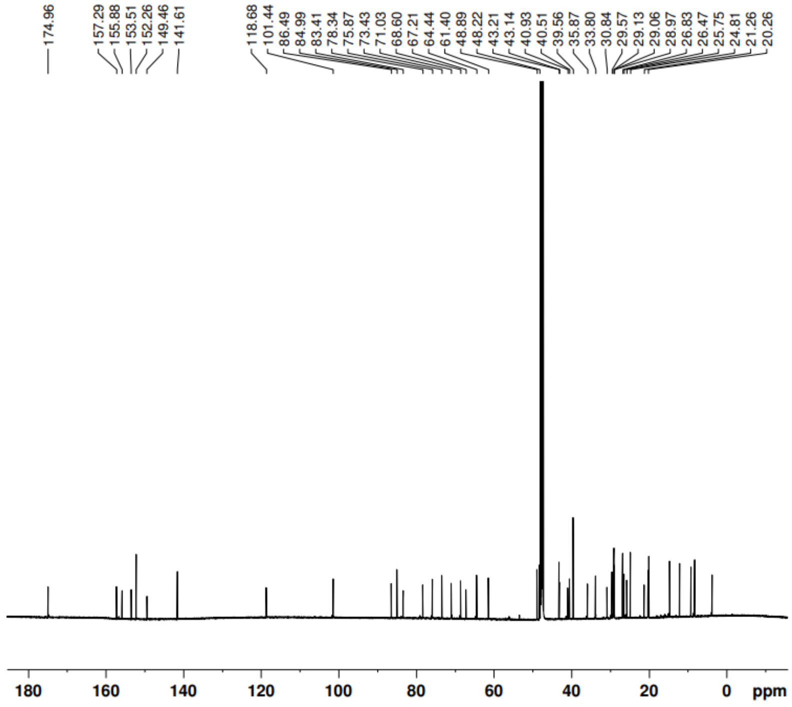

Current Data Parameters
NAME LXT-86
EXPNO 2
PROCNO 1

F2 - Acquisition Parameters
Date_ 20220421
Time 14.43 h
INSTRUM spect
PROBHD Z152837_0001 (
PULPROG zgpg30
TD 65536
SOLVENT MeOD
NS 1200
DS 4
SWH 42613.637 Hz
FIDRES 0.650232 Hz
AQ 0.7689557 sec
RG 187.62
DW 11.733 usec
DE 18.00 usec
TE 298.0 K
D1 2.00000000 sec
D11 0.03000000 sec
TD0 1
SFO1 176.0704628 MHz
NUC1 13C
P1 12.00 usec
PLW1 26.46899986 W
SFO2 700.1528006 MHz
NUC2 1H
CPDPRG[2 waltz16
PCPD2 65.00 usec
PLW2 10.69499969 W
PLW12 0.36452001 W
PLW13 0.18355000 W

F2 - Processing parameters
SI 32768
SF 176.0528575 MHz
WDW EM
SSB 0
LB 1.00 Hz
GB 0
PC 1.40
